# Preferences for community-based asynchronous-review eye clinics in the UK: A discrete choice experiment among patients, healthcare professionals, and the public

**DOI:** 10.1371/journal.pone.0342133

**Published:** 2026-09-15

**Authors:** Siyabonga Ndwandwe, Angus I. G. Ramsay, Josefine Magnusson, Steve Napier, Helen Baker, Jocelyn Cammack, Dun Jack Fu, Peng Tee Khaw, Sobha Sivaprasad, Hari Jayaram, Paul J. Foster, Caroline S. Clarke, on behalf of the HERCULES Consortium

**Affiliations:** 1 Research Department of Primary Care and Population Health, University College London, London, United Kingdom; 2 Research Department of Behavioural Science and Health, University College London, London, United Kingdom; 3 National Institute for Health and Care Research Biomedical Research Centre at Moorfields Eye Hospital NHS Foundation Trust and UCL Institute of Ophthalmology, London, United Kingdom; Touro University California College of Pharmacy, UNITED STATES OF AMERICA

## Abstract

The United Kingdom government’s 10 Year Health Plan for England sets out three shifts for a future-ready health service in England: shifting care from hospitals to communities, harnessing technology in health and care, and prioritising prevention over treatment. In this spirit, a large eye-hospital network in central London piloted a community-based, technician-led, asynchronous/virtual-review clinic to monitor stable chronic eye conditions and reduce avoidable harms, a model which could supplement traditional clinician-led services. Understanding stakeholder preferences around services is crucial for future planning and scaling. This paper describes a national survey examining key attributes for services monitoring chronic eye disease and calculating their relative importance for key stakeholders in the UK. We developed a discrete choice experiment (DCE) and distributed it electronically across the UK, targeting ophthalmology patients, healthcare professionals (HCP), and the public, asking all to respond from a patient’s perspective due to logistical and analytical considerations. Conditional logistic regression estimated the strength of stated preferences and trade-offs stakeholders would make between attributes, expressed as willingness to wait (WTW) longer than planned for their next appointment and willingness to travel (WTT) longer to the appointment location. Other attributes included whether a clinician or technician led the tests and how results were communicated (combined into single attribute reflecting service realities), venue accessibility by public transport, and parking. We received 389 responses, and the attribute describing who the patient saw and how results were received was the most important for all three groups. Compared to seeing a clinician with same-day results, respondents required appointment delay reductions of 6.27 months (95%CI 4.52–8.03) or travel reductions of 153 minutes (95%CI 88–219) if seeing a technician and receiving results by post, or smaller reductions if seeing technician then receiving results by phone. Differences across the respondent groups are discussed. This work suggests that technician-led asynchronous-review ophthalmology services could acceptably supplement clinician-led models, provided they improve appointment delays and reduce travel time. Therefore, service designers should align with these preferences when planning new services.

## Introduction

Ophthalmology accounts for the highest proportion of outpatient attendances across the English National Health Service (NHS), corresponding to 8% of activity in 2022/2023 [1]. Demand for ophthalmology outpatient services exceeds both workforce and infrastructure capacity, a common challenge across the NHS; despite an increase in employee numbers, the NHS vacancy rate increased from 7.9% in 2021 to 9.7% in 2022 [2]. Some ophthalmology patients have suffered appointment delays longer than one year, with some having suffered severe vision loss [3,4]. As highlighted in the 2024 Lord Darzi report, these service delays stem largely from austerity measures, underinvestment in infrastructure, workforce shortages, and the lasting effects of the COVID-19 pandemic [5].

At a traditional outpatient ophthalmology visit, tests and scans are performed by technicians, nurses, and other staff prior to a face-to-face consultation with a more senior clinician who provides the patient with their clinical results and recommends next steps, for example continued monitoring via regular follow-up. NHS England has recognised the potential for technician-led asynchronous-review clinics to contribute to increasing equitable access to services, and improving safety, throughput, and patient convenience [6]. In this delivery model, trained ophthalmic technicians perform tests, then specialist clinicians later review results remotely (virtually) and communicate results to patients via post or phone.

Asynchronous review clinics are not a new innovation in ophthalmology. Earlier iterations of the model include mobile diabetic retinopathy screening programmes introduced in Stockholm, Sweden, in 1990 and in Singapore in 2010 [7]. The UK has one of the largest and most successful diabetic retinopathy screening programmes, where trained technicians capture 45° colour fundus photographs that are later reviewed by clinicians; an approach that contributed to diabetic retinopathy no longer being the leading cause of blindness registration in the UK by 2010, for the first time in 50 years [7].

Potential benefits of asynchronous-review clinics compared to traditional outpatient department visits include shorter appointment times [8], lower cost per appointment [9,10], reduced waiting times for appointments [11], and more efficient use of senior clinicians’ time. Good clinical decision agreement between face-to-face appointments and asynchronous review clinics has been reported [12]. However, little is known about patients’ and other stakeholders’ preferences regarding these services. For specialties such as glaucoma and medical retinal disease, where regular monitoring is required to detect asymptomatic disease progression, aligning new service delivery models with stakeholder preferences can support greater acceptance. Higher acceptance might help reduce the appointment non-attendance, which was 6.9% in ophthalmology in 2021/22 compared with 6.5% across all other specialties [13].

In 2021, a large eye hospital network in central London piloted a community diagnostic hub in a shopping centre in north London. Previously healthcare-naïve individuals were trained as ophthalmic technicians, many with backgrounds in other consumer-facing roles such as retail. This service delivery model aligns with the UK government’s 10 Year Health Plan for England, which outlines three strategic shifts for a future-ready health service in England: shifting care from hospitals to communities, harnessing technology in health and care, and prioritising prevention over treatment [14]. In this work, we report UK stakeholders’ preferences around outpatient clinics for monitoring chronic eye disease and the relative importance of different service features. This is a crucial step in developing robust asynchronous clinic service models that are both patient- and staff-friendly and leverage recent advancements in technology and remote working practices.

## Methods

### Outline

We developed and administered an online discrete choice experiment (DCE) to assess preferences and trade-offs for diagnostic eye-service characteristics among patients, healthcare professionals (HCP) and the public. Respondents completed eight choice sets via an online written survey questionnaire (see S1 File). In each choice set, respondents chose between two hypothetical scenarios that included a mix of better and worse levels of the five service attributes so that neither scenario was clearly better than the other. The choice sets did not have an opt-out option, forcing respondents to trade off attribute levels in each choice set. Marginal rates of substitution of levels of one attribute for levels of others were calculated by analysing respondents’ sets of eight responses.

### Refining service attribute characteristics and developing their levels

The choice-set elements and their levels were developed via a multi-stage scoping exercise that was informed by the literature and refined using a survey and discussions with patient and public involvement (PPI) representatives, advocates and managers from the NHS Trust’s associated National Institute for Health and Care Research (NIHR) Biomedical Research Centre (BRC), and our multidisciplinary research team including clinicians, architects, PPI collaborators, and quantitative and qualitative researchers [15]. The first attribute encompassed two features: whether a technician or a clinician conducted the eye test and how results were communicated to patients. This reflected service realities as technicians do not interpret test results directly for patients. DCEs often include elements of cost or income in order to quantify ideas of willingness-to-pay for different levels of attributes; however, this was not considered meaningful for our study because the NHS is free at the point of use in the UK. Instead, we included concepts of willingness-to-wait and willingness-to-travel as proxy value elements. The former was characterised as the appointment delay attribute, reflecting the time gap between an expected follow-up appointment and the actual appointment date. For example, if the monitoring appointment frequency was recommended as every 6 months but one appointment had a 9-month gap, the delay for it would be 3 months. The latter was characterised as the time taken to reach the clinic venue from the respondent’s home. All attributes and their corresponding levels are given in Table 1.

**Table 1:** List of service attributes and corresponding levels.

Service attribute	Attribute levels
Who the patient sees on the day of their check-up and how results are given to the patient	Optometrist or doctor, with results given in person to the patient on the day
	Technician, with doctor reviewing results later and phoning the patient with results
	Technician, with doctor reviewing results later and sending results to the patient by post
Appointment delay	No delay – appointments happen when they should
	Up to 3 months
	Between 4 months and 8 months
	9 months or more
Accessibility by public transport	Short journey with no difficult connections
	Short journey with many or difficult connections
	Long journey with no difficult connections
	Long journey with many or difficult connections
Travel time	Less than 30 minutes
	30 minutes to 1 hour
	More than 1 hour but less than 1 hour and 30 minutes
	1 hour and 30 minutes or more
Parking availability	No parking available
	Parking available

### Experiment design

The survey was designed by the study team and implemented in an online platform by a third-party survey company. It was distributed and administered online and contained five sections (S1 File).Respondents were required to open the webpage containing the Participant Information Sheet (S2 File), which included information on what their participation would entail and how their data would be processed, before they could proceed to the rest of the survey. Participants were informed that by completing and submitting the questionnaire, they were giving their consent for the information provided to be used by the research team.

Section 1 of the electronic survey screened respondents for eligibility and recorded whether they identified as patients, HCP, or members of the public. To be eligible, respondents had to be at least 18 years old. Section 2 asked respondents to simply rank the five attributes in order, from 1 being most important to 5 being the least. Section 3 contained the eight choice sets, an example of which is shown in Table 2. Longer attribute descriptions were accessible in another window at any time during survey completion if the respondent wished. Respondents were required to complete sections 1–3 before progressing through the survey to avoid missingness in key components of the survey. Furthermore, in each choice set, respondents had to pick one option to proceed (forced-choice design) to the next choice set.

**Table 2:** Example of a choice set used in the discrete choice experiment.

	Option A	Option B
Who the patient sees on the day of the check-up and how results are given to the patient	Optometrist or doctor, with results given in person on the day	Technician, with doctor reviewing results later and phoning the patient with results
The time gap between an expected follow-up appointment and the actual appointment date	9 months or more	No gap – appointments happen when they should
How easy it is to reach the check-up location using public transport	Short journey with many or complex connections	Long journey with no complex connections
How long it takes to travel to the check-up location (door to door)	30 minutes to 1 hour	More than 1 hour but less than 1 hour and 30 minutes
Whether there is parking available at the check-up location	No parking available	Parking available
**Which location would you choose for your eye check-up?**		
	Option A ☐	Option B ☐

We implemented a D-optimal fractional design to optimise the survey using the *-dcreate-* module in Stata 17 [16], where we assumed non-informative prior estimates to select the optimal matrix [17]. We excluded unrealistic travel time and public transport accessibility level combinations *a priori*. For example, ‘less than 30 minutes’ could not be listed in the same scenario as ‘long journey with many or difficult connections’. The D-optimal design generated 16 choice sets that were split into two blocks of eight to balance participant cognitive burden against optimal data quality, consistent with current DCE approaches [18,19]. Respondents were randomly assigned one of the two blocks. Section 4 of the survey assessed respondents’ knowledge and attitudes towards key service characteristics while Section 5 captured socio-demographic characteristics.

The electronic survey was distributed nationally through UK eye-health charities (Macular Society, Glaucoma UK), the NHS Trust’s BRC PPI group, and the study team’s professional networks. The third-party survey company who hosted the survey also distributed the survey via their marketing partner. While participants could complete the survey online, by post, or by phone either in English or using an interpreter, all responses were ultimately electronic. The data were collected between 16 January 2023 and 13 March 2023. Only completed responses were provided to the research team; consequently, we do not have information on the number or characteristics of individuals who may have started but not completed the survey.

We asked all participants to respond from a patient’s perspective; we selected this approach for both practical and methodological reasons. Using a single patient-focused questionnaire across all respondent groups ensured consistency in attribute framing, wording, and choice tasks, reducing the risk that differences in responses would be driven by variations in survey design rather than underlying preferences. It also enabled direct comparison of how different stakeholder groups perceived patient preferences within a common decision-making framework; understanding the gap between what patients value and what stakeholders assume patients value is a key and crucial step in designing truly patient-centred care. In addition to participant responses, the survey platform provided information regarding start and end times of completion of the survey.

### Sample size calculation

Using Orme’s rule of thumb [20], our minimum total sample (*n*) was 78 per category, as derived using equation 1:


$$\begin{array}{c} n\geq \frac{\text{500 x c}}{\text{t x a}};n\geq \frac{\text{500 x }\text{4}}{\text{16 x }\text{2}}\geq \text{7}\text{8} \end{array}$$ (1)

where *c* = maximum number of attribute levels; *t* = number of choice sets; *a* = number of scenario options per choice set. We rounded up to target 300 respondents, i.e., 100 patients, 100 HCP, and 100 public, consistent with the literature [19,21–23].

### Ethical approval and consent to participate

This study was conducted in accordance with the Declaration of Helsinki, the principles of Good Clinical Practice (GCP), the UK Data Protection Act 2018, the UK Health Research Authority (HRA) Proportionate Review Service (PRS) procedures, the UK Policy Framework for Health and Social Care Research, and in accordance with the terms and conditions of the ethical approval given to the trial. Ethical approval for the overall HERCULES study was granted by North East – York Research Ethics Committee (Reference 21/NE/0164). Participants were informed that by completing and submitting the online questionnaire, they were giving their consent to participate in the study. They could withdraw at any point prior to submission without giving a reason. Furthermore, they were required to access the full Participant Information Sheet highlighting the purpose of the study, how their responses were going to be used, and assurance that their responses would be fully anonymised.

### Data analysis

Ranking data from section 2 of the survey were analysed by counting how often each attribute was placed first, second, third, fourth or last. Responses to attitudinal questions in section 4 of the survey were summarised as frequency and proportion. We used conditional logit regression in Stata 17 [16] to assess the strength of stated preferences from section 3. As the analysis was designed to compare preferences across the three predefined respondent groups (eye patients, HCP, and the public), rather than to investigate preference heterogeneity beyond these groups, conditional logit models were considered appropriate. Separate models were specified for each respondent group, allowing comparison of the direction and magnitude of preferences across groups.

This preference analysis is based on random utility (satisfaction) maximisation theory, which posits that in every choice set (*t*), each respondent (*i*) chooses the alternative (*j*) that yields the highest level of satisfaction (*U*) given the two alternatives. The general utility function *U*_*tij*_ decomposes into two components: a random term *ε*_*tij*_ and a deterministic component *V*_*tij*_ that is usually specified as a linear function and additive in utility [24]. This can be conceptualised as shown in equation 2:


$$\begin{array}{c} {\text{U}}_{\text{ijt}}={\text{V}}_{\text{tij}}+\varepsilon_{\text{ijt}}=\beta_{\text{tijk}}{\text{X}}_{\text{tijk}}+\varepsilon_{\text{ijt}} \end{array}$$ (2)

where *X* is a vector of *k* attributes and *β* is a coefficient vector associated with choice set *t*, respondent *i* and alternative *j*.

To calculate marginal rates of substitution (MRS), in terms of willingness to wait (WTW) and willingness to travel (WTT), we converted the corresponding categorical variables, appointment delay and travel time, into continuous variables using the midpoint values of the relevant attribute level in the base case. We assessed sensitivity to our use of the midpoint by using the minimum or maximum values in each of the categories in sensitivity analysis. See S3 Table for the full list of values used in each case. We excluded the constant term and set the confidence interval at 95%.

We first pooled all respondents into a single model, then conducted subgroup analyses by respondent type to assess patterns of preferences and trade-offs across the groups. Furthermore, we used seemingly unrelated estimation *(-suest-* Stata command) with cluster-robust standard errors and adjusted for clustering at the individual level to test for within-sociodemographic subgroups (see S4 Table). These subgroups included age group, gender, home ownership status, NHS region of residence, education level, and usual mode of transport used to access eye care diagnostic venue.

## Results

We received a total of 389 responses from 141 patients, 97 HCP, and 151 members of the public. The mean (standard deviation) time to complete the survey was 14.5 (26.3) minutes. Table 3 summarises respondents’ demographic characteristics. Further details including respondents’ attitudes to ophthalmology services in the NHS are given in the S6 Table. Overall, the gender split was even, with n = 200/389 (51%) females. The largest age bracket for eye patients was 65–74 years (n = 48/141, 34%), while for HCP and the public it was 35–44 years (n = 31/97, 32%) and 55–64 years (n = 43/151, 28%) respectively. Respondents’ ethnicity was mostly White for patients (n = 119/141, 84%) and public (n = 143/151, 95%), and just over half for HCP (56/97, 58%). Homeowners were in the majority in all three groups, at 112/141 (79%) in patients, 76/97 (78%) in HCP and 117/151 (77%) in public. Patients’ usual mode of transport was mostly public transport (n = 69/141, 49%) or driven by family or friends (n = 33/141, 23%), whereas HCP and public said they would mostly drive themselves to appointments (66/97, 68%; 96/151, 64%). Although the survey was distributed across the whole of the UK, most respondents resided in England outside London, followed by London, although the proportions of these varied across the groups, with more patients than HCPs or public being based in London. Rates of missing data were very low, so we used complete case analysis and did not impute missing values.

**Table 3:** Respondent demographics, by respondent group and overall.

	Eye patients n=141	HCP n=97	Public n=151	Total n=389
Age group (years)				
18-24 years	2 (1%)	0 (0%)	3 (2%)	5 (1%)
25-34 years	3 (2%)	12 (12%)	8 (5%)	23 (6%)
35-44 years	7 (5%)	31 (32%)	11 (7%)	49 (13%)
45-54 years	16 (11%)	28 (29%)	29 (19%)	73 (19%)
55-64 years	30 (21%)	19 (20%)	43 (28%)	92 (24%)
65-74 years	48 (34%)	6 (6%)	41 (27%)	95 (24%)
75-84 years	33 (23%)	0 (0%)	14 (9%)	47 (12%)
85 years and older	2 (1%)	0 (0%)	2 (1%)	4 (1%)
Prefer not to say	0 (0%)	1 (1%)	0 (0%)	1 (0%)
Missing	0 (0%)	0 (0%)	0 (0%)	0 (0%)
Gender				
Female	69 (49%)	56 (58%)	75 (50%)	200 (51%)
Male	71 (50%)	40 (41%)	75 (50%)	186 (48%)
Other	0 (0%)	0 (0%)	0 (0%)	0 (0%)
Prefer not to say	1 (1%)	1 (1%)	0 (0%)	2 (1%)
Missing	0 (0%)	0 (0%)	1 (1%)	1 (0%)
Ethnicity				
Asian or Asian British	5 (4%)	26 (27%)	4 (3%)	35 (9%)
Black, Black British, Caribbean, African	6 (4%)	5 (5%)	1 (1%)	12 (3%)
Mixed or multiple ethnic groups	4 (3%)	2 (2%)	1 (1%)	7 (2%)
White	119 (84%)	56 (58%)	143 (95%)	318 (82%)
Other ethnicity	1 (1%)	2 (2%)	0 (0%)	3 (1%)
Prefer not to say	4 (3%)	5 (5%)	2 (1%)	11 (3%)
Missing	2 (1%)	1 (1%)	0 (0%)	3 (1%)
Education level				
Degree or postgraduate degree	81 (57%)	86 (89%)	67 (44%)	234 (60%)
HNC/HND (or equivalent)	0 (0%)	0 (0%)	0 (0%)	0 (0%)
A level (or equivalent)	29 (21%)	5 (5%)	31 (21%)	65 (17%)
GCSE (or equivalent)	12 (9%)	3 (3%)	38 (25%)	53 (14%)
Other level of education	5 (4%)	2 (2%)	2 (1%)	9 (2%)
Other qualification	0 (0%)	0 (0%)	0 (0%)	0 (0%)
No formal qualification	7 (5%)	1 (1%)	9 (6%)	17 (4%)
No qualification	0 (0%)	0 (0%)	0 (0%)	0 (0%)
Prefer not to say	4 (3%)	0 (0%)	2 (1%)	6 (2%)
Missing	3 (2%)	0 (0%)	2 (1%)	5 (1%)
Home ownership status				
Own their home	112 (79%)	76 (78%)	117 (77%)	305 (78%)
Rent privately	13 (9%)	3 (3%)	15 (10%)	31 (8%)
Rent from local authority	5 (4%)	13 (13%)	14 (9%)	32 (8%)
Other living arrangements	7 (5%)	3 (3%)	0 (0%)	10 (3%)
Prefer not to say	2 (1%)	1 (1%)	4 (3%)	7 (2%)
Missing	2 (1%)	1 (1%)	1 (1%)	4 (1%)
Occupation status				
Full time employed	29 (21%)	64 (66%)	45 (30%)	138 (35%)
Part time employed	16 (11%)	22 (23%)	21 (14%)	59 (15%)
Homemaker	3 (2%)	1 (1%)	9 (6%)	13 (3%)
Student (in education)	1 (1%)	0 (0%)	2 (1%)	3 (1%)
Retired	81 (57%)	3 (3%)	66 (44%)	150 (39%)
Unemployed and seeking work	0 (0%)	1 (1%)	2 (1%)	3 (1%)
Unemployed, health related	7 (5%)	0 (0%)	2 (1%)	9 (2%)
Other employment status	1 (1%)	3 (3%)	2 (1%)	6 (2%)
Prefer not to say	2 (1%)	2 (2%)	1 (1%)	5 (1%)
Missing	1 (1%)	1 (1%)	1 (1%)	3 (1%)
Usual mode of transport for appointments				
Use public transport	69 (49%)	15 (15%)	28 (19%)	112 (29%)
Self-drive	28 (20%)	66 (68%)	96 (64%)	190 (49%)
Driven by family/friend	33 (23%)	6 (6%)	20 (13%)	59 (15%)
Use taxi/cab-haling service	2 (1%)	2 (2%)	2 (1%)	6 (2%)
Other means	6 (4%)	7 (7%)	5 (3%)	18 (5%)
Prefer not to say	0 (0%)	0 (0%)	0 (0%)	0 (0%)
Missing	3 (2%)	1 (1%)	0 (0%)	4 (1%)
Self-reported overall health				
Very good	18 (13%)	34 (35%)	22 (15%)	74 (19%)
Good	92 (65%)	57 (59%)	95 (63%)	244 (63%)
Poor	24 (17%)	5 (5%)	28 (19%)	57 (15%)
Very poor	4 (3%)	0 (0%)	1 (1%)	5 (1%)
Prefer not to say	2 (1%)	1 (1%)	3 (2%)	6 (2%)
Missing	1 (1%)	0 (0%)	2 (1%)	3 (2%)
Living with health conditions*				
Yes	104 (74%)	24 (25%)	57 (38%)	185 (48%)
No	34 (24%)	66 (68%)	89 (59%)	189 (49%)
Prefer not to say	3 (2%)	7 (7%)	5 (3%)	15 (4%)
Place of Residence in UK				
Scotland	10 (7%)	5 (5%)	11 (7%)	26 (7%)
Wales	3 (2%)	0 (0%)	9 (6%)	12 (3%)
Northern Ireland	2 (1%)	3 (3%)	1 (1%)	6 (2%)
England – Northeast & Yorkshire	9 (6%)	13 (13%)	26 (17%)	48 (12%)
England – Northwest	8 (6%)	11 (11%)	16 (11%)	35 (9%)
England – Midlands	15 (11%)	21 (22%)	21 (14%)	57 (15%)
England – East of England	9 (6%)	6 (6%)	13 (9%)	28 (7%)
England – Southeast	25 (18%)	10 (10%)	31 (21%)	66 (17%)
England – Southwest	6 (4%)	12 (12%)	14 (9%)	32 (8%)
London – Northwest London	14 (10%)	2 (2%)	3 (2%)	19 (5%)
London – Central London	10 (7%)	4 (4%)	2 (1%)	16 (4%)
London – East London	6 (4%)	0 (0%)	0 (0%)	6 (2%)
London – Southeast London	14 (10%)	2 (2%)	3 (2%)	19 (5%)
London – Southwest London	10 (7%)	2 (2%)	0 (0%)	12 (3%)
Outside the UK	0 (0%)	4 (4%)	0 (0%)	4 (1%)
Prefer not to say	0 (0%)	2 (2%)	0 (0%)	2 (1%)
Missing	0 (0%)	0 (0%)	1 (1%)	1 (0%)

NVQ = National Vocational Qualification, HND = Higher National Diploma, HNC = Higher National Certificate, GCSE = General Certificate of Secondary Education, HCP = health care professional

* See S11 Table for a further breakdown by condition among the 185 respondents who reported living with a health condition.

### Simple attribute ranking

**Fig 1:**
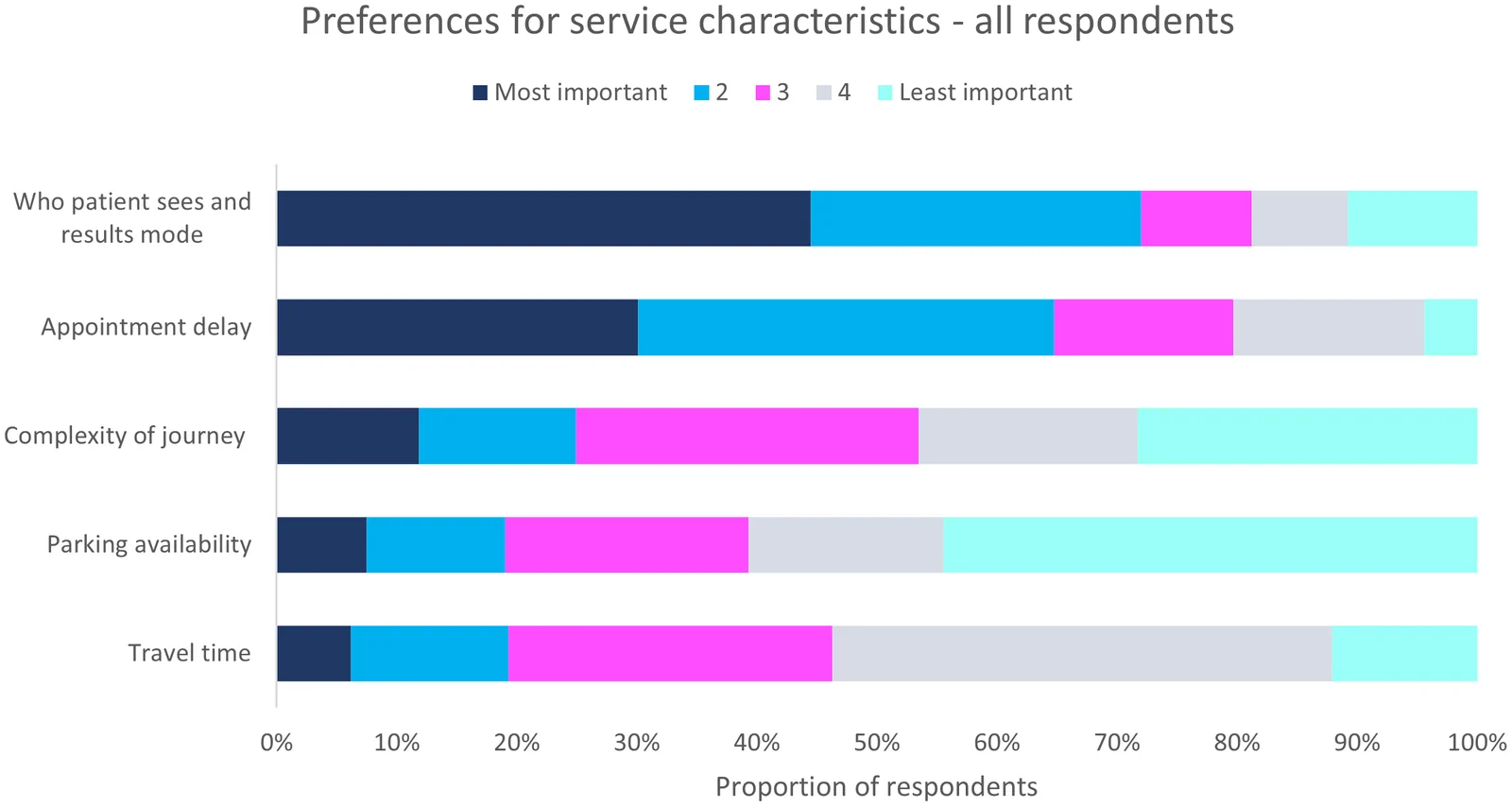
Attribute ranking (all respondents).

The composite attribute describing who the patient sees on the day and the mode of results communication was ranked as the most important service attribute, while appointment delays were ranked second (Fig 1). Parking availability was least important. This was consistent for respondents who lived in and outside London. Results were consistent across respondent groups (see S5 Fig).

A simple ranking of the five attributes showed that 44% (173/389) felt that the composite attribute describing who patient sees on the day and mode of results communication was the most important and 44% (173/389) felt that parking availability was the least important of the five.

### Discrete choice experiment

The conditional logit regression (all respondents) showed that shorter appointment delays, shorter travel times, having a clinician conduct the test and same-day results instead of a technician and later results, greater accessibility by public transport and better parking availability were all preferred, confirming face validity of the experiment. Full results are given in S7 Table. The public was statistically indifferent between seeing an optometrist or doctor and getting same-day results (reference category) versus seeing a technician then receiving results by phone. However, eye patients and HCP had statistically different preferences (p < 0.001) where seeing a technician and receiving results by post corresponded with the lowest level of utility relative to seeing an optometrist or doctor and receiving results on the same day. Furthermore, travel time did not significantly influence patients’ preferences at the 95% confidence level, whereas it was a statistically significant determinant of preferences among healthcare professionals and members of the public (see S7 Table).

The MRS estimation including all respondents showed the required trade-off in appointment delays would be a reduction by 6.27 months (95% CI, 4.52 to 8.03) for respondents to accept seeing a technician and receiving results by post compared to seeing an optometrist and getting same-day results (reference category); however, this changed to a required reduction by 3.39 months (95% CI, 2.00 to 4.78) if results were received by phone after seeing a technician (Table 4). When considering respondent types separately, patients required a reduction by 7.29 months (95%CI, 4.25 to 10.32) in order to accept seeing a technician and receiving results by post compared to seeing an optometrist or doctor and receiving same-day results (Table 4). Regarding public transport journeys, respondents (overall group) were willing to wait 5.53 months (95% CI, 3.80 to 7.26) longer for their appointment to have a short journey with no difficult connections or 3.92 months (95% CI, 2.30 to 5.53) to have a short journey with many or difficult connections, compared in each case to a long journey with many or complex journey (reference category) (Table 4). The WTT analysis highlighted similar preference patterns to the WTW, as it was based on the same utility estimation (equation 2). The public required a reduction in travel time (73 minutes) to balance seeing a technician and getting results by post vs. the reference category, while patients required a 305-minute reduction (Table 5). Note: patients had very wide WTT confidence intervals around this reduction because travel time did not significantly influence their preferences (see S7 Table).

**Table 4:** Respondents’ willingness to wait longer for their appointment (months) to balance a change in level in the other service attributes.

		Willingness To Wait, months (mean (95% CI))			
		All Respondents n = 389	Eye Patients n = 141	HCP n = 97	Public n = 151
Who the patient sees on the day of their check-up and how results are given to the patient	Optometrist or doctor, with results given in person on the day	*Ref*	*Ref*	*Ref*	*Ref*
	Technician, with doctor reviewing results later and phoning with results	−3.39 (−4.78, −2.00)	−5.59 (−8.12, −3.06)	−3.99 (−6.80, −1.17)	−0.68 (−2.68, 1.32)
	Technician, with doctor reviewing results later and sending results by post	−6.27 (−8.03, −4.52)	−7.29 (−10.32, −4.25)	−7.84 (−11.62, −4.06)	−4.07 (−6.60, −1.55)
Accessibility by public transport	Long journey with many or difficult connections	*Ref*	*Ref*	*Ref*	*Ref*
	Long journey with no difficult connections	2.12 (0.68, 3.57)	2.37 (−0.19, 4.92)	0.03 (−2.64, 2.69)	3.26 (0.99, 5.53)
	Short journey with many or difficult connections	3.92 (2.30, 5.53)	3.51 (0.77, 6.25)	2.07 (−0.91, 5.06)	5.61 (2.89, 8.32)
	Short journey with no difficult connections	5.53 (3.80, 7.26)	5.49 (2.56, 8.43)	4.08 (1.27, 6.89)	6.44 (3.37, 9.51)
Parking availability	Parking available	*Ref*	*Ref*	*Ref*	*Ref*
	No parking available	−4.76 (−6.05, −3.48)	−1.88 (−3.52, −0.24)	−5.63 (−8.12, −3.15)	−6.98 (−9.54, −4.41)
Travel time	Travel time (minutes)	−0.04 (−0.06, −0.02)	−0.02 (−0.05, 0.00)	−0.05 (−0.09, −0.01)	−0.06 (−0.09, −0.02)

HCP = healthcare professionals; CI = confidence interval; n = number of respondents; Ref = reference category

**Table 5:** Respondents’ willingness to travel for longer (in minutes) to balance a change in level in the other service attributes.

		Willingness To Travel, minutes (mean (95%CI))			
		All Respondents n = 389	Eye Patients n = 141	HCP n = 97	Public n = 151
Who the patient sees on the day of their check-up and how results are given to the patient	Optometrist or doctor, with results given in person on the day	*Ref*	*Ref*	*Ref*	*Ref*
	Technician, with doctor reviewing results later and phoning with results	−84 (−127, −40)	−233 (−488, 20)	−82 (−156, −9)	−12 (−47, 23)
	Technician, with doctor reviewing results later and sending results by post	−153 (−219, −88)	−305 (−628, 18)	−163 (−287, −38)	−73 (−124, −21)
Accessibility by public transport	Long journey with many or difficult connections	*Ref*	*Ref*	*Ref*	*Ref*
	Long journey with no difficult connections	49 (6, 93)	99 (−60, 258)	0 (−54, 56)	58 (7, 110)
	Short journey with many or difficult connections	97 (42, 153)	147 (−57, 351)	43 (−23, 110)	100 (36, 164)
	Short journey with no difficult connections	134 (69, 199)	229 (−34, 493)	84 (0, 169)	115 (43, 187)
Parking availability	Parking available	*Ref*	*Ref*	*Ref*	*Ref*
	No parking available	−117 (−166, −67)	−78 (−180, 23)	−117 (−207, −26)	−125 (−189, −60)
Delay days	Appointment delay (days)	−24 (−34, −14)	−41 (−85, 2)	−20 (−36, −4)	−17 (−27, −7)

HCP = healthcare professionals; CI = confidence interval; n = number of respondents; Ref = reference category.

Sensitivity analyses evaluating the robustness of the results to instead using the maximum or minimum points when converting categorical variables to continuous variables are given in S8 and S9 Tables for the WTW and WTT analyses, respectively. These cases provided similar results to those obtained for the base case (midpoint), with overlapping confidence intervals around the coefficients across the sensitivity analyses.

Regarding differences across demographic subgroups, the directions of preferences were similar across subgroups, but results suggested that preference magnitudes were statistically different at the 95% confidence level between respondents who: i) resided in NHS regions in London vs NHS regions outside London, ii) owned their homes vs those that did not, iii) were in full-time employment vs retired, iv) held at least a first degree vs non-degree holders, v) used public transport vs not use public transport to reach their eye appointment venues (see S4 Table).

## Discussion

Our findings have highlighted that technician-led asynchronous/virtual-review ophthalmology services could acceptably supplement clinician-led models provided they improve appointment delays and reduce travel time. To the best of our knowledge, this is the first DCE to explicitly consider non-clinical technicians and the practicalities of delivering services via asynchronous-review clinics when assessing stakeholders’ preferences for accessing outpatient ophthalmology services. Combining the attributes of who the patient sees on the day of their check-up and how results are given to the patient into a composite attribute was crucial for meaningful results. As patients would typically evaluate these aspects as a package when considering service options, we aimed to estimate preferences for realistic service configurations rather than isolate the marginal effects of clinician type and communication method. This combined attribute was ranked in this analysis as most important by patients, HCP, and the public alike, when answering questions from the perspective of a patient. This agrees with previous research that indicated the person the patient saw was an important attribute for glaucoma patients in Australia [25], and other work involving glaucoma patients in Nottingham, UK, [26], that found that travel time and who the patient saw were important, although this latter analysis did not include non-clinical technicians as possible service providers, only opticians and doctors.

The trade-offs highlight a higher level of disutility or dissatisfaction associated with not speaking to someone when receiving results. To balance seeing a non-clinical technician and receiving results later by post, respondents required their appointment delay to be reduced by 6.27 months versus a 3.39-month reduction in delay if receiving results by phone. Whilst this interpretation implies that appointments are delayed, from a service design perspective, we want to quantify the reduction in delay needed to make technician-led models acceptable, and not in endorsing longer delays. Patients’ concern over the lack of direct contact with clinicians (ophthalmologists or optometrists) in technician-led ophthalmic diagnostic pathways is noted in the literature [26,27]. Nonetheless, there are constraints to the willingness to wait; patients would not be advised to simply wait for longer to ensure seeing a clinician, as the longer the appointment is delayed past their recommended follow-up time, the greater the risk of their developing worse disease before the next monitoring appointment. Also, workforce shortages and infrastructure limitations, for example limited space and the desire to have fewer stable patients attending the main hospital to assist with infection control post-COVID, mean that not all patients can attend the main hospital site to see an optometrist or ophthalmologist at every monitoring appointment. Hence the movement towards technician-led asynchronous clinics, often located away from the main hospital site.

There were several limitations in the study. Firstly, the attributes and levels presented to respondents were a balance between depicting important attributes for patients and minimising cognitive burden as informed by the D-optimal design. We chose not to include dominance or repeated choice checks, firstly as our design emphasised minimising cognitive burden while maintaining statistical efficiency through a D‑optimal fractional design and adding additional tasks would have increased task complexity and potentially fatigued respondents and, secondly, because there is evidence in the literature suggesting these tasks are unreliable screening tests and are costly in terms of statistical power [28–31]. The second key limitation is the hypothetical nature of DCEs, and we could not directly verify respondents’ understanding and engagement with the online survey. While the average completion time was approximately 15 minutes, in line with our expectations, this measure alone does not guarantee meaningful engagement with the survey content.

Thirdly, respondents’ socioeconomic status might not be representative of the general UK population. For example, 79% of respondents owned their own homes compared to 62.5% national average in England and Wales [32]. Similarly, 60% of our respondents had a degree or postgraduate degree compared to 33% in England and Wales [33]. Fourth, there could potentially be underlying heterogeneity in each respondent group that could shape preferences. For example, preferences for non-eye-patient respondents who have experienced eye pain might differ from those who have not. However, this would require a larger sample size to permit clearer understanding of these important characteristics. This sample size limitation precludes discussion of the brief subgroup exploratory analysis presented in the Supplementary Materials. Finally, the values of WTT and WTW estimates should be interpreted with caution, as DCEs have well-known framing issues. For example, the MRS might not change proportionally if, for example, categories of: less than 45 mins / 45–90 mins / 90–180 mins / more than 180 mins had been used, instead of: less than 30 mins / 30–60 mins / 60–90 mins / more than 90 mins. Also, WTT estimates were larger than the range offered in the DCE, though this is consistent with other findings in the literature [25].

## Conclusion

This analysis suggests a strong preference for seeing a clinician and getting results on the same day, although respondents were willing to see a technician and receive results later by post if their appointment delay was reduced by 6.27 months, or by 3.39 months if they received results by phone. This trade-off highlights the opportunity for adopting technician-led models for asynchronous-review services in the NHS as a potentially patient-centred, equitable, cost-effective model that allows stable patients to be seen sooner. This is an important finding in the context of implementing services delivery models that address the huge delays across specialties currently experienced in the English NHS, owing to the aftermath of the COVID-19 pandemic and the preceding years of austerity.

## Supporting information

S1 File Full DCE survey. (DOCX)

S2 File Participant Information Sheet. (DOCX)

S3 Table Categorical to continuous. (DOCX)

S4 Table Within-group comparisons. (DOCX)

S5 Fig Attribute ranking by respondent type. (DOCX)

S6 Table Respondent attitudes. (DOCX)

S7 Table Outputs of conditional logit. (DOCX)

S8 Table Sensitivity WTW. (DOCX)

S9 Table Sensitivity WTT. (DOCX)

S10 File Members HERCULES Consortium. (DOCX)

S11 Table Additional Health Status Qs. (DOCX)
