# Supplementary material for: Preferences for community-based asynchronous-review eye clinics in the UK: A discrete choice experiment among patients, healthcare professionals, and the public
Source: PLoS One. 2026 Sep 15;21(9):e0342133. doi: 10.1371/journal.pone.0342133 (PMC13577492; doi:10.1371/journal.pone.0342133)
Supplement: Supplementary file 1 [file pone.0342133.s001.docx]

**Preferences for community-based asynchronous-review eye clinics in the UK: a discrete choice experiment among patients, healthcare professionals, and the public**

**Author information:**

Siyabonga Ndwandwe ^1*^, Angus IG Ramsay ^2^, Josefine Magnusson^2^, Steve Napier ^3‡^, Helen Baker ^3^, Jocelyn Cammack ^3^, Dun Jack Fu ^3^, Peng Tee Khaw ^3^, Sobha Sivaprasad ^3^, Hari Jayaram 3, Paul J Foster ^3^, Caroline S Clarke ^1^ on behalf of the HERCULES Consortium^

**^1^** Research Department of Primary Care and Population Health, University College London, London, United Kingdom

^2^ Research Department of Behavioural Science and Health, University College London, London, United Kingdom

^3^ National Institute for Health and Care Research Biomedical Research Centre at Moorfields Eye Hospital NHS Foundation Trust and UCL Institute of Ophthalmology, London, United Kingdom

^*^Corresponding author

e-mail: [s.ndwandwe@ucl.ac.uk](mailto:s.ndwandwe@ucl.ac.uk)

^‡^ Patient and public involvement representative

^ Membership of the HERCULES Consortium is provided in the Supporting Information.

# **S 1. The full survey for the Discrete Choice Experiment**

Please note that “If you have any problems…” and “You can alter the font size…” were repeated on every page. The background and font could also be adjusted on any page.

You can alter the font size and background colour by clicking on these buttons.

*HERCULES is supported by the National Institute for Health and Care Research (NIHR) Moorfields Biomedical Research Centre, and commercial partners: Zeiss, Optos, and Ubisense Ltd*.

You may contact the IQVIA helpline on 0800 XXX XXXX or via textphone on 0800 XXX XXXX, The Next button is at the bottom right of each page.

*If you have any problems filling out this questionnaire, please call our helpline on 0800* XXX XXXX*.*

*.*

**Project HERCULES: Healthcare Exemplar for Recovery From COVID-19 By Use of Linear Examination Systems**

**This information is about this survey that you may decide to take, to help with the HERCULES project.**

**Why do we need your help?**

We are developing a new, more efficient and sustainable system for eye health checks that will have people seen more quickly and treated sooner, and for some people this could reduce the risk of blindness. The information you provide will be vital in helping us understand how to design this new system, which could be used by other areas of healthcare within the NHS.

**What we would like you to do**

In the following pages we have listed some descriptions of services where patients with a stable long-term eye condition might go for their regular eye check-ups to monitor their condition and diagnose any changes in it. You will see pairs of hypothetical scenarios, and for each of these pairs we would like you to tell us which option sounds best to you. It is possible that in some of the pairs neither of the two options exactly match your preferences, but please don’t worry about this.

**Completing the questionnaire**

Please follow the instructions and complete all of the questions in each section using the spaces provided. There is no right or wrong answer. Your responses should reflect your views as a patient or potential patient. We know some of the choices might be hard, and you might sometimes have to choose the least bad option rather than choosing one which you would actually like. Please just do your best.

**Taking part in this survey is voluntary. Please note you must be aged 18 years or over to take part. The research team will not be able to link your responses back to you in any way. IQVIA is the company administering the survey on behalf of the research team.**

You may complete this questionnaire over the phone by contacting IQVIA on 0800 XXX XXXX or email helRline-QH@igvia.com or via textphone on 0800 XXX XXXX. You may also contact the helpline to request an interpreter to help you answer the questions in another language or to request a paper copy or large print version to be sent to you.

**What will happen to your data**

**By completing this questionnaire, you are giving your consent for the following:**

- You are providing data to IQVIA via this questionnaire. IQVIA will not use your data for any other purpose than this specific study, and will not analyse your data.
- You will not be asked to provide any information that identifies you whilst completing the questionnaire online. Once you have submitted your answers, all the responses will be anonymised by IQVIA. After this you will not be able to ask for your data to be removed because it will not be possible to work out which data is yours.
- Fully anonymous completed questionnaires will then be sent by IQVIA to University College London, UK, where they will be analysed by researchers who are part of the HERCULES team.
- Nothing will be linked to any other health care information about you that is held by any health care provider, at any point.

**Please click here to read the full Participant Information Sheet (PIS) before proceeding.**

*[see S 2 for the PIS]*

Please then close the PIS window to return to this survey.

**SECTION 1**

Please can you answer these questions:

**Which of these choices best describes you (please tick one box)?**

☐ Eye patient (accesses or has accessed hospital eye services)

☐ Healthcare professional (in any specialty). If yes, do you work in:

☐Ophthalmology

☐Other

If other specialty, please specify in this box:

☐ Member of the public (everyone else)

**If you are an eye patient, what is your main eye condition (please tick one):**

☐ Glaucoma

☐ Diabetic retinopathy

☐ Macular degeneration

☐ Other eye condition

If other eye condition, please specify in this box:

**What is your age group (please tick one box)?**

☐ Under 18 years

*(Please note, respondents under 18 years of age are not eligible to complete the survey.)*

*[survey closes and thanks respondent if they indicate that they are under 18 years of age]*

☐ 18-24 years

☐ 25-34 years

☐ 35-44 years

☐ 45-54 years

☐ 55-64 years

☐ 65-74 years

☐ 75-84 years

☐ 85 years and older

☐ Prefer not to say (but over 18 years)

**SECTION 2**

Please consider the following features of services where patients with a stable long-term eye condition might go for their regular eye check-ups. You can find more detailed information here if you wish, and you can refer back to this information tab later on during the survey via links on the following pages.

**Please rank the five features according to their overall importance to you:**

**Please use 1, 2, 3, 4, 5 in the order that you like (1 = most important, 5 = least important), and do not use any number more than once.**

| Who the patient *sees* on the day of their check-up and how results are given to the patient  *(Level of seniority of health care professional)* | □ |
| --- | --- |
| The time gap between an expected follow-up appointment and the actual appointment date  *(For example, if the next appointment is due in 6 months but is booked for 9 months later, the time gap is 3 months)* | □ |
| How easy it is to reach the check-up location using public transport  *(Based on the distance and difficulty of the journey)* | □ |
| How long it takes to travel to the check-up location (door to door) | □  □ |
| Whether there is parking available at the check-up location |  |

**SECTION 3**

In the following tables, the column on the left describes the different features and they are the same for each question. The other 2 columns show 2 different options, Option A. and Option B, that are different each time, and we would like you to please pick your preferred option for each pair. Each choice should be considered as a standalone question and does not relate to any other choices made elsewhere in the survey.

***Click here for more information on service features***

*[this opens a window with “more detailed information”. See it at the end of this section]*

**Question 1**

|  |  | Option A | Option B | |
| --- | --- | --- | --- | --- |
| Who the patient sees on the day of their check-up and how results are given to the patient | | **Technician, with doctor reviewing results later and phoning the patient with results** | **Technician, with doctor reviewing results later and sending results to the patient by post** |  |
| The time gap between an expected follow-up appointment and the actual appointment date | | **Between 4 months and 8 months** | **Up to 3 months** |  |
| How easy it is to reach the check-up location using public transport | | **Short distance with no difficult connections** | **Long distance with no difficult connections** |  |
| How long it takes to travel to the check-up location (door to door) | | **Less than 30 minutes** | **1 hour and 30 minutes or more** |  |
| Whether there is parking available at the check-up location | | **No parking available** | **Parking available** |  |

| **Which location would you choose for your eye check-up?** | | |
| --- | --- | --- |
|  | Option A  ☐ | Option B  ☐ |

**Question 2**

|  | Option A | Option B |
| --- | --- | --- |
| Who the patient sees on the day of their check-up and how results are given to the patient | **Optometrist or doctor, with results given in person on the day** | **Technician, with doctor reviewing results later and phoning the patient with results** |
| The time gap between an expected follow-up appointment and the actual appointment date | **Between 4 months and 8 months** | **No gap – appointments happen when they should** |
| How easy it is to reach the check-up location using public transport | **Long distance with no difficult connections** | **Short distance with many or difficult connections** |
| How long it takes to travel to the check-up location (door to door) | **Less than 30 minutes** | **30 minutes to 1 hour** |
| Whether there is parking available at the check-up location | **Parking available** | **No parking available** |

| **Which location would you choose for your eye check-up?** | | |
| --- | --- | --- |
|  | Option A  ☐ | Option B  ☐ |

**Question 3**

|  | Option A | Option B |
| --- | --- | --- |
| Who the patient sees on the day of their check-up and how results are given to the patient | **Optometrist or doctor, with results given in person on the day** | **Technician, with doctor reviewing results later and phoning the patient with results** |
| The time gap between an expected follow-up appointment and the actual appointment date | **9 months or more** | **Between 4 months and 8 months** |
| How easy it is to reach the check-up location using public transport | **Short distance with no difficult connections** | **Long distance with no difficult connections** |
| How long it takes to travel to the check-up location (door to door) | **1 hour and 30 minutes or more** | **More than 1 hour but less than 1 hour and 30 minutes** |
| Whether there is parking available at the check-up location | **No parking available** | **Parking available** |

| **Which location would you choose for your eye check-up?** | | |
| --- | --- | --- |
|  | Option A  ☐ | Option B  ☐ |

**Question 4**

|  | Option A | Option B |
| --- | --- | --- |
| Who the patient sees on the day of their check-up and how results are given to the patient | **Optometrist or doctor, with results given in person on the day** | **Technician, with doctor reviewing results later and phoning the patient with results** |
| The time gap between an expected follow-up appointment and the actual appointment date | **9 months or more** | **Up to 3 months** |
| How easy it is to reach the check-up location using public transport | **Long distance with no difficult connections** | **Short distance with many or difficult connections** |
| How long it takes to travel to the check-up location (door to door) | **More than 1 hour but less than 1 hour and 30 minutes** | **30 minutes to 1 hour** |
| Whether there is parking available at the check-up location | **No parking available** | **Parking available** |

| **Which location would you choose for your eye check-up?** | | |
| --- | --- | --- |
|  | Option A  ☐ | Option B  ☐ |

**Question 5**

|  | Option A | Option B |  |
| --- | --- | --- | --- |
| Who the patient sees on the day of their check-up and how results are given to the patient | **Technician, with doctor reviewing results later and phoning the patient with results** | **Technician, with doctor reviewing results later and sending results to the patient by post** | |
| The time gap between an expected follow-up appointment and the actual appointment date | **Up to 3 months** | **No gap – appointments happen when they should** | |
| How easy it is to reach the check-up location using public transport | **Long distance with many or difficult connections** | **Short distance with many or difficult connections** | |
| How long it takes to travel to the check-up location (door to door) | **1 hour and 30 minutes or more** | **30 minutes to 1 hour** | |
| Whether there is parking available at the check-up location | **Parking available** | **No parking available** | |

| **Which location would you choose for your eye check-up?** | | |
| --- | --- | --- |
|  | Option A  ☐ | Option B  ☐ |

**Question 6**

|  | Option A | Option B |
| --- | --- | --- |
| Who the patient sees on the day of their check-up and how results are given to the patient | **Technician, with doctor reviewing results later and sending results to the patient by post** | **Optometrist or doctor, with results given in person on the day** |
| The time gap between an expected follow-up appointment and the actual appointment date | **9 months or more** | **No gap – appointments happen when they should** |
| How easy it is to reach the check-up location using public transport | **Short distance with many or difficult connections** | **Short distance with no difficult connections** |
| How long it takes to travel to the check-up location (door to door) | **Less than 30 minutes** | **More than 1 hour but less than 1 hour and 30 minutes** |
| Whether there is parking available at the check-up location | **No parking available** | **Parking available** |

| **Which location would you choose for your eye check-up?** | | |
| --- | --- | --- |
|  | Option A  ☐ | Option B  ☐ |

**Question 7**

|  |  | Option A | Option B |
| --- | --- | --- | --- |
| Who the patient sees on the day of their check-up and how results are given to the patient | | **Technician, with doctor reviewing results later and phoning the patient with results** | **Technician, with doctor reviewing results later and sending results to the patient by post** |
| The time gap between an expected follow-up appointment and the actual appointment date | | **No gap – appointments happen when they should** | **Between 4 months and 8 months** |
| How easy it is to reach the check-up location using public transport | | **Long distance with no difficult connections** | **Long distance with many or difficult connections** |
| How long it takes to travel to the check-up location (door to door) | | **Less than 30 minutes** | **More than 1 hour but less than 1 hour and 30 minutes** |
| Whether there is parking available at the check-up location | | **No parking available** | **Parking available** |

| **Which location would you choose for your eye check-up?** | | |
| --- | --- | --- |
|  | Option A  ☐ | Option B  ☐ |

**Question 8**

|  |  | Option A | Option B |  |
| --- | --- | --- | --- | --- |
| Who the patient sees on the day of their check-up and how results are given to the patient | | **Technician, with doctor reviewing results later and phoning the patient with results** | **Optometrist or doctor, with results given in person on the day** | |
| The time gap between an expected follow-up appointment and the actual appointment date | | **Between 4 months and 8 months** | **9 months or more** | |
| How easy it is to reach the check-up location using public transport | | **Short distance with no difficult connections** | **Long distance with no difficult connections** | |
| How long it takes to travel to the check-up location (door to door) | | **1 hour and 30 minutes or more** | **30 minutes to 1 hour** | |
| Whether there is parking available at the check-up location | | **No parking available** | **Parking available** | |

| **Which location would you choose for your eye check-up?** | | |
| --- | --- | --- |
|  | Option A  ☐ | Option B  ☐ |

***Pop-out window showing more detailed information about attributes:***

**Who the patient sees on the day of their check-up and how results are given to the patient**

Possible descriptions:

- Optometrist or doctor, with results given in person to the patient on the day
- Technician, with doctor reviewing results later and sending results to the patient by post
- Technician, with doctor reviewing results later and phoning the patient with results

**The time gap between an expected follow-up appointment and the actual appointment date** *For example, if the next appointment is due in 6 months but is actually booked for 9 months later, the time gap is 3 months*

Possible descriptions:

- No gap – appointments happen when they should
- Up to 3 months
- Between 4 months and 8 months
- 9 months or more

**How easy it is to reach the check-up location using public transport based on how long and how difficult that journey is**

Possible descriptions:

- Short distance with no difficult connections
- Short distance with many or difficult connections
- Long distance with no difficult connections
- Long distance with many or difficult connections

**How long it should take to travel to the check-up location (door to door)**

Possible descriptions:

- Less than 30 minutes
- 30 minutes to 1 hour
- More than 1 hour but less than 1 hour and 30 minutes
- 1 hour and 30 minutes or more

**Whether there is parking available at the check-up location**

Possible descriptions:

- No parking available
- Parking available

**SECTION 4**

1. Please say how easy or difficult it was to make your choices in the previous section.

☐ Very easy

☐ Easy

☐ Neither easy nor difficult

☐ Difficult

☐ Very difficult

☐ Prefer not to say

2. Please tell us what was easy or difficult about making the choices in the previous section, and provide any other comments on making the choices, in this box:

**Thinking about the current system of eye care, how do you feel about the following statements?**

3. How long do you think it should take a typical patient to travel to their regular eye check- up appointment?

☐ 30 minutes

☐ 60 minutes

☐ 90 minutes

☐ 120 minutes

☐ Don’t know / Prefer not to say

4. The quality of eye health services varies significantly across the different NHS regions.

☐ Strongly disagree

☐ Disagree

☐ Neither agree nor disagree

☐ Agree

☐ Strongly agree

☐ Don’t know / Prefer not to say

5. There is a relationship between eye health and overall health.

☐ Strongly disagree

☐ Disagree

☐ Neither agree nor disagree

☐ Agree

☐ Strongly agree

☐ Don’t know / Prefer not to say

6. I am satisfied with the skill and competency of the staff providing eye healthcare.

☐ Strongly disagree

☐ Disagree

☐ Neither agree nor disagree

☐ Agree

☐ Strongly agree

☐ Don’t know / Prefer not to say

7. The staff providing eye healthcare are friendly and sensitive to cultural differences.

☐ Strongly disagree

☐ Disagree

☐ Neither agree nor disagree

☐ Agree

☐ Strongly agree

☐ Don’t know / Prefer not to say

8. I am open to having some appointments with my eye doctor over the phone or via video call instead of in person.

☐ Strongly disagree

☐ Disagree

☐ Neither agree nor disagree

☐ Agree

☐ Strongly agree

☐ Don’t know / Prefer not to say

**SECTION 5**

**Finally, we would like to ask a few questions about you.**

We would like to ask you some questions about your health and personal circumstances. All your responses will be fully anonymised before being used in the analysis. This information will help us analyse the data and report the results in a useful and appropriate way.

1. How would you describe your health in general?

☐ Very good

☐ Good

☐ Poor

☐ Very poor

☐ Prefer not to say

2. Do you have any health conditions?

☐ Yes

☐ No

☐ Prefer not to say

a) If yes, how would you describe your health condition? (Please tick all that apply, or choose “Prefer not to say”)

☐ Long-term eye condition

☐ Non-long term eye condition

☐ Other long-term condition

☐ Other non-long-term condition

If other long-term conditions, please specify in this box

If other non-long-term conditions, please specify in this box

☐. Prefer not to say

3. If you have an eye condition, how often are you asked to go for a routine eye health check-up about this condition?

☐ Once every 2-6 months

☐ Once every 7-12 months

☐ Once every 13-18 months

☐ Once every longer than 18 months

☐ Prefer not to say

4. If you have an eye condition, do you have regular eye injections?

☐ Yes

☐ No

☐ Prefer not to say

5. How do you usually travel from where you live to where you get health care services?

☐ I use public transport

☐ I drive myself

☐ A family member or friend drives me

☐ I use a taxi/cab-hailing service

☐ Other means of transport

If other, please specify in this box

☐ Prefer not to say

6. What is your gender?

☐ Female

☐ Male

☐ Other

If other, please specify in this box

☐ Prefer not to say

7. Which of the following best describes your ethnicity?

☐ Asian or Asian British

☐ Black, Black British, Caribbean, or African

☐ Mixed or multiple ethnic groups

☐ White

☐ Other ethnicity

If other, please specify in this box

☐ Prefer not to say

8. In which NHS region do you currently live?

☐ Scotland

☐ Wales

☐ Northern Ireland

☐ England – Northeast and Yorkshire

☐ England – Northwest

☐ England – Midlands

☐ England – East of England

☐ England – Southeast

☐ England – Southwest

☐ London – Northwest London

☐ London – Central London

☐ London – East London

☐ London – Southeast London

☐ London – Southwest London

☐ Outside the UK *(please specify)*

☐ Prefer not to say

9. Which of the following best describes your highest completed level of education?

☐ Degree or postgraduate degree

☐ A level (or equivalent)

☐ GCSE (or equivalent)

☐ No formal academic or vocational qualification

☐ Other level of education

If other, please specify in this box

☐ Prefer not to say

10. Which of the following best describes your employment status?

☐ Full time employed

☐ Part time employed

☐ Homemaker

☐ Student (in education)

☐ Retired

☐ Unemployed and seeking work

☐ Unemployed – unable to work for health reasons

☐ Other employment status

If other, please specify in this box

☐ Prefer not to say

11. Which of the following best describes your living arrangements?

☐ I own my home

☐ I rent from local authority/housing association

☐ I rent privately

☐ Other living arrangements

If other *(e.g., living with friends or family),* please specify below

☐ Prefer not to say

**Thank you for taking the time to fill in this questionnaire. We very much appreciate your time and your responses.**

**If you have any questions or suggestions about this questionnaire, please contact IQVIA over the phone on 0800 XXX XXXX, or by emailing helpline-QH@iqvia.com or via textphone on 0800 XXX XXXX.**

If you are interested in learning more about the HERCULES project, you can visit this link:

<https://tinyurl.com/yckskudn>

*If you have any problems filling out this questionnaire, please call our helpline on 0800* XXX XXXX*.*
