# Supplementary material for: Preferences for community-based asynchronous-review eye clinics in the UK: A discrete choice experiment among patients, healthcare professionals, and the public
Source: PLoS One. 2026 Sep 15;21(9):e0342133. doi: 10.1371/journal.pone.0342133 (PMC13577492; doi:10.1371/journal.pone.0342133)
Supplement: Supplementary file 2 [file pone.0342133.s002.docx]

**Preferences for community-based asynchronous-review eye clinics in the UK: a discrete choice experiment among patients, healthcare professionals, and the public**

**Author information:**

Siyabonga Ndwandwe ^1*^, Angus IG Ramsay ^2^, Josefine Magnusson^2^, Steve Napier ^3‡^, Helen Baker ^3^, Jocelyn Cammack ^3^, Dun Jack Fu ^3^, Peng Tee Khaw ^3^, Sobha Sivaprasad ^3^, Hari Jayaram 3, Paul J Foster ^3^, Caroline S Clarke ^1^ on behalf of the HERCULES Consortium^

**^1^** Research Department of Primary Care and Population Health, University College London, London, United Kingdom

^2^ Research Department of Behavioural Science and Health, University College London, London, United Kingdom

^3^ National Institute for Health and Care Research Biomedical Research Centre at Moorfields Eye Hospital NHS Foundation Trust and UCL Institute of Ophthalmology, London, United Kingdom

^*^Corresponding author

e-mail: [s.ndwandwe@ucl.ac.uk](mailto:s.ndwandwe@ucl.ac.uk)

^‡^ Patient and public involvement representative

^ Membership of the HERCULES Consortium is provided in the Supporting Information.

# **S 2. Participant Information Sheet**

**Participant Information Sheet**

**Healthcare Exemplar for Recovery from COVID-19 by Use of Linear Examination Systems**

**Project HERCULES – Analysis of Stakeholder Preferences (Discrete Choice Experiment)**

**Chief Investigators:**

Professor Paul Foster, Consultant Ophthalmologist, Moorfields Eye Hospital

Professor Sobha Sivaprasad, Consultant Ophthalmologist, Moorfields Eye Hospital

Dr Hari Jayaram, Consultant Ophthalmologist, Moorfields Eye Hospital

Project HERCULES: Stakeholder Preferences (Discrete Choice Experiment); PIS v1.1 – IRAS ID: 303760 16 Nov 2022

You are being invited to take part in this research study because you are either a patient receiving eye care within the NHS, a healthcare professional, or a member of the public. Before you decide to participate it is important for you to understand why the research is being done and what it will involve.

Please read the following information carefully. Please talk to others about the study if you wish.

**What is the purpose of this study?**

The primary aim of Project HERCULES is to develop a cost-effective, patient-centred, and sustainable new model for providing NHS outpatient care in the “post pandemic era”. We plan to develop and innovate socially distanced NHS outpatient care provided in different configurations in a retail space at Brent Cross Shopping Centre in London, with aim of maximizing the safety, efficiency, and patient experience.

In this part of the study, we would like to seek the opinions of patients, health care professionals, and the public to understand their preferences for the different characteristics of diagnostic eye care services.

This will enable us to incorporate this feedback to help inform the development of future innovations in Diagnostic Hubs for Eye Care across the NHS.

**Why have I been invited to take part?**

You have been invited to take part because you are either a patient receiving eye care within the NHS, a health care professional, or a member of the public.

**Do I have to take part?**

It is up to you to decide to join the study. We will describe this specific part of the HERCULES study in this information sheet. If you agree to take part, we will then ask you to complete an online survey. This will be administered by IQVIA which is a professional survey company. If you complete the survey, we will take this as you are providing your consent to take part in the study. You are free to withdraw at any time, without giving a reason.

**What will happen to me if I take part?**

IQVIA, which is a professional survey company, is hosting the survey on behalf of the project team. Once you close this window, you can continue to the next page to fill in the survey via this online platform. If you prefer, you can contact IQVIA to arrange to receive the survey by post, or to complete it over the telephone (either in English, or using an interpreter arranged by IQVIA).

In the survey, we will list some descriptions of services where patients with a stable long-term eye condition might go for their regular eye check-ups. You will see pairs of options, and we are asking you to think about these various descriptions and tell us which option sounds best to you. There is no right or wrong answer. Your responses should reflect your views as a patient or potential patient. We will also ask you for some basic information about yourself.

Analysis of the completed survey data, which will include no identifiable information and not be linked to any other information, will be carried out by our colleagues at University College London.

**Expenses and payments**

There will be no expenses provided for this study, as no additional visits to the hospital are necessary.

**What will I have to do?**

Once you have had time to decide whether or not to take part, we would like you to access the questionnaires via the online platform, or you can request a paper version or to complete them over the telephone.

**What are the possible disadvantages and risks of taking part?**

There are no specific risks to taking part in this study.

**What are the possible benefits of taking part?**

This study will not help you directly, but the information we obtain from this study will help us to develop, evaluate and deliver a new, efficient, and patient centred system of care, that will help the NHS safely and effectively look after patients with chronic eye diseases in the “post pandemic era”.

**What will happen if I don’t want to carry on with the study?**

You can withdraw from the study at any time, by exiting from the online platform. However, information that has already been collected may still be used.

**What if there is a problem?**

This is very unlikely in an online survey such as this. However, should you have any concerns you may contact the Moorfields Eye Hospital Patient Advice and Liaison Service by telephone on 020 XXX XXXX or by emailing [moorfields.pals@nhs.net](mailto:moorfields.pals@nhs.net). If you have any questions or suggestions about the questionnaire itself, please contact IQVIA on Freephone 0800 XXX XXXX or email [helpline-QH@iqvia.com](mailto:helpline-QH@iqvia.com).

**How will we use information about you?**

You will be providing data to IQVIA via the online platform. IQVIA will not use your data for any other purpose than this specific study and will not analyse your data.

You will not be asked to provide any information that identifies you whilst completing the questionnaire online.

Once you have submitted your answers, you will not be able to ask for your data to be removed from the dataset for analysis because it would be impossible to work out which was your data.

The final anonymised dataset (from which it will not be possible to identify any individual’s responses) will be sent by IQVIA to researchers at University College London and analysed by the researchers there, who are also part of the HERCULES study team.

Nothing will be linked to any other health care information about you that is held by any health care provider, at any point. We will write our reports in a way that no-one can work out that you took part in the study.

If you contact the IQVIA helpline to request a paper copy or a call back from a translator, they will ask you for your name and address or telephone number in order to arrange this. The responses you provide to the questions, in any format, will not be linked back to your personal details. Personal details will be destroyed 3 months after the end of the project.

**What are your choices about how your information is used?**

You can stop being part of the study at any time, without giving a reason, by exiting from the online platform, but we will keep any anonymous information about you that you have already provided.

**Where can you find out more about how your information is used?**

You can find out more about how we use your information by contacting IQVIA on Freephone 0800 XXX XXXX or email [helpline-XX@XXX.com](mailto:helpline-XX@XXX.com).

Moorfields Eye Hospital NHS Foundation Trust is the sponsor for this study, which is based only in the United Kingdom, and will act as the data controller for this study. This means that we are responsible for looking after your information and using it properly. IQVIA will delete identifiable information about you after the study has finished.

**What will happen to the results of the research study?**

The results of the study will be presented at national and international conferences and published in scientific journals. All data for analysis will be anonymous and none of the participants involved the study can be identified from any reports published. We will also share the results of this study with NHS England, the National Institute for Health and Care Research, Moorfields Eye Charity, the Macular Society and Glaucoma UK. Should you wish to see the results of the study in the future, please let one of our team know.

**Who is organising and funding the research?**

This project is being funded through support from the National Institute for Health and Care Research Biomedical Research Centre at Moorfields Eye Hospital and the UCL Institute of Ophthalmology, and commercial partners Zeiss, Optos and Ubisense. The project is sponsored by Moorfields Eye Hospital NHS Foundation Trust.

**Who has reviewed the study?**

All research in the NHS is looked at by an independent group of people called a Research Ethics Committee, to protect your interests. This study has been reviewed and given favourable opinion by the North East – York NHS Research Ethics Committee.

**Further information and contact details**

You have the right to ask questions concerning your participation in this study at any time. You may ask questions before you sign the consent, at any time during your participation in the study, and after you are finished with the study.

The people to contact for any questions or problems during the study are listed below:

IQVIA: Freephone 0800 XXX XXXX or email [helpline-QH@iqvia.com](mailto:helpline-QH@iqvia.com).

If you have any concerns regarding the conduct of the study, or wish to speak to someone independent of the research team, please contact the Patient Advice and Liaison Service (PALS) on 020 XXX XXXX.

Thank you for taking the time for reading the study details in this Participant Information Sheet.

Please close this tab to return to the survey
