# Supplementary material for: Preferences for community-based asynchronous-review eye clinics in the UK: A discrete choice experiment among patients, healthcare professionals, and the public
Source: PLoS One. 2026 Sep 15;21(9):e0342133. doi: 10.1371/journal.pone.0342133 (PMC13577492; doi:10.1371/journal.pone.0342133)
Supplement: Supplementary file 3 [file pone.0342133.s003.docx]

**Preferences for community-based asynchronous-review eye clinics in the UK: a discrete choice experiment among patients, healthcare professionals, and the public**

**Author information:**

Siyabonga Ndwandwe ^1*^, Angus IG Ramsay ^2^, Josefine Magnusson^2^, Steve Napier ^3‡^, Helen Baker ^3^, Jocelyn Cammack ^3^, Dun Jack Fu ^3^, Peng Tee Khaw ^3^, Sobha Sivaprasad ^3^, Hari Jayaram 3, Paul J Foster ^3^, Caroline S Clarke ^1^ on behalf of the HERCULES Consortium^

**^1^** Research Department of Primary Care and Population Health, University College London, London, United Kingdom

^2^ Research Department of Behavioural Science and Health, University College London, London, United Kingdom

^3^ National Institute for Health and Care Research Biomedical Research Centre at Moorfields Eye Hospital NHS Foundation Trust and UCL Institute of Ophthalmology, London, United Kingdom

^*^Corresponding author

e-mail: [s.ndwandwe@ucl.ac.uk](mailto:s.ndwandwe@ucl.ac.uk)

^‡^ Patient and public involvement representative

^ Membership of the HERCULES Consortium is provided in the Supporting Information.

# **S 3. Table. Values used for minimum, mid and maximum values of categorical attributes when transformed into continuous variables**

| **Service attribute** | **Attribute levels** | **Minimum value** | **Midpoint value** | **Maximum value** |
| --- | --- | --- | --- | --- |
| Who the patient sees on the day of their check-up and how results are given to the patient | Optometrist or doctor, with results given in person to the patient on the day | n/a | n/a | n/a |
|  | Technician, with doctor reviewing results later and phoning the patient with results | n/a | n/a | n/a |
|  | Technician, with doctor reviewing results later and sending results to the patient by post | n/a | n/a | n/a |
| Appointment delay | No delay – appointments happen when they should | 0 | 0 | 0 |
|  | Up to 3 months | 1 | 2 | 3 |
|  | Between 4 months and 8 months | 4 | 6 | 8 |
|  | 9 months or more | 9 | 12 | 15 |
| Accessibility by public transport | Short journey with no difficult connections | n/a | n/a | n/a |
|  | Short journey with many or difficult connections | n/a | n/a | n/a |
|  | Long journey with no difficult connections | n/a | n/a | n/a |
|  | Long journey with many or difficult connections | n/a | n/a | n/a |
| Travel time | Less than 30 minutes | 10 | 20 | 30 |
|  | 30 minutes to 1 hour | 30 | 45 | 60 |
|  | More than 1 hour but less than 1 hour and 30 minutes | 60 | 75 | 90 |
|  | 1 hour and 30 minutes or more | 90 | 105 | 120 |
| Parking availability | No parking available | n/a | n/a | n/a |
|  | Parking available | n/a | n/a | n/a |
