# Supplementary material for: Preferences for community-based asynchronous-review eye clinics in the UK: A discrete choice experiment among patients, healthcare professionals, and the public
Source: PLoS One. 2026 Sep 15;21(9):e0342133. doi: 10.1371/journal.pone.0342133 (PMC13577492; doi:10.1371/journal.pone.0342133)
Supplement: Supplementary file 4 [file pone.0342133.s004.docx]

**Preferences for community-based asynchronous-review eye clinics in the UK: a discrete choice experiment among patients, healthcare professionals, and the public**

**Author information:**

Siyabonga Ndwandwe ^1*^, Angus IG Ramsay ^2^, Josefine Magnusson^2^, Steve Napier ^3‡^, Helen Baker ^3^, Jocelyn Cammack ^3^, Dun Jack Fu ^3^, Peng Tee Khaw ^3^, Sobha Sivaprasad ^3^, Hari Jayaram 3, Paul J Foster ^3^, Caroline S Clarke ^1^ on behalf of the HERCULES Consortium^

**^1^** Research Department of Primary Care and Population Health, University College London, London, United Kingdom

^2^ Research Department of Behavioural Science and Health, University College London, London, United Kingdom

^3^ National Institute for Health and Care Research Biomedical Research Centre at Moorfields Eye Hospital NHS Foundation Trust and UCL Institute of Ophthalmology, London, United Kingdom

^*^Corresponding author

e-mail: [s.ndwandwe@ucl.ac.uk](mailto:s.ndwandwe@ucl.ac.uk)

^‡^ Patient and public involvement representative

^ Membership of the HERCULES Consortium is provided in the Supporting Information.

# **S 4. Table. Within-group comparison tests**

| Subgroup comparisons | χ^2^ | DF |
| --- | --- | --- |
| In London vs Outside of London | 32.63*** | 8 |
| Female vs Male | 14.52* | 8 |
| Up to 65 years old vs 66 and older | 12.42 | 8 |
| Homeowner vs non-homeowner | 27.2*** | 8 |
| Full-time employment vs Retired vs Other employment | 18.42 | 16 |
| Full-time vs Retired | 16.61** | 8 |
| Retired vs Other employment | 6.13 | 8 |
| Full time employment vs Other employment | 3.59 | 8 |
| At least first-degree vs Non-degree holders | 16.85** | 8 |
| Public transport user vs Self-driver vs User of other modes of transport | 75.51*** | 16 |
| Public transport user vs Self-driver | 62.25*** | 8 |
| Self-driver vs Users of other modes of transport | 6.94 | 8 |
| Public transport user vs User of other modes of transport | 31.15*** | 8 |
| White vs Non-White | 11.78 | 8 |

DF = degrees of freedom, χ^2^ = Chi-squared statistic

* p<0.1, ** p<0.05, ***p<0.01
