# Supplementary material for: Preferences for community-based asynchronous-review eye clinics in the UK: A discrete choice experiment among patients, healthcare professionals, and the public
Source: PLoS One. 2026 Sep 15;21(9):e0342133. doi: 10.1371/journal.pone.0342133 (PMC13577492; doi:10.1371/journal.pone.0342133)
Supplement: Supplementary file 5 [file pone.0342133.s005.docx]

**Preferences for community-based asynchronous-review eye clinics in the UK: a discrete choice experiment among patients, healthcare professionals, and the public**

**Author information:**

Siyabonga Ndwandwe ^1*^, Angus IG Ramsay ^2^, Josefine Magnusson^2^, Steve Napier ^3‡^, Helen Baker ^3^, Jocelyn Cammack ^3^, Dun Jack Fu ^3^, Peng Tee Khaw ^3^, Sobha Sivaprasad ^3^, Hari Jayaram 3, Paul J Foster ^3^, Caroline S Clarke ^1^ on behalf of the HERCULES Consortium^

**^1^** Research Department of Primary Care and Population Health, University College London, London, United Kingdom

^2^ Research Department of Behavioural Science and Health, University College London, London, United Kingdom

^3^ National Institute for Health and Care Research Biomedical Research Centre at Moorfields Eye Hospital NHS Foundation Trust and UCL Institute of Ophthalmology, London, United Kingdom

^*^Corresponding author

e-mail: [s.ndwandwe@ucl.ac.uk](mailto:s.ndwandwe@ucl.ac.uk)

^‡^ Patient and public involvement representative

^ Membership of the HERCULES Consortium is provided in the Supporting Information.

# **S 5. Figure. Attribute ranking by type of respondent.**

S5 Fig A: Eye patients (n=141)

S5 Fig B. HCP (n=97)

S5 Fig C. Public (n=151)
