# Supplementary material for: Preferences for community-based asynchronous-review eye clinics in the UK: A discrete choice experiment among patients, healthcare professionals, and the public
Source: PLoS One. 2026 Sep 15;21(9):e0342133. doi: 10.1371/journal.pone.0342133 (PMC13577492; doi:10.1371/journal.pone.0342133)
Supplement: Supplementary file 6 [file pone.0342133.s006.docx]

**Preferences for community-based asynchronous-review eye clinics in the UK: a discrete choice experiment among patients, healthcare professionals, and the public**

**Author information:**

Siyabonga Ndwandwe ^1*^, Angus IG Ramsay ^2^, Josefine Magnusson^2^, Steve Napier ^3‡^, Helen Baker ^3^, Jocelyn Cammack ^3^, Dun Jack Fu ^3^, Peng Tee Khaw ^3^, Sobha Sivaprasad ^3^, Hari Jayaram 3, Paul J Foster ^3^, Caroline S Clarke ^1^ on behalf of the HERCULES Consortium^

**^1^** Research Department of Primary Care and Population Health, University College London, London, United Kingdom

^2^ Research Department of Behavioural Science and Health, University College London, London, United Kingdom

^3^ National Institute for Health and Care Research Biomedical Research Centre at Moorfields Eye Hospital NHS Foundation Trust and UCL Institute of Ophthalmology, London, United Kingdom

^*^Corresponding author

e-mail: [s.ndwandwe@ucl.ac.uk](mailto:s.ndwandwe@ucl.ac.uk)

^‡^ Patient and public involvement representative

^ Membership of the HERCULES Consortium is provided in the Supporting Information.

# **S6. Tables. Respondent attitudes towards ophthalmology services in the NHS**

S6 Table A. How long do you think it should take a typical patient to travel to their regular eye check- up appointment?

|  |  |  | Eye patients  n=141 | Healthcare professionals  n=97 | Public  n=151 | Total  n=389 |
| --- | --- | --- | --- | --- | --- | --- |
|  |  | 30 minutes | 52 | 54 | 102 | 208 |
|  |  | 60 minutes | 71 | 33 | 40 | 144 |
|  |  | 90 minutes | 8 | 3 | 5 | 16 |
|  |  | 120 minutes | 2 | 1 | 0 | 3 |
|  |  | Don’t know / Prefer not to say | 7 | 5 | 4 | 16 |
|  |  | Missing | 1 | 1 | 0 | 2 |
|  |  | Total | 140 | 96 | 151 | 389 |

S6 Table B. The quality of eye health services varies significantly across the different NHS regions.

|  |  | Eye patients  n=141 | Healthcare professionals  n=97 | Public  n=151 | Total  n=389 |
| --- | --- | --- | --- | --- | --- |
|  | Strongly disagree | 4 | 4 | 0 | 8 |
|  | Disagree | 7 | 9 | 8 | 24 |
|  | Neither agree nor disagree | 25 | 12 | 33 | 70 |
|  | Agree | 29 | 45 | 54 | 128 |
|  | Strongly agree | 25 | 24 | 12 | 61 |
|  | Don't know / Prefer not to say | 51 | 2 | 44 | 97 |
|  | Missing | 0 | 1 | 0 | 1 |
|  | Total | 141 | 97 | 151 | 389 |

S6 Table C. There is a relationship between eye health and overall health.

|  |  | Eye patients  n=141 | Healthcare professionals  n=97 | Public  n=151 | Total  n=389 |
| --- | --- | --- | --- | --- | --- |
|  | Strongly disagree | 5 | 4 | 1 | 10 |
|  | Disagree | 3 | 1 | 5 | 9 |
|  | Neither agree nor disagree | 15 | 3 | 12 | 30 |
|  | Agree | 59 | 41 | 73 | 173 |
|  | Strongly agree | 50 | 47 | 51 | 148 |
|  | Don't know / Prefer not to say | 9 | 0 | 7 | 16 |
|  | Missing | 0 | 1 | 2 | 3 |
|  | Total | 141 | 97 | 151 | 389 |

S6 Table D. I am satisfied with the skill and competency of the staff providing eye healthcare.

|  | Eye patients  n=141 | Healthcare professionals  n=97 | Public  n=151 | Total  n=389 |
| --- | --- | --- | --- | --- |
| Strongly disagree | 8 | 1 | 0 | 9 |
| Disagree | 10 | 3 | 1 | 14 |
| Neither agree nor disagree | 8 | 14 | 13 | 35 |
| Agree | 53 | 38 | 73 | 164 |
| Strongly agree | 60 | 34 | 47 | 141 |
| Don't know / Prefer not to say | 2 | 5 | 17 | 24 |
| Missing | 0 | 2 | 0 | 2 |
| Total | 141 | 97 | 151 | 389 |

S6 Table E. The staff providing eye healthcare are friendly and sensitive to cultural differences.

|  |  | Eye patients  n=141 | Healthcare professionals  n=97 | Public  n=151 | Total  n=389 |
| --- | --- | --- | --- | --- | --- |
|  | Strongly disagree | 6 | 1 | 2 | 9 |
|  | Disagree | 7 | 4 | 0 | 11 |
|  | Neither agree nor disagree | 21 | 20 | 29 | 70 |
|  | Agree | 55 | 39 | 48 | 142 |
|  | Strongly agree | 39 | 20 | 34 | 93 |
|  | Don't know / Prefer not to say | 13 | 13 | 38 | 64 |
|  | Missing | 0 | 0 | 0 | 0 |
|  | Total | 141 | 97 | 151 | 389 |

S6 Table F. I am open to having some appointments with my eye doctor over the phone or via video call instead of in person.

|  | Eye patients  n=141 | Healthcare professionals  n=97 | Public  n=151 | Total  n=389 |
| --- | --- | --- | --- | --- |
| Strongly disagree | 31 | 4 | 16 | 51 |
| Disagree | 32 | 21 | 47 | 100 |
| Neither agree nor disagree | 21 | 8 | 21 | 50 |
| Agree | 33 | 50 | 48 | 131 |
| Strongly agree | 21 | 11 | 16 | 48 |
| Don't know / Prefer not to say | 3 | 3 | 3 | 9 |
| Missing | 0 | 0 | 0 | 0 |
| Total | 141 | 97 | 151 | 389 |
