# Supplementary material for: Preferences for community-based asynchronous-review eye clinics in the UK: A discrete choice experiment among patients, healthcare professionals, and the public
Source: PLoS One. 2026 Sep 15;21(9):e0342133. doi: 10.1371/journal.pone.0342133 (PMC13577492; doi:10.1371/journal.pone.0342133)
Supplement: Supplementary file 7 [file pone.0342133.s007.docx]

**Preferences for community-based asynchronous-review eye clinics in the UK: a discrete choice experiment among patients, healthcare professionals, and the public**

**Author information:**

Siyabonga Ndwandwe ^1*^, Angus IG Ramsay ^2^, Josefine Magnusson^2^, Steve Napier ^3‡^, Helen Baker ^3^, Jocelyn Cammack ^3^, Dun Jack Fu ^3^, Peng Tee Khaw ^3^, Sobha Sivaprasad ^3^, Hari Jayaram 3, Paul J Foster ^3^, Caroline S Clarke ^1^ on behalf of the HERCULES Consortium^

**^1^** Research Department of Primary Care and Population Health, University College London, London, United Kingdom

^2^ Research Department of Behavioural Science and Health, University College London, London, United Kingdom

^3^ National Institute for Health and Care Research Biomedical Research Centre at Moorfields Eye Hospital NHS Foundation Trust and UCL Institute of Ophthalmology, London, United Kingdom

^*^Corresponding author

e-mail: [s.ndwandwe@ucl.ac.uk](mailto:s.ndwandwe@ucl.ac.uk)

^‡^ Patient and public involvement representative

^ Membership of the HERCULES Consortium is provided in the Supporting Information.

# **S 7. Table. Outputs of the conditional logit regression model**

Table S7a. Conditional logit regression model output

|  |  | **All Respondents**  **n=389** | **Eye Patients**  **n=141** | **HCP**  **n=97** | **Public**  **n=151** |
| --- | --- | --- | --- | --- | --- |
|  |  | B (SE) | B (SE) | B (SE) | B (SE) |
| Who the patient sees on the day of their check-up and how results are given to the patient | Optometrist or doctor, with results given in person to the patient on the day | *Reference category* | *Reference category* | *Reference category* | *Reference category* |
|  | Technician, with doctor reviewing results later and phoning the patient with results | -0.315*** (-0.06) | -0.529*** (-0.10) | -0.389** (-0.12) | -0.063 (-0.09) |
|  | Technician, with doctor reviewing results later and sending results to the patient by post | -0.582*** (-0.07) | -0.689*** (-0.12) | -0.766*** (-0.15) | -0.377*** (-0.10) |
| Accessibility by public transport | Long journey with many or difficult connections | *Reference category* | *Reference category* | *Reference category* | *Reference category* |
|  | Long journey with no difficult connections | 0.197** (-0.07) | 0.224 (-0.12) | 0.003 (-0.13) | 0.302** (-0.11) |
|  | Short journey with many or difficult connections | 0.363*** (-0.07) | 0.332** (-0.12) | 0.203 (-0.14) | 0.519*** (-0.12) |
|  | Short journey with no difficult connections | 0.513*** (-0.08) | 0.520*** (-0.13) | 0.399** (-0.13) | 0.597*** (-0.13) |
| Parking availability | Parking available | *Reference category* | *Reference category* | *Reference category* | *Reference category* |
|  | No parking available | -0.442*** (-0.05) | -0.178* (-0.08) | -0.550*** (-0.11) | -0.646*** (-0.09) |
| Delay days | Appointment delay (days) | -0.093*** (-0.01) | -0.095*** (-0.01) | -0.098*** (-0.02) | -0.093*** (-0.01) |
| Travel time | Travel time  (minutes) | -0.004*** (0.00) | -0.002 (0.00) | -0.005** (0.00) | -0.005*** (0.00) |
|  | Observations (choices) | 6224 | 2256 | 1552 | 2416 |
|  | BIC | 3853 | 1449 | 979 | 1495 |

BIC = Bayesian information criterion; β = estimated coefficient; SE = standard error; HCP = health care professionals; n = number of
