# Supplementary material for: Preferences for community-based asynchronous-review eye clinics in the UK: A discrete choice experiment among patients, healthcare professionals, and the public
Source: PLoS One. 2026 Sep 15;21(9):e0342133. doi: 10.1371/journal.pone.0342133 (PMC13577492; doi:10.1371/journal.pone.0342133)
Supplement: Supplementary file 8 [file pone.0342133.s008.docx]

**Preferences for community-based asynchronous-review eye clinics in the UK: a discrete choice experiment among patients, healthcare professionals, and the public**

**Author information:**

Siyabonga Ndwandwe ^1*^, Angus IG Ramsay ^2^, Josefine Magnusson^2^, Steve Napier ^3‡^, Helen Baker ^3^, Jocelyn Cammack ^3^, Dun Jack Fu ^3^, Peng Tee Khaw ^3^, Sobha Sivaprasad ^3^, Hari Jayaram 3, Paul J Foster ^3^, Caroline S Clarke ^1^ on behalf of the HERCULES Consortium^

**^1^** Research Department of Primary Care and Population Health, University College London, London, United Kingdom

^2^ Research Department of Behavioural Science and Health, University College London, London, United Kingdom

^3^ National Institute for Health and Care Research Biomedical Research Centre at Moorfields Eye Hospital NHS Foundation Trust and UCL Institute of Ophthalmology, London, United Kingdom

^*^Corresponding author

e-mail: [s.ndwandwe@ucl.ac.uk](mailto:s.ndwandwe@ucl.ac.uk)

^‡^ Patient and public involvement representative

^ Membership of the HERCULES Consortium is provided in the Supporting Information.

# **S 8. Table. Sensitivity of Willingness to wait to using minimum, mid, or maximum values appointment delays and travel time**

|  |  | **Willingness to wait -- all respondents (mean, 95% CI))** | | |
| --- | --- | --- | --- | --- |
|  |  | Min values | Mid values | Max values |
| Who the patient sees on the day of their check-up and how results are given to the patient | Optometrist or doctor, with results given in person on the day | Reference category | Reference category | Reference category |
|  | Technician, with doctor reviewing results later and phoning with results | -2.61 (-3.69, -1.53) | -3.39 (-4.78, -2.00) | -4.19 (-5.91, -2.48) |
|  | Technician, with doctor reviewing results later and sending results by post | -4.73 (-6.09, -3.37) | -6.27 (-8.03, -4.52) | -7.84 (-10.00, -5.67) |
| Accessibility by public transport | Long journey with many or difficult connections | Reference category | Reference category | Reference category |
|  | Long journey with no difficult connections | 1.64 (0.52, 2.75) | 2.12 (0.68, 3.57) | 2.61 (0.82, 4.40) |
|  | Short journey with many or difficult connections | 3.17 (1.93, 4.42) | 3.92 (2.30, 5.53) | 4.69 (2.68, 6.69) |
|  | Short journey with no difficult connections | 4.27 (2.94, 5.61) | 5.53 (3.80, 7.26) | 6.80 (4.66, 8.93) |
| Parking availability | Parking available | Reference category | Reference category | Reference category |
|  | No parking available | -3.68 (-4.67, -2.68) | -4.76 (-6.05, -3.48) | -5.87 (-7.45, -4.29) |
| Travel time | Travel time  (minutes) | -0.03 (-0.05, -0.02) | -0.04 (-0.06, -0.02) | -0.05 (-0.07, -0.03) |

CI = confidence interval
