# Supplementary material for: Preferences for community-based asynchronous-review eye clinics in the UK: A discrete choice experiment among patients, healthcare professionals, and the public
Source: PLoS One. 2026 Sep 15;21(9):e0342133. doi: 10.1371/journal.pone.0342133 (PMC13577492; doi:10.1371/journal.pone.0342133)
Supplement: Supplementary file 9 [file pone.0342133.s009.docx]

**Preferences for community-based asynchronous-review eye clinics in the UK: a discrete choice experiment among patients, healthcare professionals, and the public**

**Author information:**

Siyabonga Ndwandwe ^1*^, Angus IG Ramsay ^2^, Josefine Magnusson^2^, Steve Napier ^3‡^, Helen Baker ^3^, Jocelyn Cammack ^3^, Dun Jack Fu ^3^, Peng Tee Khaw ^3^, Sobha Sivaprasad ^3^, Hari Jayaram 3, Paul J Foster ^3^, Caroline S Clarke ^1^ on behalf of the HERCULES Consortium^

**^1^** Research Department of Primary Care and Population Health, University College London, London, United Kingdom

^2^ Research Department of Behavioural Science and Health, University College London, London, United Kingdom

^3^ National Institute for Health and Care Research Biomedical Research Centre at Moorfields Eye Hospital NHS Foundation Trust and UCL Institute of Ophthalmology, London, United Kingdom

^*^Corresponding author

e-mail: [s.ndwandwe@ucl.ac.uk](mailto:s.ndwandwe@ucl.ac.uk)

^‡^ Patient and public involvement representative

^ Membership of the HERCULES Consortium is provided in the Supporting Information.

# **S. 10. Table.** **Members of the HERCULES Research Consortium**

| First name | Last name | Affiliation |
| --- | --- | --- |
| Eleanor | Dean | Akeso |
| Olivia | Jeffrey | Akeso |
| Chris | Robson | Akeso |
| Nadine | Abdelgalil | Moorfields NHS FT |
| Joy | Adesanya | Moorfields NHS FT |
| Samiul | Alom | Moorfields NHS FT |
| Stacey | Angus | Moorfields NHS FT |
| Muna | Ayah | Moorfields NHS FT |
| Helen | Baker | Moorfields NHS FT |
| Connor | Beddow | Moorfields NHS FT |
| Paul | Cartwright | Moorfields NHS FT |
| Elisha | Chung | Moorfields NHS FT |
| George | Damianidis | Moorfields NHS FT |
| Sarah | Davies | Moorfields NHS FT |
| Sandi | Drewett | Moorfields NHS FT |
| Matala | Dyke | Moorfields NHS FT |
| Sherene | Ettiene | Moorfields NHS FT |
| Clare | Feasby | Moorfields NHS FT |
| Declan | Flanagan | Moorfields NHS FT |
| Dun Jack | Fu | Moorfields NHS FT |
| Gus | Gazzard | Moorfields NHS FT |
| Simranjit | Gill | Moorfields NHS FT |
| Steven | Gill | Moorfields NHS FT |
| Robin | Hamilton | Moorfields NHS FT |
| Nick | Hardie | Moorfields NHS FT |
| Jamie | Henderson | Moorfields NHS FT |
| Lesley | Henry | Moorfields NHS FT |
| Hari | Jayaram | Moorfields NHS FT |
| Aadil | Kazi | Moorfields NHS FT |
| Peng Tee | Khaw | Moorfields NHS FT |
| Chris | Leak | Moorfields NHS FT |
| Richard | Lee | Moorfields NHS FT |
| Sarah | Martin | Moorfields NHS FT |
| Mary | Masih | Moorfields NHS FT |
| Dhakshayini | Muhundhakumar | Moorfields NHS FT |
| Steve | Napier | Moorfields NHS FT |
| Luke | Nicholson | Moorfields NHS FT |
| Natalie | O'Shea | Moorfields NHS FT |
| Ella | Preston | Moorfields NHS FT |
| Kimberley | Quan | Moorfields NHS FT |
| Tulga | Reis | Moorfields NHS FT |
| Nick | Roberts | Moorfields NHS FT |
| Ana | Sanchez | Moorfields NHS FT |
| Kathryn | Scotcher | Moorfields NHS FT |
| Sobha | Sivaprasad | Moorfields NHS FT |
| Jon | Spencer | Moorfields NHS FT |
| Peter | Thomas | Moorfields NHS FT |
| Rachel | Thompson | Moorfields NHS FT |
| Karen | Titmus | Moorfields NHS FT |
| Jonathan | Wilson | Moorfields NHS FT |
| Nick | Hynes | Somo Global |
| Tom | Blair | Ubisense |
| Paul | Webster | Ubisense |
| Omar | Abolnaga | UCL |
| Farbod | Afshar Bakeshloo | UCL |
| Kenan | Arifoglu | UCL |
| Nick | Burt | UCL |
| Caroline | Clarke | UCL |
| Dolores | Conroy | UCL |
| Ian | Eames | UCL |
| Holly | Elphinstone | UCL |
| Ecem | Ergin | UCL |
| Iqbal | Fahmi | UCL |
| Paul | Foster | UCL |
| Susana | Frazao Pinheiro | UCL |
| Naomi | Fulop | UCL |
| Saheli | Gandhi | UCL |
| Steven | Gray | UCL |
| Xiaojia | Guo | UCL |
| Rouba | Ibrahim | UCL |
| Hanna | James | UCL |
| Melih | Kamaoglu | UCL |
| Stephanie | Kumpunen | UCL |
| Xiaoming | Li | UCL |
| Josefine | Magnusson | UCL |
| Dominika | Matusiak | UCL |
| Grant | Mills | UCL |
| Siyabonga | Ndwandwe | UCL |
| Pei Li | Ng | UCL |
| Giovanni | Ometto | UCL |
| Rosica | Pachilova | UCL |
| Angus | Ramsay | UCL |
| Yaman | Rawas Kalaji | UCL |
| Daniel | Rennie | UCL |
| Irinie | Roufaeel | UCL |
| Kerstin | Sailer | UCL |
| Mine | Sak Acur | UCL |
| Peter | Scully | UCL |
| Lina | Song | UCL |
| Anne | Symons | UCL |
| Yue | Tang | UCL |
| Ahmed | Tarek Zaky Fouad | UCL |
| Melike | Toprak | UCL |
| Jemima | Unwin Teji | UCL |
| Martin | Utley | UCL |
| Duncan | Wilson | UCL |
| Dongyuan | Zhan | UCL |
| Jocelyn | Cammack | UCL and Moorfields NHS FT |
| Paula | Lorgelly | University of Auckland |
