# Supplementary material for: Preferences for community-based asynchronous-review eye clinics in the UK: A discrete choice experiment among patients, healthcare professionals, and the public
Source: PLoS One. 2026 Sep 15;21(9):e0342133. doi: 10.1371/journal.pone.0342133 (PMC13577492; doi:10.1371/journal.pone.0342133)
Supplement: Supplementary file 11 [file pone.0342133.s011.docx]

**Preferences for community-based asynchronous-review eye clinics in the UK: a discrete choice experiment among patients, healthcare professionals, and the public**

**Author information:**

Siyabonga Ndwandwe ^1*^, Angus IG Ramsay ^2^, Josefine Magnusson^2^, Steve Napier ^3‡^, Helen Baker ^3^, Jocelyn Cammack ^3^, Dun Jack Fu ^3^, Peng Tee Khaw ^3^, Sobha Sivaprasad ^3^, Hari Jayaram 3, Paul J Foster ^3^, Caroline S Clarke ^1^ on behalf of the HERCULES Consortium^

**^1^** Research Department of Primary Care and Population Health, University College London, London, United Kingdom

^2^ Research Department of Behavioural Science and Health, University College London, London, United Kingdom

^3^ National Institute for Health and Care Research Biomedical Research Centre at Moorfields Eye Hospital NHS Foundation Trust and UCL Institute of Ophthalmology, London, United Kingdom

^*^Corresponding author

e-mail: [s.ndwandwe@ucl.ac.uk](mailto:s.ndwandwe@ucl.ac.uk)

^‡^ Patient and public involvement representative

^ Membership of the HERCULES Consortium is provided in the Supporting Information.

**S 11. Tables. Additional Health Status Questions**

A. Health conditions amongst respondents living with a health condition (n= 185, 48%)

| Health condition | Eye patients | Healthcare professionals | General Public | Total |
| --- | --- | --- | --- | --- |
| Long-term eye condition | 37 (36%) | 1 (4%) | 6 (11%) | 44 (24%) |
| Non-long-term eye condition | 5 (5%) | 3 (13%) | 5 (9%) | 13 (7%) |
| Diabetes | 11 (11%) | 2 (8%) | 4 (7%) | 17 (9%) |
| Hypertension | 8 (8%) | 1 (4%) | 2 (4%) | 11 (6%) |
| Other long-term conditions | 32 (31%) | 13 (54%) | 17 (30%) | 62 (34%) |
| Other non-long-term conditions | 6 (6%) | 1 (4%) | 4 (7%) | 11 (6%) |
| Prefer not to say | 0 (0%) | 2 (8%) | 5 (9%) | 7 (4%) |
| Missing | 5 (5%) | 1 (4%) | 14 (25%) | 20 (11%) |

B. Eye check-up frequency amongst respondents living with longer-term or short-term eye conditions

| Recommended routine eye health check-up frequency | Eye patients | Healthcare professionals | General Public | Total |
| --- | --- | --- | --- | --- |
| Once every 2-6 months | 21 (50%) | 0 (0%) | 0 (0%) | 21 (37%) |
| Once every 7-12 months | 16 (38%) | 1 (25%) | 5 (45%) | 22 (39%) |
| Once every 13-18 months | 2 (5%) | 0 (0%) | 2 (18%) | 4 (7%) |
| Once every longer than 18 months | 2 (5%) | 0 (0%) | 3 (27%) | 5 (9%) |
| Prefer not to say | 0 (0%) | 2 (50%) | 1 (9%) | 3 (5%) |
| Missing | 1 (2%) | 1 (25%) | 0 (0%) | 2 (4%) |

C. Use of regular eye injections amongst respondents living with longer-term or short-term eye conditions

| Regular use of eye injections | Eye patients | Healthcare professionals | General Public | Total |
| --- | --- | --- | --- | --- |
| Yes | 12 (29%) | 0 (0%) | 2 (18%) | 14 (25%) |
| No | 30 (71%) | 2 (50%) | 9 (82%) | 41 (72%) |
| Prefer not to say | 0 (0%) | 1 (25%) | 0 (0%) | 1 (2%) |
| Missing | 0 (0%) | 1 (25%) | 0 (0%) | 1 (2%) |
