# Supplementary material for: Preferences for community-based asynchronous-review eye clinics in the UK: A discrete choice experiment among patients, healthcare professionals, and the public
Source: PLoS One. 2026 Sep 15;21(9):e0342133. doi: 10.1371/journal.pone.0342133 (PMC13577492; doi:10.1371/journal.pone.0342133)
Supplement: Supplementary file 12 [file pone.0342133.s013.docx]

**Reponses to editorial and reviewer comments**

**Title:** Preferences for community-based asynchronous-review eye clinics in the UK: a discrete choice experiment among patients, healthcare professionals, and the public

**Author information:**

Siyabonga Ndwandwe ^1*^, Angus IG Ramsay ^2^, Josefine Magnusson^2^, Steve Napier ^3‡^, Helen Baker ^3^, Jocelyn Cammack ^3^, Dun Jack Fu ^3^, Peng Tee Khaw ^3^, Sobha Sivaprasad ^3^, Hari Jayaram ^3^, Paul J Foster ^3^, Caroline S Clarke ^1^

|  | Comments | Responses |
| --- | --- | --- |
| 1 | Please ensure that your manuscript meets PLOS ONE's style requirements, including those for file naming. The PLOS ONE style templates can be found at  https://journals.plos.org/plosone/s/file?id=wjVg/PLOSOne_formatting_sample_main_body.pdf and  https://journals.plos.org/plosone/s/file?id=ba62/PLOSOne_formatting_sample_title_authors_affiliations.pdf | Thank you for sharing the links to PLOS ONE style templates. We have taken note and revised our manuscript according to the outlined PLOS ONE style and conventions. |
| 2 | Thank you for stating in your Funding Statement:  This research was supported by the National Institute for Health and Care Research (NIHR) Moorfields Biomedical Research Centre (BRC), which is a partnership between Moorfields Eye Hospital NHS Foundation Trust and UCL Institute of Ophthalmology, and the Moorfields Eye Hospital Research and Development Department. The views expressed are those of the authors and not necessarily of the NIHR or the Department of Health and Social Care or other funders. Commercial partners for the wider HERCULES project were Zeiss, Optos, and Ubisense. The funder had no role in the design and conduct of the study; collection, management, analysis, and interpretation of the data; preparation, review, or approval of the manuscript; or decision to submit the manuscript for publication.    Please provide an amended statement that declares *all* the funding or sources of support (whether external or internal to your organization) received during this study, as detailed online in our guide for authors at http://journals.plos.org/plosone/s/submit-now.  Please also include the statement “There was no additional external funding received for this study.” in your updated Funding Statement.  Please include your amended Funding Statement within your cover letter. We will change the online submission form on your behalf. | Thank you for your comment. We have revised the cover letter to include a comprehensive funding statement, disclosing all sources of support for the manuscript. We have also clarified that the funders had no role in the study design, data collection and analysis, decision to publish, or manuscript preparation. Additionally, we have stated that no external funding was received beyond the sources already declared. |
| 3 | Thank you for stating the following in the Acknowledgments Section of your manuscript:  The authors of this acknowledge the contributions of all members of the HERCULES Research Consortium. A full list of the members of the HERCULES Research Consortium can be found in the supplementary materials part 10.    We note that you have provided funding information that is not currently declared in your Funding Statement. However, funding information should not appear in the Acknowledgments section or other areas of your manuscript. We will only publish funding information present in the Funding Statement section of the online submission form.  Please remove any funding-related text from the manuscript and let us know how you would like to update your Funding Statement. Currently, your Funding Statement reads as follows:  This research was supported by the National Institute for Health and Care Research (NIHR) Moorfields Biomedical Research Centre (BRC), which is a partnership between Moorfields Eye Hospital NHS Foundation Trust and UCL Institute of Ophthalmology, and the Moorfields Eye Hospital Research and Development Department. The views expressed are those of the authors and not necessarily of the NIHR or the Department of Health and Social Care or other funders. Commercial partners for the wider HERCULES project were Zeiss, Optos, and Ubisense. The funder had no role in the design and conduct of the study; collection, management, analysis, and interpretation of the data; preparation, review, or approval of the manuscript; or decision to submit the manuscript for publication.    Please include your amended statements within your cover letter; we will change the online submission form on your behalf. | Thank you for your comment. We have now removed the funding statement from the manuscript, in line with the journal’s requirements. Thank you for offering to change the online submission form on our behalf, we updated the cover letter accordingly. |
| 4 | Thank you for stating the following in the Competing Interests section:  Siyabonga Ndwandwe and Caroline Clarke have honorary research associate contracts with Moorfields Eye Hospital NHS Foundation Trust (required for receiving data for another aspect of the HERCULES project). Dun Jack Fu has an academic clinical research contract with Moorfields Eye Hospital NHS Foundation Trust. Sobha Sivaprasad and Hari Jayaram are employed by, while Sir Peng T Khaw and Paul J Foster have honorary contracts with, Moorfields Eye Hospital NHS Foundation Trust. Sir Peng T Khaw is Co-Director of, and Helen Baker and Jocelyn Cammack are staff at, the National Institute for Health and Care Research (NIHR) Biomedical Research Centre (BRC) at Moorfields Eye Hospital NHS Foundation Trust and UCL Institute of Ophthalmology. Sir Peng Khaw is also supported by the Helen Hamlyn Trust, the Nolan Family, the Katz Foundation, Moorfields Eye.  Steve Napier is a volunteer at the National Institute for Health and Care Research (NIHR) Biomedical Research Centre (BRC) at Moorfields Eye Hospital NHS Foundation Trust and UCL Institute of Ophthalmology. Angus IG Ramsay and Josefine Magnusson reports no competing interests.    We note that one or more of the authors are employed by a commercial company.  a. Please provide an amended Funding Statement declaring this commercial affiliation, as well as a statement regarding the Role of Funders in your study. If the funding organization did not play a role in the study design, data collection and analysis, decision to publish, or preparation of the manuscript and only provided financial support in the form of authors' salaries and/or research materials, please review your statements relating to the author contributions, and ensure you have specifically and accurately indicated the role(s) that these authors had in your study. You can update author roles in the Author Contributions section of the online submission form.    Please also include the following statement within your amended Funding Statement.  “The funder provided support in the form of salaries for authors [insert relevant initials], but did not have any additional role in the study design, data collection and analysis, decision to publish, or preparation of the manuscript. The specific roles of these authors are articulated in the ‘author contributions’ section.”  If your commercial affiliation did play a role in your study, please state and explain this role within your updated Funding Statement.    b. Please also provide an updated Competing Interests Statement declaring this commercial affiliation along with any other relevant declarations relating to employment, consultancy, patents, products in development, or marketed products, etc.    Within your Competing Interests Statement, please confirm that this commercial affiliation does not alter your adherence to all PLOS ONE policies on sharing data and materials by including the following statement: "This does not alter our adherence to  PLOS ONE policies on sharing data and materials.” (as detailed online in our guide for authors http://journals.plos.org/plosone/s/competing-interests) . If this adherence statement is not accurate and  there are restrictions on sharing of data and/or materials, please state these. Please note that we cannot proceed with consideration of your article until this information has been declared.    Please include both an updated Funding Statement and Competing Interests Statement in your cover letter. We will change the online submission form on your behalf. | Thank you very much for this helpful comment. We have updated both the *Competing Interests* and *Funding* sections to reflect the role of funders in our study and to declare relevant commercial affiliations of co-authors. Specifically:   1. We have clarified that the funder had no role in the study design, data collection and analysis, decision to publish, or preparation of the manuscript. 2. We have disclosed that, at the time of drafting this manuscript, Steve Napier (our Patient and Public Involvement Engagement [PPIE] representative) was affiliated with Janes, a global open-source intelligence company specialising in military, national security, aerospace, and transport. This affiliation did not influence any aspect of the study. All PPIE activities were conducted independently and transparently, with appropriate safeguards to ensure the integrity of public involvement. This does not affect our adherence to PLOS ONE’s policies on data and materials sharing. 3. We have revised the co-authors’ competing interests to include any additional fees or honoraria received outside the submitted work. |
| 5 | We note that you have indicated that there are restrictions to data sharing for this study. For studies involving human research participant data or other sensitive data, we encourage authors to share de-identified or anonymized data. However, when data cannot be publicly shared for ethical reasons, we allow authors to make their data sets available upon request. For information on unacceptable data access restrictions, please see http://journals.plos.org/plosone/s/data-availability#loc-unacceptable-data-access-restrictions.    Before we proceed with your manuscript, please address the following prompts:    a) If there are ethical or legal restrictions on sharing a de-identified data set, please explain them in detail (e.g., data contain potentially identifying or sensitive patient information, data are owned by a third-party organization, etc.) and who has imposed them (e.g., a Research Ethics Committee or Institutional Review Board, etc.). Please also provide contact information for a data access committee, ethics committee, or other institutional body to which data requests may be sent.    b) If there are no restrictions, please upload the minimal anonymized data set necessary to replicate your study findings to a stable, public repository and provide us with the relevant URLs, DOIs, or accession numbers. Please see http://www.bmj.com/content/340/bmj.c181.long for guidelines on how to de-identify and prepare clinical data for publication. For a list of recommended repositories, please see https://journals.plos.org/plosone/s/recommended-repositories. You also have the option of uploading the data as Supporting Information files, but we would recommend depositing data directly to a data repository if possible.    Please update your Data Availability statement in the submission form accordingly. | Thank you for this clarifying comment. We have updated the *Data Availability* section accordingly. We have noted that the data are not publicly available due to ethical and data governance restrictions imposed by the North East–York Research Ethics Committee and University College London. The dataset contains sensitive patient information, and consent for sharing beyond the study team was not obtained. However, requests for collaborative access to anonymised data will be considered upon reasonable request to the corresponding author, in line with the data sharing policies of the study sponsor. |
| 6 | Your ethics statement should only appear in the Methods section of your manuscript. If your ethics statement is written in any section besides the Methods, please delete it from any other section. | Thank you for pointing out this oversight. We have moved the ethics statement into the methods section. It is now the last paragraph in the methods section. |
| 7 | We note that this data set consists of interview transcripts. Can you please confirm that all participants gave consent for interview transcript to be published?  If they DID provide consent for these transcripts to be published, please also confirm that the transcripts do not contain any potentially identifying information (or let us know if the participants consented to having their personal details published and made publicly available). We consider the following details to be identifying information:  - Names, nicknames, and initials  - Age more specific than round numbers  - GPS coordinates, physical addresses, IP addresses, email addresses  - Information in small sample sizes (e.g. 40 students from X class in X year at X university)  - Specific dates (e.g. visit dates, interview dates)  - ID numbers    Or, if the participants DID NOT provide consent for these transcripts to be published:  - Provide a de-identified version of the data or excerpts of interview responses  - Provide information regarding how these transcripts can be accessed by researchers who meet the criteria for access to confidential data, including:  a) the grounds for restriction  b) the name of the ethics committee, Institutional Review Board, or third-party organization that is imposing sharing restrictions on the data  c) a non-author, institutional point of contact that is able to field data access queries, in the interest of maintaining long-term data accessibility.  d) Any relevant data set names, URLs, DOIs, etc. that an independent researcher would need in order to request your minimal data set.    For further information on sharing data that contains sensitive participant information, please see: https://journals.plos.org/plosone/s/data-availability#loc-human-research-participant-data-and-other-sensitive-data    If there are ethical, legal, or third-party restrictions upon your dataset, you must provide all of the following details (https://journals.plos.org/plosone/s/data-availability#loc-acceptable-data-access-restrictions):  a) A complete description of the dataset  b) The nature of the restrictions upon the data (ethical, legal, or owned by a third party) and the reasoning behind them  c) The full name of the body imposing the restrictions upon your dataset (ethics committee, institution, data access committee, etc)  d) If the data are owned by a third party, confirmation of whether the authors received any special privileges in accessing the data that other researchers would not have  e) Direct, non-author contact information (preferably email) for the body imposing the restrictions upon the data, to which data access requests can be sent | Thank you very much for this comment. This manuscript is based on written online survey data and did not involve conducting any interviews and as such there are no transcripts. As noted in the *Methods* section (under *Experimental Design*) and detailed in *S1. The full survey for the Discrete Choice Experiment*, we administered an online survey at the national level in the UK. Participants were informed that by completing and submitting the survey, they were providing consent to participate in the study. Additionally, before proceeding with the survey, respondents were required to access a webpage containing the full Participant Information Sheet that outlined the purpose of the study, what participation would involve, and how their responses would be processed.  We have added in a few words to clarify this on line 153 in the manuscript. |
| 8 | Please include captions for your Supporting Information files at the end of your manuscript, and update any in-text citations to match accordingly. Please see our Supporting Information guidelines for more information: http://journals.plos.org/plosone/s/supporting-information. | Thank you for this comment. We have now included captions for all supplementary materials and reorganised the original document into ten distinct components, each prepared as an individual file. This restructuring aims to improve clarity and accessibility for readers. |
| 9 | If the reviewer comments include a recommendation to cite specific previously published works, please review and evaluate these publications to determine whether they are relevant and should be cited. There is no requirement to cite these works unless the editor has indicated otherwise. | Thank you for this comment. None of the reviewers have requested us to cite specific works in the literature |
|  | **Reviewer 1 comments (review points to consider)** |  |
| 10 | The abstract presents clear picture of what readers should expect with the whole work and shows consistency throughout the write-up while also filling a research gap in patient care in eye clinics. The research appears to be well thought-out with rigorous design demonstrated, consistent referencing and supplementary materials provided. Sentences are precise and easy to understand despite presenting complex scenarios. The results were clearly presented and shows analytical rigour with the discussion section well-presented and highlighting related studies. | We would like to thank the reviewer for their positive and generous comments regarding our manuscript and the design of our study. We greatly appreciate their thoughtful feedback and are pleased that the study’s approach and contribution were well received. |
| 11 | First, perhaps for non-expert readers, the paper could benefit from a few lines in the introductory section that provides more details of cases where asynchronous-review eye clinics have been successful in other countries if there are no sufficient evidence from the UK. | We thank the reviewer for this suggestion. We have looked at the earlier iterations of asynchronous eye clinics and in light of this comment have added a few lines highlighting the use of this service model in diabetic retinopathy screening in Sweden, Singapore, and the UK. We state that:  “Asynchronous-review clinics are not a new innovation in ophthalmology. Earlier iterations of the model include mobile diabetic retinopathy screening programmes, as introduced in Stockholm, Sweden, in 1990 and in Singapore in 2010 [7]. The UK has one of the largest and most successful diabetic retinopathy screening programmes, where trained technicians capture 45° colour fundus photographs that are later reviewed by clinicians; an approach that contributed to diabetic retinopathy no longer being the leading cause of blindness registration in the UK by 2010, for the first time in 50 years [7].” |
| 12 | Second review point is with regards to this statement “All respondents were asked to respond from a patient’s perspective, so that the same language could be used for all, to make the results comparable across respondent types and simplify distribution of the survey link”. While asking all respondents to respond from a patient’s perspective as stated above is justified by the authors in the statement, it is difficult to be certain that respondents that do not fall into the patient category can make choices as patient respondents would since they may not have the experiences in terms of treatment and appointment needs as patients would. Also, even if non-patient respondents responded from patients’ perspective, it could imply that the preferences of non-patient respondents themselves were not really captured for comparisons with the responses of patients. Thus, querying why there was a need to involve non-patient respondents or whether the opinions of non-patient respondents were actually captured by the survey. | Thank you very much for this clarifying comment.  Firstly, inviting participants to respond from a patient’s perspective enabled us to explore what key stakeholders perceive as fair and valuable to patients.  Secondly, as shown in Tables 4 and 5, as well as Supplementary tables S5 and S7, we conducted extensive subgroup analyses involving eye patients, healthcare professionals (HCPs), and members of the public as part of our sensitivity analyses. For example, we found that the public was statistically indifferent between seeing an optometrist or doctor and receiving same-day results (reference category) versus seeing a technician and receiving results by phone. In contrast, eye patients and HCPs exhibited statistically significant preferences (p < 0.001), with the lowest utility associated with seeing a technician and receiving results by post (S7 Table. Outputs of the conditional logit regression model).  We also report that patients required a reduction of 7.29 months (95% CI: 4.25 to 10.32) to accept seeing a technician and receiving results by post, compared to the reference category. This figure was 7.84 months for HCPs and 4.07 months for the public. Additionally, patients required a reduction in travel time of 305 minutes to accept this trade-off, which was approximately four times greater than the public’s requirement of 73 minutes (Table 5).  Overall, we believe this approach has allowed us to assess what key stakeholders—whether current eye patients, potential future patients, or policy constituents—consider fair to patients. We believe that recognising differences in what patients value versus what stakeholders assume patients value is a key and crucial step in designing truly patient-centred care. To this end, We have also thoroughly examined the heterogeneity of preferences across these three stakeholder groups. |
|  | **Reviewer 2 comments** |  |
| 13 | This research explores alternative treatment options for patients with eye diseases with the involvement of technicians. While this research is promising, it must be cautious because there are many types of eye diseases ranging from mild to severe, and not all can be treated by technicians. The questions in this study are not about general eye treatments, nor are they differentiated or grouped by type of disease. If the eye disease groups were differentiated, the service options would be different. | We would like to thank the reviewer for this insightful comment and agree that “there are many types of eye diseases ranging from mild to severe, and not all can be treated by technicians.”  Our study focuses on services for patients with stable chronic eye conditions that require regular monitoring and pose low clinical risk. This was made explicit in the survey design. For example, in Section Two of the questionnaire, participants were asked to consider “features of services where patients with a stable long-term eye condition might go for their regular eye check-ups” (*S 1. The full survey for the Discrete Choice Experiment*).  We intentionally kept the condition description broad to allow for generalisability to other high-volume, low-complexity specialties within the National Health Service (NHS) that also require routine monitoring. The wider HERCULES Project, within which this study is situated, principally focuses on glaucoma and medical retina conditions—both of which account for a substantial proportion of outpatient ophthalmology appointments in NHS England |
| 14 | The respondents were divided into three groups: patients, healthcare professionals, and the public. The last two groups could not indicate whether or not they had ever experienced eye pain (this question was not included in the questionnaire). Respondents who have experienced eye pain may differ from those who have not in their choice of services. | We would like to thank the reviewer for this insightful comment. Indeed, respondents who have experienced eye pain may differ from those who have not in their choice of services. We have now noted this concern as a fourth limitation in our study. We state that, “… there potentially could be underlying heterogeneity in each respondent group that could shape preferences. For example, preferences for non-eye-patient respondents who have experienced eye pain might differ from those who have not. However, this is secondary and would require a very large sample to sufficiently tease out.” |
| 15 | The concept of willingness to pay is examined in relation to willingness to wait and willingness to travel. Is there data on how respondents' occupations relate to time expenditure? Those with time-consuming jobs tend to choose short-distance services. | We thank the reviewer for this insightful comment. In Section 4, Question 10, of our questionnaire, we asked respondents to report their employment status (*S 1. The full survey for the Discrete Choice Experiment*). As detailed in *S 4. Table. Within-group comparison tests*, our within-group comparison tests found that that preferences were statistically indifferent between those in full-time employment vs retired vs other employment, retired vs other employment, full time employment vs other employment. Statistically significant different preferences were detected between those in full-time employment vs retired. We do not have any further breakdown on the types of jobs that people had.  In response to this comment, we have now added summary data on occupational status to Table 3 to enhance clarity and transparency. |
| 16 | If no information is available from the previous point, it can be included as a limitation of this research | This information has been captured in *S 4. Table. Within-group comparison tests*. |
