# Supplementary material for: Preferences for community-based asynchronous-review eye clinics in the UK: A discrete choice experiment among patients, healthcare professionals, and the public
Source: PLoS One. 2026 Sep 15;21(9):e0342133. doi: 10.1371/journal.pone.0342133 (PMC13577492; doi:10.1371/journal.pone.0342133)
Supplement: Supplementary file 13 [file pone.0342133.s014.docx]

**Responses to peer reviewer comments, April 2026**

**Title:** Preferences for community-based asynchronous-review eye clinics in the UK: a discrete choice experiment among patients, healthcare professionals, and the public

**Author information:**

Siyabonga Ndwandwe ^1*^, Angus IG Ramsay ^2^, Josefine Magnusson^2^, Steve Napier ^3‡^, Helen Baker ^3^, Jocelyn Cammack ^3^, Dun Jack Fu ^3^, Peng Tee Khaw ^3^, Sobha Sivaprasad ^3^, Hari Jayaram ^3^, Paul J Foster ^3^, Caroline S Clarke ^1^

| **Reviewer** | **Reviewers’ comments** | **Authors’ responses** |
| --- | --- | --- |
| Reviewer 1 | The authors have addressed all my comments in the first review and have made changes in the article to reflect them | No further response required |
| Reviewer 2 | (No Response) | No further response required |
| Reviewer 3: Methods Clarification | Line 175-176: Please clarify the eligibility criteria used in Section 1 to screen respondents. | We thank the reviewer for this helpful comment. We have now clarified the eligibility criteria (lines 177-178). The manuscript now also states:  “To be eligible, participants had to be at least 18 years old.” |
| Reviewer 3: Methods Clarification | Consistency Checks: You note the absence of consistency/dominance checks as a limitation. Consider briefly discussing in the methods or limitations why this common DCE practice was omitted (e.g., due to cognitive burden concerns from your design work). | We thank the reviewer for raising this important point and we have added some discussion on this in the Limitations section (lines 394-402). |
| Reviewer 3: Results Presentation | Figure 1 & Legend: The legend for Figure 1 (lines 278-282) describes results for a different topic ("acceptable mode of appointment") which seems misplaced. The legend should describe the attribute ranking figure itself. The text currently in the legend appears to belong in the Supplementary Materials or a different part of the results (e.g., around Supplementary Part 6). | We thank the reviewer for identifying this discrepancy. The legend for Figure 1 has now been corrected to accurately describe the attribute ranking figure.  The revised legend is:  Figure 1 Legend:  “A simple ranking of the five attributes showed that 44% (173/389) felt that the composite attribute describing who patient sees on the day and mode of results communication was the most important and 44% (173/389) felt that parking availability was the least important of the five.” |
| Reviewer 3: Results Presentation | Tables 4 & 5: The estimates for Patients in Tables 4 and 5 have very wide confidence intervals (e.g., WTT: -305 (-628, 18) minutes). This should be briefly acknowledged in the text as indicating less precise estimates within this subgroup, potentially due to heterogeneity or sample size. | We thank the reviewer for this observation. The wide willingness‑to‑travel (WTT) confidence intervals for the patient subgroup arise because, as shown in supplementary table, S7 Table, travel time was not a statistically significant predictor of utility for this group. We have now explicitly acknowledged this in the Results section (lines 311-314), noting that the imprecision likely reflects a weaker association between travel time and preferences in patients. |
| Reviewer 3: Discussion Refinement | Limitations - Socioeconomic Representativeness: The point about homeownership rates is well-taken. Consider also commenting briefly on the overrepresentation of highly educated respondents (60% with a degree) and its potential impact on the generalizability of preferences. | We thank the reviewer for this comment. We have now added a comment on this in the limitations section (lines 408-410). |
| Reviewer 3: Discussion Refinement | Interpretation of WTW: The discussion on lines 357-370 about patients "willing to wait... longer to see a clinician" is insightful. To avoid misinterpretation, you could explicitly state that from a service design perspective, the primary utility of the finding is in quantifying the reduction in delay needed to make technician-led models acceptable, not in endorsing longer delays. | We thank the reviewer for this helpful comment. We are indeed cautious to avoid any possible misinterpretation, and we have added this further clarification in lines 415-421. |
| Reviewer 3:  Language and Copyediting | Please perform a thorough proofread to correct the typographical errors, awkward line breaks, and minor grammatical issues noted above and throughout the text. Ensure consistency in table formatting | We thank the reviewer for this comment. We have revised the work and corrected these issues throughout. |
| Reviewer 4 | The exploration of alternative service delivery models in the interest of public health is commendable, especially given the premise that delayed care can equate to denied care. The study is well-designed to assess the trade-offs patients are willing to make, such as balancing travel time against waiting periods for appointments. It is particularly insightful in evaluating patient preferences in the context of technician-led clinics, remote result communication, and reduced appointment delays. Understanding these preferences is crucial for shaping more efficient and accessible healthcare services. However, there are notable limitations regarding the sample composition. A significant proportion of respondents—approximately 79% of patients and 65% of the general public—were above 55 years of age. This age distribution likely influenced the findings on willingness to wait and to travel, as older individuals may have different preferences and constraints compared to younger populations. Furthermore, the majority of participants were from outside London, where access to healthcare facilities and public transport may differ from larger cities. This could explain the greater acceptance of local technician-led models. If the sample had included a broader age range and more participants from metropolitan areas with robust public transport infrastructure, the acceptance of non-clinician-led care might have yielded different results. Future studies should consider a more heterogeneous sample to enhance the generalisability of the findings. In public health service delivery there is no one shoe that fits all as shown in literature so what works in once place and population group | We thank the reviewer for these thoughtful observations. |
| Reviewer 4 | Recommendation: Since the study clearly indicated that clinician driven care was the preferred option and sample was skewed more geriatric, it would be good to conclude saying that technician led care of service delivery could be considered as a door step/ close to home care model in remote locations for geriatric population as part of NHS service. And considering that same day results was also a preferred option, this could be coupled with AI or telehealth or MHealth platforms. for expert opinion on the spot. | We thank the reviewer for these thoughtful comments.  On lines 346-352, at the end of the Results section, we described aggregating some variables to attempt to assess some level of heterogeneity across potential subgroups. For example, we collapsed the eight age groups highlighted in table 3 into two groups: respondents aged ≤65 years and those ≥66 years and then compared responses between these two groups, as shown in Table S4, but found no statistically significant difference in direction or magnitude between the two groups.  We did not collect information that would allow us to state the relative degree of remoteness of residence. The closest we have for this is collapsing place-of-residence subgroups to London and Out-of-London, which we had also done and which is described similarly in lines 346-349. As Table S4 shows, we found that preferences in these two subgroups had the same direction but statistically different magnitude.  We feel that the suggestions made by the reviewer might not be sufficiently evidenced by the analysis and dataset that we have, so we have not added any further comments on this to the manuscript.  The reviewer further suggested that we could consider aligning the results with topical issues like AI and MHealth platforms. We greatly appreciate this consideration; however, as we did not consider such development in the study design we have similarly decided not to add in comments on this. If the Editor feels strongly that we should make some comment along these lines, then we would be happy to discuss this further with them. |
| Reviewer 5 - overview | In “Preferences for community-based asynchronous-review eye clinics in the UK: a discrete choice experiment among patients, healthcare professionals, and the public,” Siyabonga Ndwandwe et al. examine the trade-offs that eye-care users are willing to make between care setting and time costs when accessing care for stable eye conditions. The study demonstrates that decentralizing chronic eye-care monitoring from hospitals to community-based settings can help reduce pressure on hospital clinics, among other benefits highlighted in the paper. A particular strength is the stratification of respondents by their interaction with eye-care and healthcare services, which allows the authors to focus on individuals with actual eye-care needs who are most likely to use community-based services.  The authors present a clear research question and apply a robust methodology aligned with their aims. The results are clearly reported and thoughtfully discussed. Overall, the manuscript is well written and makes for an engaging and informative read. | We thank the reviewer for these kind comments. |

| Reviewer 5 – specific comments | 1. The severity of patients' eye conditions likely influences the urgency of their eye care needs and thus their willingness to wait for care or travel to receive it. The authors collected information on the frequency of eye follow-up visits and the receipt of regular intraocular injections. Could the authors clarify how these factors were incorporated into the analysis?  As mentioned in lines 243–244, were similar tests conducted within patient subgroups stratified by frequency of eye care use? | We thank the reviewer for this insightful comment. Our study was however not designed to assess within‑group heterogeneity by disease severity or frequency of eye care use. Such analyses would have required substantially larger subgroups, with sufficient balance. For example, table 3 shows that 82% (n=318) of all respondents’ self-reported health status was good (n=244, 63%) or very good (n=74, 19%), while only 16% (n= 62) of all respondents reported poor or very poor health status. Conducting a within-group analysis with this level of sample imbalance would have yielded imprecise estimates, potentially with very wide confidence intervals. In some instances, within-group sample imbalance would have limited convergence of the conditional logit model: for example, Table S11 shows that 100% of the 21 respondents living with long-term and short-term eye conditions were patients, therefore we could not go into this level of detail in the analysis by type of respondent.  We have clarified the mention of this limitation in the Limitations section and we hope that it reads more clearly. |
| --- | --- | --- |
|  | The discussion section is brief and does not address all the presented results. A focused discussion of the implications of the subgroup analyses (lines 326–332) would provide valuable insight and strengthen the paper. | We thank the reviewer for this valuable suggestion. However, we intentionally avoided over‑interpreting results due to sample size constraints—the study was not powered to detect within-group differences beyond the three broad type of respondents. We have added a comment in the discussion to state that the small sample size precludes further discussion here on these points. |
