# Supplementary material for: Preferences for community-based asynchronous-review eye clinics in the UK: A discrete choice experiment among patients, healthcare professionals, and the public
Source: PLoS One. 2026 Sep 15;21(9):e0342133. doi: 10.1371/journal.pone.0342133 (PMC13577492; doi:10.1371/journal.pone.0342133)
Supplement: Supplementary file 14 [file pone.0342133.s015.docx]

**Responses to peer reviewer comments, July 2026**

**Title:** Preferences for community-based asynchronous-review eye clinics in the UK: a discrete choice experiment among patients, healthcare professionals, and the public

**Author information:**

Siyabonga Ndwandwe ^1^, Angus IG Ramsay ^2^, Josefine Magnusson^2^, Steve Napier ^3^, Helen Baker ^3^, Jocelyn Cammack ^3^, Dun Jack Fu ^3^, Peng Tee Khaw ^3^, Sobha Sivaprasad ^3^, Hari Jayaram ^3^, Paul J Foster ^3^, Caroline S Clarke ^1^

^1^Research Department of Primary Care and Population Health, University College London, London, United Kingdom

^2^ Research Department of Behavioural Science and Health, University College London, London, United Kingdom

^3^ National Institute for Health and Care Research Biomedical Research Centre at Moorfields Eye Hospital NHS Foundation Trust and UCL Institute of Ophthalmology, London, United Kingdom

| **Reviewers’ comments** | **Authors’ responses** |
| --- | --- |
| Reviewer #1: All comments have been addressed | No further response required |
| Reviewer #3: (No Response) | No further response required |
| Reviewer #4: All comments have been addressed | No further response required |
| Reviewer #5: All comments have been addressed | No further response required |
| 2. Is the manuscript technically sound, and do the data support the conclusions? |  |
| The manuscript must describe a technically sound piece of scientific research with data that supports the conclusions. Experiments must have been conducted rigorously, with appropriate controls, replication, and sample sizes. The conclusions must be drawn appropriately based on the data presented. |  |
| Reviewer #1: Yes | No further response required |
| Reviewer #3: Yes | No further response required |
| Reviewer #4: Yes | No further response required |
| Reviewer #5: Yes | No further response required |
| 3. Has the statistical analysis been performed appropriately and rigorously? |  |
| Reviewer #1: Yes | No further response required |
| Reviewer #3: Yes | No further response required |
| Reviewer #4: Yes | No further response required |
| Reviewer #5: Yes | No further response required |
| 4. Have the authors made all data underlying the findings in their manuscript fully available? |  |
| [The PLOS Data policy requires authors to make all data underlying the findings described in their manuscript fully available without restriction, with rare exception (please refer to the Data Availability Statement in the manuscript PDF file). The data should be provided as part of the manuscript or its supporting information, or deposited to a public repository. For example, in addition to summary statistics, the data points behind means, medians and variance measures should be available. If there are restrictions on publicly sharing data—e.g. participant privacy or use of data from a third party—those must be specified.](https://eur01.safelinks.protection.outlook.com/?url=http%3A%2F%2Ftrack.editorialmanager.com%2FCL0%2Fhttp%3A%252F%252Fwww.plosone.org%252Fstatic%252Fpolicies.action%2523sharing%2F1%2F010f019f0178b4e7-040d43ed-d86f-4f90-a72f-b1e46b867ca2-000000%2FYwbmkYFS6EBtcOgPuSPGYrF7w9TCshlzHvxcF5QUQ50%3D258&data=05%7C02%7Cs.ndwandwe%40ucl.ac.uk%7Cb9e534ebac894365555e08ded31f73a0%7C1faf88fea9984c5b93c9210a11d9a5c2%7C0%7C0%7C639180329206599516%7CUnknown%7CTWFpbGZsb3d8eyJFbXB0eU1hcGkiOnRydWUsIlYiOiIwLjAuMDAwMCIsIlAiOiJXaW4zMiIsIkFOIjoiTWFpbCIsIldUIjoyfQ%3D%3D%7C0%7C%7C%7C&sdata=QYCiC9L6r2jZJhmMcjk%2FAYuz1JfBUVG9%2FSauP3%2FU40E%3D&reserved=0) |  |
| Reviewer #1: Yes | No further response required |
| Reviewer #3: No | We thank the reviewer for noting this. We have provided an accompanying data availability statement as part of additional information on the submission portal, where we state that:  “Due to the UK’s national legislative framework (UK GDPR and UK Data Protection Act 2018), institutional data governance arrangements (Moorfields Eye Hospital NHS Foundation Trust and University College London) and ethical considerations (North East - York Research Ethics Committee; Reference 21/NE/0164), the datasets generated and/or analysed during the study cannot be made universally publicly available. Some components of the dataset contain confidential patient information. However, bona fide requests for collaborative access to anonymised data will be considered on a case-by-case basis upon reasonable request to the corresponding author and in accordance with standard data sharing regulations and policies of the study sponsor. Initial enquiries should be directed to the Research Governance Lead, Moorfields Eye Hospital NHS Foundation Trust ([moorfields.resadmin@nhs.net](mailto:moorfields.resadmin@nhs.net)).  Some of our data is available and has been provided via supplementary information files, titled 'Supplementary materials.zip” |
| Reviewer #4: Yes | No further response required |
| Reviewer #5: (No Response) | No further response required |
| 5. Is the manuscript presented in an intelligible fashion and written in standard English? |  |
| PLOS ONE does not copyedit accepted manuscripts, so the language in submitted articles must be clear, correct, and unambiguous. Any typographical or grammatical errors should be corrected at revision, so please note any specific errors here. |  |
| Reviewer #1: Yes | No further response required |
| Reviewer #3: Yes | No further response required |
| Reviewer #4: Yes | No further response required |
| Reviewer #5: Yes | No further response required |
| 6. Review Comments to the Author |  |
| Please use the space provided to explain your answers to the questions above. You may also include additional comments for the author, including concerns about dual publication, research ethics, or publication ethics. (Please upload your review as an attachment if it exceeds 20,000 characters) |  |
| Reviewer #1: (No Response) | No further response required |
| Reviewer #3: Major Strengths |  |
| >Unique contribution: This is the first DCE to explicitly test preference for a technician-led asynchonous-review model of ophthalmology care, filling in a critical knowledge gap for implementation science of workforce-efficient clinical models.  >Sound methodology: The D-optimal design, forced-choice, and choice between dominance checks (justified for reasons of cognitive load) are thoughtful choices. >Relevant clinical insights: The core finding that respondents would accept technician-led care if appointment wait times were cut by 3-6 months (or time travelling to the appointment by 1-2.5 hours) offers useful insights for service planners. | We thank the reviewer for their positive and encouraging assessment of our study. We appreciate the recognition of the study's novelty, methodological rigour, and practical relevance to health planning and policy. |
| Major Concerns to Address |  |
| 1. Generalisability |  |
| >The authors note some substantial demographic bias (79% homeowners vs. national average of 62.5%; 60% college degree vs. national average of 33%), but this should be emphasised in the abstract and conclusions. These factors are known to affect preferences for health-care services and may affect generalizability to socioeconomically more diverse communities. The authors should consider: >Including a statement of limitations in the abstract >Including more detail on how these may impact the WTW/WTT estimates (e.g., higher education is associated with stronger preferences for more contact with health care providers? | We thank the reviewer for this comment. In response, we have added a statement in the abstract acknowledging this limitation and expanded the discussion in the limitations section. Specifically, in lines 425 - 428, we note that socioeconomic factors and other unobserved respondent characteristics may influence preferences for healthcare delivery models. This would consequently affect the estimated willingness-to-wait (WTW) and willingness-to-travel (WTT) values. However, the available sample size did not support sufficiently powered subgroup analyses to examine these effects formally. As a result, we are unable to isolate the influence these factors might have on preferences and WWT/WTT estimates. Future research using a larger, more socioeconomically diverse sample would be valuable for better understanding how patient characteristics shape preferences for asynchronous, technician-led ophthalmology care. |
| 2. Inconsistent patient vs. public perspective |  |
| All respondents (including HCPs and the public) were asked to answer "from a patient's perspective". This approach is problematic for the following reasons: >HCPs may find it challenging to put aside their own beliefs and thoughts, creating a response bias >The members of the public without chronic eye disease lack the experience needed to develop preferences >These are two different groups (subgroup analyses) but still asked to take on a patient perspective Suggestion: This should be explained and defended, or the authors should report it as a weakness. Perhaps the use of independent models (patient vs. reference) could be justified, rather than having them all think in a certain way. | We thank the reviewer for this important observation. Our objective was to understand what different stakeholder groups, including healthcare professionals (HCPs) and members of the public, consider acceptable and appropriate for patients, given their important role in the implementation and support of new care models.  In response, we have justified the selected approach as both methodological and practical. In lines 213 – 220, we state that:  “Using a single patient-focused questionnaire across all respondent groups ensured consistency in attribute framing, wording, and choice tasks, reducing the risk that differences in responses would be driven by variations in survey design rather than underlying preferences. It also enabled direct comparison of how different stakeholder groups perceived patient preferences within a common decision-making framework; understanding the gap between what patients value and what stakeholders assume patients value is a key and crucial step in designing truly patient-centred care.” |
| 3. Unrealistic WTT estimates range beyond attributes |  |
| The authors acknowledge the estimated WTTs (e.g., 305 minutes for patients who would accept technician+post vs. reference) are larger than the largest travel times presented in the DCE (90+ minutes). They refer to other data, but this implies extrapolations. The authors should: >Clearly report these estimates are extrapolations and should be used carefully >Explore the possibility of a non-linear utility function in modelling travel time values >Include the proportion of respondents who had WTT values higher than the highest attribute level | We thank the reviewer for this important observation. Firstly, we agree that our WTT estimates amongst patients extend beyond the range of travel times presented in the DCE; therefore, they should be interpreted with caution. This might be due to the wide WTT confidence intervals “because travel time did not significantly influence their preferences (see S7 file)”, (lines 352 -354). We further note in the discussion (lines 438 - 440) that this result is consistent with other findings in the literature (Lu et al., 2019).    Secondly, we want to note that WTT estimates are derived from average marginal rates of substitution; therefore, it is not possible, given the statistical software used, to determine the proportion of respondents with values exceeding the highest attribute level presented in the experiment.  Thirdly, we thank the reviewer for suggesting that we explore non-linear specifications of travel time. However, our study design was not sufficient to robustly estimate and compare more complex functional forms. This would be a welcome consideration in future studies.  Lastly, we thank the reviewer for suggesting that we include the proportion of respondents who had WTT values higher than the highest attribute level. However, the WTT estimates are derived from average marginal rates of substitution, and it is not possible, given the statistical software used, to determine the proportion of respondents with values exceeding the highest attribute level presented in the experiment. |
| 4. Handling of missing data and completion rate |  |
| The authors say "rates of missing data were very low" but this is not quantified. An average of 14.5 minutes (SD 26.3) to complete had a long tail, with some taking a long time. The authors should: >Include the survey completion rate (percentage of the survey started completed) >Report the number of people who initiated but did not complete the survey >Disclose rates of missing data for key variables | We thank the reviewer for this comment and suggestions. All variables with missing values are reported in Table 3 and also in S6 Table, which reports respondents' attitudes. To improve clarity, we have revised the methods section to contextualise missingness. We state in lines 183 - 184 that sections 1 to 3 were required to be completed before progressing through the survey. This ensured that there was no missingness on key components of the survey.  On the other hand, the survey was administered and hosted by a third-party survey company, as described in the methods section (lines 168 – 169). Consequently, we do not have information on the number or characteristics of individuals who may have started but not completed the survey, as only completed responses were provided to the research team. We have now clarified this in the methods section, in lines 202 - 207. Therefore, we do not have the information on non-completers to carry out the reviewer’s suggestions. |
| 5. Composite attribute interpretability |  |
| The composite attribute ("who the patient sees") and ("how the results are given") is justified but causes interpretational problems. The bundle "clinician + same-day results" is different to the other bundles (technician + phone or technician + post) in two ways. This means the WTW estimates bundle: >The disutility of technician (not clinician) >The disutility of phone/post vs. in-person communication >The disutility of phone/post vs. face-to-face >The authors note this but consider whether changing the design (e.g., separate attributes with interactions) may have allowed for decomposition of this. | We thank the reviewer for this insightful comment. The composite attribute was intentionally designed to reflect the real-world service delivery model, where the type of healthcare professional and method of communicating results are experienced jointly by patients. Our objective was therefore to value these service configurations as they occur in practice, rather than to decompose the separate effects of clinician type and result communication method. We have clarified this rationale in lines 151-154 of the manuscript. |
| Minor Concerns and Clarifications Needed |  |
| 6. Table size: Table 3 is long. Move data to the supplement or reduce categories. | We thank the reviewer for this suggestion. However, we have retained the full table because all categories are relevant to interpreting the study findings and provide a complete picture of the sample, including the extent and pattern of missing data. Reducing categories or moving these data to the supplementary material may limit transparency and make it more difficult for readers to fully assess the study population. We would, however, be happy to move this table to the supplementary information if the editor feels that would be preferable. |
| 7. Interpretation of confidence intervals: For patients WTT estimates (Table 5), 95% CI for technician+post (-628 to 18) includes zero and is very large, corresponding to the non-significant travel time coefficient in S7. This is noted, but should be made clearer that they are statistically unreliable and should not be misinterpreted. | We thank the reviewer for this helpful observation. We agree that the WTT estimate for the technician + post scenario should be interpreted with caution. As the reviewer has noted, we have stated that the 95% confidence interval is wide and includes zero, reflecting considerable uncertainty arising from the non-significant travel time coefficient. We have clarified this in the results (lines 352 - 354) and discussion sections (lines 434 - 452) |
| 8. Opportunity for more with attitudinal data: Section 4 involved attitudinal data collection but not in the main findings. Could any insights be added from the attitudinal data (e.g. relationship between trust in technicians and preferences). | We thank the reviewer for this comment. As highlighted in the previous round of reviews, our study was not designed to assess within‑group heterogeneity by disease severity or frequency of eye care use. Such analyses would have required substantially larger subgroups, with sufficient balance. Conducting a within-group analysis with this level of sample imbalance would have yielded imprecise estimates, potentially with very wide confidence intervals. In some instances, within-group sample imbalance would have limited convergence of the conditional logit model. In lines 434 - 440 in the discussion section, we state that this level of detailed assessment, beyond the exploratory analyses presented in the Supplementary Materials, would require a larger sample size. |
| 9. Conflicts of interest: Multiple authors (PJF, SS, PTK, HJ) have multiple industry ties. These are all declared but readers should remain alert to the influence of sponsorship (this is less likely in a DCE). | We thank the reviewer for this comment. We assure the reviewer and PLOS ONE readership that all relevant competing interests and industry affiliations have been fully disclosed in accordance with journal requirements to ensure transparency. We have further clarified in the competing interests section (lines 496 - 498) that the views expressed in this manuscript are those of the authors and do not necessarily reflect those of their employers, affiliated institutions, funding bodies, or industry collaborators. |
| Questions for Authors |  |
| >Did you investigate preference heterogeneity (mixed logit and latent class)? If yes, please provide estimates. If not, explain why on not needed conditional logit. | We thank the reviewer for this comment. In response, we have revised the methods section to clarify this point; in lines 249– 255, we now state that:  “We used conditional logit regression in Stata 17 [16] to assess the strength of stated preferences from section 3. As the analysis was designed to compare preferences across the three predefined respondent groups (eye patients, HCP, and the public), rather than to investigate preference heterogeneity beyond these groups, conditional logit models were considered appropriate. Separate models were specified for each respondent group, allowing comparison of the direction and magnitude of preferences across groups.” |
| >How did you check that the composite attribute was understood? Were checks made using the longer descriptions? | We thank the reviewer for this comment. The survey materials underwent several rounds of review and refinement with key stakeholders, including patient and public involvement and engagement (PPIE) representatives. This process helped ensure that the survey content, wording, and instructions were clear, appropriate, and written in plain English for the target audience. We were unfortunately not able to ascertain directly for each respondent that they had understood this, as the survey was completed online. We have revised the limitations section (line 421 - 424) of the manuscript and hope that this is now clearer. |
| >Since 49% of patients used public transport to attend appointments, but only 19% of the public would be willing to do so, does this indicate hypothetical bias in the public's responses from adopting a patient perspective? | We thank the reviewer for this insightful comment. While there are differences in characteristics between patients and members of the public, we do not believe that these differences alone provide conclusive evidence of hypothetical bias that would prevent the public from adopting a patient perspective. Or a difference in preference not captured by the analysis in some way, albeit exploratory. For example, in addition to the use of different modes of transportation between patients and the public, Table 3 in the manuscript shows that a greater proportion of patients in our sample were based in London than members of the public. The subgroup analysis presented in S4 Table, suggests that preferences vary by geographic location. Whilst this statistic captures broad differences by location, it does not directly assess differences between London and non-London respondents within each respondent group. |
| >The fact that schedules are delayed implicitly and by implication patients would wait 6 months for appointments (lines 378-380) appears to imply a change in direction of causality for the WTW estimate. Please note that the estimate is the compensation needed for patients to accept care from a technician, not a preference for a longer wait. | We thank the reviewer for highlighting this point. Our intended interpretation is centred on the trade-off between provider type and waiting time, with the WTW estimate reflecting the additional waiting time patients would be willing to accept as compensation for receiving care from a technician. We have revised the manuscript to reiterate this key implication of the results in lines 394 to 400. |
| Recommendation |  |
| Minor Revision - The manuscript is well done and a valuable contribution but needs to address the need for representativeness, for providing care from a patient's perspective, and explain the WTT concerns. | We thank the reviewer for their positive assessment of our manuscript and for the constructive suggestions. |
| Reviewer #4: The authors have tried to incorporate the comments with the available data. And I have no further comments for them. |  |
| Reviewer #5: (No Response) |  |
