# Supplementary material for: Preferences for community-based asynchronous-review eye clinics in the UK: A discrete choice experiment among patients, healthcare professionals, and the public
Source: PLoS One. 2026 Sep 15;21(9):e0342133. doi: 10.1371/journal.pone.0342133 (PMC13577492; doi:10.1371/journal.pone.0342133)
Supplement: Peer review history

# Peer review: Decision Letter 0

**DOI:** 10.1371/journal.pone.0342133.r001

**Authors:** Davide Costa

15 Jul 2025

Dear Dr. Ndwandwe,

Thank you for submitting your manuscript to PLOS ONE. After careful consideration, we feel that it has merit but does not fully meet PLOS ONE’s publication criteria as it currently stands. Therefore, we invite you to submit a revised version of the manuscript that addresses the points raised during the review process.

**The manuscript is interesting but some reviewers' concerns should be addressed before we can reconsider your paper for publication.**

Please submit your revised manuscript by Aug 29 2025 11:59PM. If you will need more time than this to complete your revisions, please reply to this message or contact the journal office at plosone@plos.org. When you're ready to submit your revision, log on to https://www.editorialmanager.com/pone/ and select the 'Submissions Needing Revision' folder to locate your manuscript file.

- A rebuttal letter that responds to each point raised by the academic editor and reviewer(s). You should upload this letter as a separate file labeled 'Response to Reviewers'.
- A marked-up copy of your manuscript that highlights changes made to the original version. You should upload this as a separate file labeled 'Revised Manuscript with Track Changes'.
- An unmarked version of your revised paper without tracked changes. You should upload this as a separate file labeled 'Manuscript'.

If you would like to make changes to your financial disclosure, please include your updated statement in your cover letter. Guidelines for resubmitting your figure files are available below the reviewer comments at the end of this letter.

If applicable, we recommend that you deposit your laboratory protocols in protocols.io to enhance the reproducibility of your results. Protocols.io assigns your protocol its own identifier (DOI) so that it can be cited independently in the future. For instructions see: https://journals.plos.org/plosone/s/submission-guidelines#loc-laboratory-protocols. Additionally, PLOS ONE offers an option for publishing peer-reviewed Lab Protocol articles, which describe protocols hosted on protocols.io. Read more information on sharing protocols at https://plos.org/protocols?utm_medium=editorial-email&utm_source=authorletters&utm_campaign=protocols.

We look forward to receiving your revised manuscript.

Kind regards,

Davide Costa

Academic Editor

PLOS ONE

**Journal Requirements:**

1. When submitting your revision, we need you to address these additional requirements.

Please ensure that your manuscript meets PLOS ONE's style requirements, including those for file naming. The PLOS ONE style templates can be found at

https://journals.plos.org/plosone/s/file?id=wjVg/PLOSOne_formatting_sample_main_body.pdf and

https://journals.plos.org/plosone/s/file?id=ba62/PLOSOne_formatting_sample_title_authors_affiliations.pdf

2. Thank you for stating in your Funding Statement:

This research was supported by the National Institute for Health and Care Research (NIHR) Moorfields Biomedical Research Centre (BRC), which is a partnership between Moorfields Eye Hospital NHS Foundation Trust and UCL Institute of Ophthalmology, and the Moorfields Eye Hospital Research and Development Department. The views expressed are those of the authors and not necessarily of the NIHR or the Department of Health and Social Care or other funders. Commercial partners for the wider HERCULES project were Zeiss, Optos, and Ubisense. The funder had no role in the design and conduct of the study; collection, management, analysis, and interpretation of the data; preparation, review, or approval of the manuscript; or decision to submit the manuscript for publication.

Please provide an amended statement that declares *all* the funding or sources of support (whether external or internal to your organization) received during this study, as detailed online in our guide for authors at http://journals.plos.org/plosone/s/submit-now.  Please also include the statement “There was no additional external funding received for this study.” in your updated Funding Statement.

Please include your amended Funding Statement within your cover letter. We will change the online submission form on your behalf.

3. Thank you for stating the following in the Acknowledgments Section of your manuscript:

The authors of this acknowledge the contributions of all members of the HERCULES Research Consortium. A full list of the members of the HERCULES Research Consortium can be found in the supplementary materials part 10.

We note that you have provided funding information that is not currently declared in your Funding Statement. However, funding information should not appear in the Acknowledgments section or other areas of your manuscript. We will only publish funding information present in the Funding Statement section of the online submission form.

Please remove any funding-related text from the manuscript and let us know how you would like to update your Funding Statement. Currently, your Funding Statement reads as follows:

This research was supported by the National Institute for Health and Care Research (NIHR) Moorfields Biomedical Research Centre (BRC), which is a partnership between Moorfields Eye Hospital NHS Foundation Trust and UCL Institute of Ophthalmology, and the Moorfields Eye Hospital Research and Development Department. The views expressed are those of the authors and not necessarily of the NIHR or the Department of Health and Social Care or other funders. Commercial partners for the wider HERCULES project were Zeiss, Optos, and Ubisense. The funder had no role in the design and conduct of the study; collection, management, analysis, and interpretation of the data; preparation, review, or approval of the manuscript; or decision to submit the manuscript for publication.

Please include your amended statements within your cover letter; we will change the online submission form on your behalf.

4. Thank you for stating the following in the Competing Interests section:

Siyabonga Ndwandwe and Caroline Clarke have honorary research associate contracts with Moorfields Eye Hospital NHS Foundation Trust (required for receiving data for another aspect of the HERCULES project). Dun Jack Fu has an academic clinical research contract with Moorfields Eye Hospital NHS Foundation Trust. Sobha Sivaprasad and Hari Jayaram are employed by, while Sir Peng T Khaw and Paul J Foster have honorary contracts with, Moorfields Eye Hospital NHS Foundation Trust. Sir Peng T Khaw is Co-Director of, and Helen Baker and Jocelyn Cammack are staff at, the National Institute for Health and Care Research (NIHR) Biomedical Research Centre (BRC) at Moorfields Eye Hospital NHS Foundation Trust and UCL Institute of Ophthalmology. Sir Peng Khaw is also supported by the Helen Hamlyn Trust, the Nolan Family, the Katz Foundation, Moorfields Eye.

Steve Napier is a volunteer at the National Institute for Health and Care Research (NIHR) Biomedical Research Centre (BRC) at Moorfields Eye Hospital NHS Foundation Trust and UCL Institute of Ophthalmology. Angus IG Ramsay and Josefine Magnusson reports no competing interests.

We note that one or more of the authors are employed by a commercial company.

a. Please provide an amended Funding Statement declaring this commercial affiliation, as well as a statement regarding the Role of Funders in your study. If the funding organization did not play a role in the study design, data collection and analysis, decision to publish, or preparation of the manuscript and only provided financial support in the form of authors' salaries and/or research materials, please review your statements relating to the author contributions, and ensure you have specifically and accurately indicated the role(s) that these authors had in your study. You can update author roles in the Author Contributions section of the online submission form.

Please also include the following statement within your amended Funding Statement.

“The funder provided support in the form of salaries for authors [insert relevant initials], but did not have any additional role in the study design, data collection and analysis, decision to publish, or preparation of the manuscript. The specific roles of these authors are articulated in the ‘author contributions’ section.”

If your commercial affiliation did play a role in your study, please state and explain this role within your updated Funding Statement.

b. Please also provide an updated Competing Interests Statement declaring this commercial affiliation along with any other relevant declarations relating to employment, consultancy, patents, products in development, or marketed products, etc.

Within your Competing Interests Statement, please confirm that this commercial affiliation does not alter your adherence to all PLOS ONE policies on sharing data and materials by including the following statement: "This does not alter our adherence to  PLOS ONE policies on sharing data and materials.” (as detailed online in our guide for authors http://journals.plos.org/plosone/s/competing-interests) . If this adherence statement is not accurate and  there are restrictions on sharing of data and/or materials, please state these. Please note that we cannot proceed with consideration of your article until this information has been declared.

Please include both an updated Funding Statement and Competing Interests Statement in your cover letter. We will change the online submission form on your behalf.

5. We note that you have indicated that there are restrictions to data sharing for this study. For studies involving human research participant data or other sensitive data, we encourage authors to share de-identified or anonymized data. However, when data cannot be publicly shared for ethical reasons, we allow authors to make their data sets available upon request. For information on unacceptable data access restrictions, please see http://journals.plos.org/plosone/s/data-availability#loc-unacceptable-data-access-restrictions.

Before we proceed with your manuscript, please address the following prompts:

a) If there are ethical or legal restrictions on sharing a de-identified data set, please explain them in detail (e.g., data contain potentially identifying or sensitive patient information, data are owned by a third-party organization, etc.) and who has imposed them (e.g., a Research Ethics Committee or Institutional Review Board, etc.). Please also provide contact information for a data access committee, ethics committee, or other institutional body to which data requests may be sent.

b) If there are no restrictions, please upload the minimal anonymized data set necessary to replicate your study findings to a stable, public repository and provide us with the relevant URLs, DOIs, or accession numbers. Please see http://www.bmj.com/content/340/bmj.c181.long for guidelines on how to de-identify and prepare clinical data for publication. For a list of recommended repositories, please see https://journals.plos.org/plosone/s/recommended-repositories. You also have the option of uploading the data as Supporting Information files, but we would recommend depositing data directly to a data repository if possible.

Please update your Data Availability statement in the submission form accordingly.

6. Your ethics statement should only appear in the Methods section of your manuscript. If your ethics statement is written in any section besides the Methods, please delete it from any other section.

7. We note that this data set consists of interview transcripts. Can you please confirm that all participants gave consent for interview transcript to be published?

If they DID provide consent for these transcripts to be published, please also confirm that the transcripts do not contain any potentially identifying information (or let us know if the participants consented to having their personal details published and made publicly available). We consider the following details to be identifying information:

- Names, nicknames, and initials

- Age more specific than round numbers

- GPS coordinates, physical addresses, IP addresses, email addresses

- Information in small sample sizes (e.g. 40 students from X class in X year at X university)

- Specific dates (e.g. visit dates, interview dates)

- ID numbers

Or, if the participants DID NOT provide consent for these transcripts to be published:

- Provide a de-identified version of the data or excerpts of interview responses

- Provide information regarding how these transcripts can be accessed by researchers who meet the criteria for access to confidential data, including:

a) the grounds for restriction

b) the name of the ethics committee, Institutional Review Board, or third-party organization that is imposing sharing restrictions on the data

c) a non-author, institutional point of contact that is able to field data access queries, in the interest of maintaining long-term data accessibility.

d) Any relevant data set names, URLs, DOIs, etc. that an independent researcher would need in order to request your minimal data set.

For further information on sharing data that contains sensitive participant information, please see: https://journals.plos.org/plosone/s/data-availability#loc-human-research-participant-data-and-other-sensitive-data

If there are ethical, legal, or third-party restrictions upon your dataset, you must provide all of the following details (https://journals.plos.org/plosone/s/data-availability#loc-acceptable-data-access-restrictions):

a) A complete description of the dataset

b) The nature of the restrictions upon the data (ethical, legal, or owned by a third party) and the reasoning behind them

c) The full name of the body imposing the restrictions upon your dataset (ethics committee, institution, data access committee, etc)

d) If the data are owned by a third party, confirmation of whether the authors received any special privileges in accessing the data that other researchers would not have

e) Direct, non-author contact information (preferably email) for the body imposing the restrictions upon the data, to which data access requests can be sent

8. Please include captions for your Supporting Information files at the end of your manuscript, and update any in-text citations to match accordingly. Please see our Supporting Information guidelines for more information: http://journals.plos.org/plosone/s/supporting-information.

9. If the reviewer comments include a recommendation to cite specific previously published works, please review and evaluate these publications to determine whether they are relevant and should be cited. There is no requirement to cite these works unless the editor has indicated otherwise.

[Note: HTML markup is below. Please do not edit.]

Reviewers' comments:

Reviewer's Responses to Questions

**Comments to the Author**

1. Is the manuscript technically sound, and do the data support the conclusions?

Reviewer #1: Yes

Reviewer #2: Partly

**********

2. Has the statistical analysis been performed appropriately and rigorously?

Reviewer #1: Yes

Reviewer #2: Yes

**********

3. Have the authors made all data underlying the findings in their manuscript fully available?

The PLOS Data policy

Reviewer #1: No

Reviewer #2: No

**********

4. Is the manuscript presented in an intelligible fashion and written in standard English?

Reviewer #1: Yes

Reviewer #2: Yes

**********

**Reviewer #1:**  Review for Preferences for community-based asynchronous-review eye clinics in the UK: a discrete choice experiment among patients, healthcare professionals, and the public.

The abstract presents clear picture of what readers should expect with the whole work and shows consistency throughout the write-up while also filling a research gap in patient care in eye clinics.

The research appears to be well thought-out with rigorous design demonstrated, consistent referencing and supplementary materials provided.

Sentences are precise and easy to understand despite presenting complex scenarios. The results were clearly presented and shows analytical rigour with the discussion section well-presented and highlighting related studies.

Two review points to consider:

First, perhaps for non-expert readers, the paper could benefit from a few lines in the introductory section that provides more details of cases where asynchronous-review eye clinics have been successful in other countries if there are no sufficient evidence from the UK.

Second review point is with regards to this statement “All respondents were asked to respond from a patient’s perspective, so that the same language could be used for all, to make the results comparable across respondent types and simplify distribution of the survey link”. While asking all respondents to respond from a patient’s perspective as stated above is justified by the authors in the statement, it is difficult to be certain that respondents that do not fall into the patient category can make choices as patient respondents would since they may not have the experiences in terms of treatment and appointment needs as patients would. Also, even if non-patient respondents responded from patients’ perspective, it could imply that the preferences of non-patient respondents themselves were not really captured for comparisons with the responses of patients. Thus, querying why there was a need to involve non-patient respondents or whether the opinions of non-patient respondents were actually captured by the survey.

**Reviewer #2:** 1.This research explores alternative treatment options for patients with eye diseases with the involvement of technicians. While this research is promising, it must be cautious because there are many types of eye diseases ranging from mild to severe, and not all can be treated by technicians. The questions in this study are not about general eye treatments, nor are they differentiated or grouped by type of disease. If the eye disease groups were differentiated, the service options would be different.

2.The respondents were divided into three groups: patients, healthcare professionals, and the public. The last two groups could not indicate whether or not they had ever experienced eye pain (this question was not included in the questionnaire). Respondents who have experienced eye pain may differ from those who have not in their choice of services.

3.The concept of willingness to pay is examined in relation to willingness to wait and willingness to travel. Is there data on how respondents' occupations relate to time expenditure? Those with time-consuming jobs tend to choose short-distance services.

4.If no information is available from the previous point, it can be included as a limitation of this research

**********

what does this mean?). If published, this will include your full peer review and any attached files.

If you choose “no”, your identity will remain anonymous but your review may still be made public.

**Do you want your identity to be public for this peer review?**  For information about this choice, including consent withdrawal, please see our Privacy Policy

Reviewer #1: No

Reviewer #2: No

**********

[NOTE: If reviewer comments were submitted as an attachment file, they will be attached to this email and accessible via the submission site. Please log into your account, locate the manuscript record, and check for the action link "View Attachments". If this link does not appear, there are no attachment files.]

While revising your submission, please upload your figure files to the Preflight Analysis and Conversion Engine (PACE) digital diagnostic tool, https://pacev2.apexcovantage.com/. PACE helps ensure that figures meet PLOS requirements. To use PACE, you must first register as a user. Registration is free. Then, login and navigate to the UPLOAD tab, where you will find detailed instructions on how to use the tool. If you encounter any issues or have any questions when using PACE, please email PLOS at figures@plos.org. Please note that Supporting Information files do not need this step.

# Author comment: Author response to Decision Letter 1

**DOI:** 10.1371/journal.pone.0342133.r002

1 Oct 2025

Reviewer 1 comments (review points to consider)

1 The abstract presents clear picture of what readers should expect with the whole work and shows consistency throughout the write-up while also filling a research gap in patient care in eye clinics.

The research appears to be well thought-out with rigorous design demonstrated, consistent referencing and supplementary materials provided.

Sentences are precise and easy to understand despite presenting complex scenarios. The results were clearly presented and shows analytical rigour with the discussion section well-presented and highlighting related studies.

Response:

We would like to thank the reviewer for their positive and generous comments regarding our manuscript and the design of our study.We greatly appreciate their thoughtful feedback and are pleased that the study’s approach and contribution were well received.

2 First, perhaps for non-expert readers, the paper could benefit from a few lines in the introductory section that provides more details of cases where asynchronous-review eye clinics have been successful in other countries if there are no sufficient evidence from the UK.

Response:

We thank the reviewer for this suggestion. We have looked at the earlier iterations of asynchronous eye clinics and in light of this comment have added a few lines highlighting the use of this service model in diabetic retinopathy screening in Sweden, Singapore, and the UK. We state that: “Asynchronous-review clinics are not a new innovation in ophthalmology. Earlier iterations of the model include mobile diabetic retinopathy screening programmes, as introduced in Stockholm, Sweden, in 1990 and in Singapore in 2010 [7]. The UK has one of the largest and most successful diabetic retinopathy screening programmes, where trained technicians capture 45° colour fundus photographs that are later reviewed by clinicians; an approach that contributed to diabetic retinopathy no longer being the leading cause of blindness registration in the UK by 2010, for the first time in 50 years [7].”

3 Second review point is with regards to this statement “All respondents were asked to respond from a patient’s perspective, so that the same language could be used for all, to make the results comparable across respondent types and simplify distribution of the survey link”. While asking all respondents to respond from a patient’s perspective as stated above is justified by the authors in the statement, it is difficult to be certain that respondents that do not fall into the patient category can make choices as patient respondents would since they may not have the experiences in terms of treatment and appointment needs as patients would. Also, even if non-patient respondents responded from patients’ perspective, it could imply that the preferences of non-patient respondents themselves were not really captured for comparisons with the responses of patients. Thus, querying why there was a need to involve non-patient respondents or whether the opinions of non-patient respondents were actually captured by the survey.

Response:

Thank you very much for this clarifying comment.

Firstly, inviting participants to respond from a patient’s perspective enabled us to explore what key stakeholders perceive as fair and valuable to patients.

Secondly, as shown in Tables 4 and 5, as well as Supplementary tables S5 and S7, we conducted extensive subgroup analyses involving eye patients, healthcare professionals (HCPs), and members of the public as part of our sensitivity analyses. For example, we found that the public was statistically indifferent between seeing an optometrist or doctor and receiving same-day results (reference category) versus seeing a technician and receiving results by phone. In contrast, eye patients and HCPs exhibited statistically significant preferences (p < 0.001), with the lowest utility associated with seeing a technician and receiving results by post (S7 Table. Outputs of the conditional logit regression model).

We also report that patients required a reduction of 7.29 months (95% CI: 4.25 to 10.32) to accept seeing a technician and receiving results by post, compared to the reference category. This figure was 7.84 months for HCPs and 4.07 months for the public. Additionally, patients required a reduction in travel time of 305 minutes to accept this trade-off, which was approximately four times greater than the public’s requirement of 73 minutes (Table 5).

Overall, we believe this approach has allowed us to assess what key stakeholders—whether current eye patients, potential future patients, or policy constituents—consider fair to patients. We believe that recognising differences in what patients value versus what stakeholders assume patients value is a key and crucial step in designing truly patient-centred care. To this end, We have also thoroughly examined the heterogeneity of preferences across these three stakeholder groups.

Reviewer 2 comments

1 This research explores alternative treatment options for patients with eye diseases with the involvement of technicians. While this research is promising, it must be cautious because there are many types of eye diseases ranging from mild to severe, and not all can be treated by technicians. The questions in this study are not about general eye treatments, nor are they differentiated or grouped by type of disease. If the eye disease groups were differentiated, the service options would be different.

Response:

We would like to thank the reviewer for this insightful comment and agree that “there are many types of eye diseases ranging from mild to severe, and not all can be treated by technicians.”

Our study focuses on services for patients with stable chronic eye conditions that require regular monitoring and pose low clinical risk. This was made explicit in the survey design. For example, in Section Two of the questionnaire, participants were asked to consider “features of services where patients with a stable long-term eye condition might go for their regular eye check-ups” (S 1. The full survey for the Discrete Choice Experiment).

We intentionally kept the condition description broad to allow for generalisability to other high-volume, low-complexity specialties within the National Health Service (NHS) that also require routine monitoring. The wider HERCULES Project, within which this study is situated, principally focuses on glaucoma and medical retina conditions—both of which account for a substantial proportion of outpatient ophthalmology appointments in NHS England

2 The respondents were divided into three groups: patients, healthcare professionals, and the public. The last two groups could not indicate whether or not they had ever experienced eye pain (this question was not included in the questionnaire). Respondents who have experienced eye pain may differ from those who have not in their choice of services.

Response:

We would like to thank the reviewer for this insightful comment. Indeed, respondents who have experienced eye pain may differ from those who have not in their choice of services. We have now noted this concern as a fourth limitation in our study. We state that, “… there potentially could be underlying heterogeneity in each respondent group that could shape preferences. For example, preferences for non-eye-patient respondents who have experienced eye pain might differ from those who have not. However, this is secondary and would require a very large sample to sufficiently tease out.”

3 The concept of willingness to pay is examined in relation to willingness to wait and willingness to travel. Is there data on how respondents' occupations relate to time expenditure? Those with time-consuming jobs tend to choose short-distance services.

Response:

We thank the reviewer for this insightful comment. In Section 4, Question 10, of our questionnaire, we asked respondents to report their employment status (S 1. The full survey for the Discrete Choice Experiment). As detailed in S 4. Table. Within-group comparison tests, our within-group comparison tests found that that preferences were statistically indifferent between those in full-time employment vs retired vs other employment, retired vs other employment, full time employment vs other employment. Statistically significant different preferences were detected between those in full-time employment vs retired. We do not have any further breakdown on the types of jobs that people had.In response to this comment, we have now added summary data on occupational status to Table 3 to enhance clarity and transparency.

4 If no information is available from the previous point, it can be included as a limitation of this research

Response:

This information has been captured in S 4. Table. Within-group comparison tests.

Attachment Submitted filename: 2025 08 28 Response to comments v2.docx

# Peer review: Decision Letter 1

**DOI:** 10.1371/journal.pone.0342133.r003

**Authors:** Ilse Bloom

10 Feb 2026

Preferences for community-based asynchronous-review eye clinics in the UK: a discrete choice experiment among patients, healthcare professionals, and the public

PLOS One

Dear Dr. Ndwandwe,

Please submit your revised manuscript by Mar 26 2026 11:59PM. If you will need more time than this to complete your revisions, please reply to this message or contact the journal office at plosone@plos.org. When you're ready to submit your revision, log on to https://www.editorialmanager.com/pone/ and select the 'Submissions Needing Revision' folder to locate your manuscript file.

- A letter that responds to each point raised by the academic editor and reviewer(s). You should upload this letter as a separate file labeled 'Response to Reviewers'.
- A marked-up copy of your manuscript that highlights changes made to the original version. You should upload this as a separate file labeled 'Revised Manuscript with Track Changes'.
- An unmarked version of your revised paper without tracked changes. You should upload this as a separate file labeled 'Manuscript'.

If you would like to make changes to your financial disclosure, please include your updated statement in your cover letter. Guidelines for resubmitting your figure files are available below the reviewer comments at the end of this letter.

https://journals.plos.org/plosone/s/submission-guidelines#loc-laboratory-protocols. Additionally, PLOS ONE offers an option for publishing peer-reviewed Lab Protocol articles, which describe protocols hosted on protocols.io. Read more information on sharing protocols at https://plos.org/protocols?utm_medium=editorial-email&utm_source=authorletters&utm_campaign=protocols.

We look forward to receiving your revised manuscript.

Kind regards,

Ilse Bloom

Staff Editor

PLOS One

Journal Requirements:

1. If the reviewer comments include a recommendation to cite specific previously published works, please review and evaluate these publications to determine whether they are relevant and should be cited. There is no requirement to cite these works unless the editor has indicated otherwise.

2. Please review your reference list to ensure that it is complete and correct. If you have cited papers that have been retracted, please include the rationale for doing so in the manuscript text, or remove these references and replace them with relevant current references. Any changes to the reference list should be mentioned in the rebuttal letter that accompanies your revised manuscript. If you need to cite a retracted article, indicate the article’s retracted status in the References list and also include a citation and full reference for the retraction notice.

Additional Editor Comments:

The manuscript has been evaluated by five reviewers, and their comments are available below.

Could you please carefully revise the manuscript to address all comments raised?

[Note: HTML markup is below. Please do not edit.]

Reviewer's Responses to Questions

**Comments to the Author**

Reviewer #1: All comments have been addressed

Reviewer #2: All comments have been addressed

Reviewer #3: All comments have been addressed

Reviewer #4: All comments have been addressed

Reviewer #5: (No Response)

**********

2. Is the manuscript technically sound, and do the data support the conclusions?

Reviewer #1: Yes

Reviewer #2: (No Response)

Reviewer #3: Yes

Reviewer #4: Yes

Reviewer #5: Yes

**********

3. Has the statistical analysis been performed appropriately and rigorously?

Reviewer #1: Yes

Reviewer #2: (No Response)

Reviewer #3: Yes

Reviewer #4: Yes

Reviewer #5: Yes

**********

4. Have the authors made all data underlying the findings in their manuscript fully available?

The PLOS Data policy

Reviewer #1: Yes

Reviewer #2: (No Response)

Reviewer #3: Yes

Reviewer #4: Yes

Reviewer #5: No

**********

5. Is the manuscript presented in an intelligible fashion and written in standard English?

Reviewer #1: Yes

Reviewer #2: (No Response)

Reviewer #3: Yes

Reviewer #4: Yes

Reviewer #5: Yes

**********

Reviewer #1: The authors have addressed all my comments in the first review and have made changes in the article to reflect them.

Reviewer #2: (No Response)

Reviewer #3: 2. Methods Clarification:

>Line 175-176: Please clarify the eligibility criteria used in Section 1 to screen respondents.

>Consistency Checks: You note the absence of consistency/dominance checks as a limitation. Consider briefly discussing in the methods or limitations why this common DCE practice was omitted (e.g., due to cognitive burden concerns from your design work).

3. Results Presentation:

>Figure 1 & Legend: The legend for Figure 1 (lines 278-282) describes results for a different topic ("acceptable mode of appointment") which seems misplaced. The legend should describe the attribute ranking figure itself. The text currently in the legend appears to belong in the Supplementary Materials or a different part of the results (e.g., around Supplementary Part 6).

>Tables 4 & 5: The estimates for Patients in Tables 4 and 5 have very wide confidence intervals (e.g., WTT: -305 (-628, 18) minutes). This should be briefly acknowledged in the text as indicating less precise estimates within this subgroup, potentially due to heterogeneity or sample size.

4. Discussion Refinement:

>Limitations - Socioeconomic Representativeness: The point about homeownership rates is well-taken. Consider also commenting briefly on the overrepresentation of highly educated respondents (60% with a degree) and its potential impact on the generalizability of preferences.

>Interpretation of WTW: The discussion on lines 357-370 about patients "willing to wait... longer to see a clinician" is insightful. To avoid misinterpretation, you could explicitly state that from a service design perspective, the primary utility of the finding is in quantifying the reduction in delay needed to make technician-led models acceptable, not in endorsing longer delays.

5. Language and Copyediting:

>Please perform a thorough proofread to correct the typographical errors, awkward line breaks, and minor grammatical issues noted above and throughout the text. Ensure consistency in table formatting.

Reviewer #4: The exploration of alternative service delivery models in the interest of public health is commendable, especially given the premise that delayed care can equate to denied care. The study is well-designed to assess the trade-offs patients are willing to make, such as balancing travel time against waiting periods for appointments. It is particularly insightful in evaluating patient preferences in the context of technician-led clinics, remote result communication, and reduced appointment delays. Understanding these preferences is crucial for shaping more efficient and accessible healthcare services.

However, there are notable limitations regarding the sample composition. A significant proportion of respondents—approximately 79% of patients and 65% of the general public—were above 55 years of age. This age distribution likely influenced the findings on willingness to wait and to travel, as older individuals may have different preferences and constraints compared to younger populations. Furthermore, the majority of participants were from outside London, where access to healthcare facilities and public transport may differ from larger cities. This could explain the greater acceptance of local technician-led models. If the sample had included a broader age range and more participants from metropolitan areas with robust public transport infrastructure, the acceptance of non-clinician-led care might have yielded different results. Future studies should consider a more heterogeneous sample to enhance the generalisability of the findings. In public health service delivery there is no one shoe that fits all as shown in literature so what works in once place and population group

Recommendation:

Since the study clearly indicated that clinician driven care was the preferred option and sample was skewed more geraitric, it would be good to conclude saying that technician led care of service delivery could be considered as a door step/ close to home care model in remote locations for geriatic population as part of NHS service. And considering that same day results was also a preferred option, this could be coupled with AI or telehealth or MHealth platforms. for expert opinion on the spot.

Reviewer #5: In “Preferences for community-based asynchronous-review eye clinics in the UK: a discrete choice experiment among patients, healthcare professionals, and the public,” Siyabonga Ndwandwe et al. examine the trade-offs that eye-care users are willing to make between care setting and time costs when accessing care for stable eye conditions. The study demonstrates that decentralizing chronic eye-care monitoring from hospitals to community-based settings can help reduce pressure on hospital clinics, among other benefits highlighted in the paper. A particular strength is the stratification of respondents by their interaction with eye-care and healthcare services, which allows the authors to focus on individuals with actual eye-care needs who are most likely to use community-based services.

The authors present a clear research question and apply a robust methodology aligned with their aims. The results are clearly reported and thoughtfully discussed. Overall, the manuscript is well written and makes for an engaging and informative read.

Specific comments

1. The severity of patients' eye conditions likely influences the urgency of their eye care needs and thus their willingness to wait for care or travel to receive it. The authors collected information on the frequency of eye follow-up visits and the receipt of regular intraocular injections. Could the authors clarify how these factors were incorporated into the analysis?

As mentioned in lines 243–244, were similar tests conducted within patient subgroups stratified by frequency of eye care use?

2. The discussion section is brief and does not address all the presented results. A focused discussion of the implications of the subgroup analyses (lines 326–332) would provide valuable insight and strengthen the paper.

**********

what does this mean?). If published, this will include your full peer review and any attached files.

If you choose “no”, your identity will remain anonymous but your review may still be made public.

**Do you want your identity to be public for this peer review?**  For information about this choice, including consent withdrawal, please see our Privacy Policy

Reviewer #1: No

Reviewer #2: No

Reviewer #3: **Yes:** Md Mahmudul Hasan

Reviewer #4: No

Reviewer #5: No

**********

[NOTE: If reviewer comments were submitted as an attachment file, they will be attached to this email and accessible via the submission site. Please log into your account, locate the manuscript record, and check for the action link "View Attachments". If this link does not appear, there are no attachment files.]

To ensure your figures meet our technical requirements, please review our figure guidelines: https://journals.plos.org/plosone/s/figures

You may also use PLOS’s free figure tool, NAAS, to help you prepare publication quality figures: https://journals.plos.org/plosone/s/figures#loc-tools-for-figure-preparation.

NAAS will assess whether your figures meet our technical requirements by comparing each figure against our figure specifications.

# Author comment: Author response to Decision Letter 2

**DOI:** 10.1371/journal.pone.0342133.r004

17 Apr 2026

Responses to peer reviewer comments, April 2026

Reviewer 1 comments: The authors have addressed all my comments in the first review and have made changes in the article to reflect them

Response to Reviewer 1 comments: No further response required

Reviewer 2 comments: (No Response)

Response to Reviewer 2 comments: No further response required

Reviewer 3 comments (methods clarification): Line 175-176: Please clarify the eligibility criteria used in Section 1 to screen respondents.

Response to Reviewer 3 comments: We thank the reviewer for this helpful comment. We have now clarified the eligibility criteria (lines 177-178). The manuscript now also states:

“To be eligible, participants had to be at least 18 years old.”

Reviewer 3 comments (methods clarification): Consistency Checks: You note the absence of consistency/dominance checks as a limitation. Consider briefly discussing in the methods or limitations why this common DCE practice was omitted (e.g., due to cognitive burden concerns from your design work).

Response to Reviewer 3 comments: We thank the reviewer for raising this important point and we have added some discussion on this in the Limitations section (lines 394-402).

Reviewer 3 comments (results presentation): Figure 1 & Legend: The legend for Figure 1 (lines 278-282) describes results for a different topic ("acceptable mode of appointment") which seems misplaced. The legend should describe the attribute ranking figure itself. The text currently in the legend appears to belong in the Supplementary Materials or a different part of the results (e.g., around Supplementary Part 6).

Response to Reviewer 3 comments: We thank the reviewer for identifying this discrepancy. The legend for Figure 1 has now been corrected to accurately describe the attribute ranking figure. The revised legend is:

Figure 1 Legend: “A simple ranking of the five attributes showed that 44% (173/389) felt that the composite attribute describing who patient sees on the day and mode of results communication was the most important and 44% (173/389) felt that parking availability was the least important of the five.”

Reviewer 3 comments (results presentation): Tables 4 & 5: The estimates for Patients in Tables 4 and 5 have very wide confidence intervals (e.g., WTT: -305 (-628, 18) minutes). This should be briefly acknowledged in the text as indicating less precise estimates within this subgroup, potentially due to heterogeneity or sample size.

Response to Reviewer 3 comments: We thank the reviewer for this observation. The wide willingness‑to‑travel (WTT) confidence intervals for the patient subgroup arise because, as shown in supplementary table, S7 Table, travel time was not a statistically significant predictor of utility for this group. We have now explicitly acknowledged this in the Results section (lines 311-314), noting that the imprecision likely reflects a weaker association between travel time and preferences in patients.

Reviewer 3: Discussion Refinement Limitations - Socioeconomic Representativeness: The point about homeownership rates is well-taken. Consider also commenting briefly on the overrepresentation of highly educated respondents (60% with a degree) and its potential impact on the generalizability of preferences. We thank the reviewer for this comment. We have now added a comment on this in the limitations section (lines 408-410).

Reviewer 3 comments (discussion refinement): Interpretation of WTW: The discussion on lines 357-370 about patients "willing to wait... longer to see a clinician" is insightful. To avoid misinterpretation, you could explicitly state that from a service design perspective, the primary utility of the finding is in quantifying the reduction in delay needed to make technician-led models acceptable, not in endorsing longer delays.

Response to Reviewer 3 comments: We thank the reviewer for this helpful comment. We are indeed cautious to avoid any possible misinterpretation, and we have added this further clarification in lines 415-421.

Reviewer 3 comments (language and copyediting): Please perform a thorough proofread to correct the typographical errors, awkward line breaks, and minor grammatical issues noted above and throughout the text. Ensure consistency in table formatting.

Response to Reviewer 3 comments: We thank the reviewer for this comment. We have revised the work and corrected these issues throughout.

Reviewer 4 comments: The exploration of alternative service delivery models in the interest of public health is commendable, especially given the premise that delayed care can equate to denied care. The study is well-designed to assess the trade-offs patients are willing to make, such as balancing travel time against waiting periods for appointments. It is particularly insightful in evaluating patient preferences in the context of technician-led clinics, remote result communication, and reduced appointment delays. Understanding these preferences is crucial for shaping more efficient and accessible healthcare services. However, there are notable limitations regarding the sample composition. A significant proportion of respondents—approximately 79% of patients and 65% of the general public—were above 55 years of age. This age distribution likely influenced the findings on willingness to wait and to travel, as older individuals may have different preferences and constraints compared to younger populations. Furthermore, the majority of participants were from outside London, where access to healthcare facilities and public transport may differ from larger cities. This could explain the greater acceptance of local technician-led models. If the sample had included a broader age range and more participants from metropolitan areas with robust public transport infrastructure, the acceptance of non-clinician-led care might have yielded different results. Future studies should consider a more heterogeneous sample to enhance the generalisability of the findings. In public health service delivery there is no one shoe that fits all as shown in literature so what works in once place and population group

Response to Reviewer 4 comments: We thank the reviewer for these thoughtful observations.

Reviewer 4 comments (recommendation):Since the study clearly indicated that clinician driven care was the preferred option and sample was skewed more geriatric, it would be good to conclude saying that technician led care of service delivery could be considered as a door step/ close to home care model in remote locations for geriatric population as part of NHS service. And considering that same day results was also a preferred option, this could be coupled with AI or telehealth or MHealth platforms. for expert opinion on the spot.

Response to Reviewer 4 comments: We thank the reviewer for these thoughtful comments. On lines 346-352, at the end of the Results section, we described aggregating some variables to attempt to assess some level of heterogeneity across potential subgroups. For example, we collapsed the eight age groups highlighted in table 3 into two groups: respondents aged ≤65 years and those ≥66 years and then compared responses between these two groups, as shown in Table S4, but found no statistically significant difference in direction or magnitude between the two groups. We did not collect information that would allow us to state the relative degree of remoteness of residence. The closest we have for this is collapsing place-of-residence subgroups to London and Out-of-London, which we had also done and which is described similarly in lines 346-349. As Table S4 shows, we found that preferences in these two subgroups had the same direction but statistically different magnitude. We feel that the suggestions made by the reviewer might not be sufficiently evidenced by the analysis and dataset that we have, so we have not added any further comments on this to the manuscript.

The reviewer further suggested that we could consider aligning the results with topical issues like AI and MHealth platforms. We greatly appreciate this consideration; however, as we did not consider such development in the study design we have similarly decided not to add in comments on this. If the Editor feels strongly that we should make some comment along these lines, then we would be happy to discuss this further with them.

Reviewer 5 comments (overview): In “Preferences for community-based asynchronous-review eye clinics in the UK: a discrete choice experiment among patients, healthcare professionals, and the public,” Siyabonga Ndwandwe et al. examine the trade-offs that eye-care users are willing to make between care setting and time costs when accessing care for stable eye conditions. The study demonstrates that decentralizing chronic eye-care monitoring from hospitals to community-based settings can help reduce pressure on hospital clinics, among other benefits highlighted in the paper. A particular strength is the stratification of respondents by their interaction with eye-care and healthcare services, which allows the authors to focus on individuals with actual eye-care needs who are most likely to use community-based services. The authors present a clear research question and apply a robust methodology aligned with their aims. The results are clearly reported and thoughtfully discussed. Overall, the manuscript is well written and makes for an engaging and informative read.

Response to Reviewer 5 comments: We thank the reviewer for these kind comments.

Reviewer 5 comments (specific comments) 1. The severity of patients' eye conditions likely influences the urgency of their eye care needs and thus their willingness to wait for care or travel to receive it. The authors collected information on the frequency of eye follow-up visits and the receipt of regular intraocular injections. Could the authors clarify how these factors were incorporated into the analysis? As mentioned in lines 243–244, were similar tests conducted within patient subgroups stratified by frequency of eye care use?

Response to Reviewer 5 comments: We thank the reviewer for this insightful comment. Our study was however not designed to assess within‑group heterogeneity by disease severity or frequency of eye care use. Such analyses would have required substantially larger subgroups, with sufficient balance. For example, table 3 shows that 82% (n=318) of all respondents’ self-reported health status was good (n=244, 63%) or very good (n=74, 19%), while only 16% (n= 62) of all respondents reported poor or very poor health status. Conducting a within-group analysis with this level of sample imbalance would have yielded imprecise estimates, potentially with very wide confidence intervals. In some instances, within-group sample imbalance would have limited convergence of the conditional logit model: for example, Table S11 shows that 100% of the 21 respondents living with long-term and short-term eye conditions were patients, therefore we could not go into this level of detail in the analysis by type of respondent.

We have clarified the mention of this limitation in the Limitations section and we hope that it reads more clearly.

Reviewer 5 comments (discussion): The discussion section is brief and does not address all the presented results. A focused discussion of the implications of the subgroup analyses (lines 326–332) would provide valuable insight and strengthen the paper.

Response to Reviewer 5 comments: We thank the reviewer for this valuable suggestion. However, we intentionally avoided over‑interpreting results due to sample size constraints—the study was not powered to detect within-group differences beyond the three broad type of respondents. We have added a comment in the discussion to state that the small sample size precludes further discussion here on these points.

Attachment Submitted filename: 2026 04 16 Responses to reviewers.docx

# Peer review: Decision Letter 2

**DOI:** 10.1371/journal.pone.0342133.r005

**Authors:** Taiwo Opeyemi Aremu

25 Jun 2026

Dear Dr. Ndwandwe,

Thank you for submitting your manuscript to PLOS ONE. After careful consideration, we feel that it has merit but does not fully meet PLOS ONE’s publication criteria as it currently stands. Therefore, we invite you to submit a revised version of the manuscript that addresses the points raised during the review process.

Please submit your revised manuscript by Aug 09 2026 11:59PM. If you will need more time than this to complete your revisions, please reply to this message or contact the journal office at plosone@plos.org. When you're ready to submit your revision, log on to https://www.editorialmanager.com/pone/ and select the 'Submissions Needing Revision' folder to locate your manuscript file.

- A letter that responds to each point raised by the academic editor and reviewer(s). You should upload this letter as a separate file labeled 'Response to Reviewers'.
- A marked-up copy of your manuscript that highlights changes made to the original version. You should upload this as a separate file labeled 'Revised Manuscript with Track Changes'.
- An unmarked version of your revised paper without tracked changes. You should upload this as a separate file labeled 'Manuscript'.

If you would like to make changes to your financial disclosure, please include your updated statement in your cover letter. Guidelines for resubmitting your figure files are available below the reviewer comments at the end of this letter.

If applicable, we recommend that you deposit your laboratory protocols in protocols.io to enhance the reproducibility of your results. Protocols.io assigns your protocol its own identifier (DOI) so that it can be cited independently in the future. For instructions see: https://journals.plos.org/plosone/s/submission-guidelines#loc-laboratory-protocols. Additionally, PLOS ONE offers an option for publishing peer-reviewed Lab Protocol articles, which describe protocols hosted on protocols.io. Read more information on sharing protocols at https://plos.org/protocols?utm_medium=editorial-email&utm_source=authorletters&utm_campaign=protocols.

As the corresponding author, your ORCID iD is verified in the submission system and will appear in the published article. PLOS supports the use of ORCID, and we encourage all coauthors to register for an ORCID iD and use it as well. Please encourage your coauthors to verify their ORCID iD within the submission system before final acceptance, as unverified ORCID iDs will not appear in the published article. *Only*  the individual author can complete the verification step; PLOS staff *cannot*  verify ORCID iDs on behalf of authors.

We look forward to receiving your revised manuscript.

Kind regards,

Taiwo Opeyemi Aremu, MD, MPH, PhD

Academic Editor

PLOS One

Journal Requirements:

1. If the reviewer comments include a recommendation to cite specific previously published works, please review and evaluate these publications to determine whether they are relevant and should be cited. There is no requirement to cite these works unless the editor has indicated otherwise.

2. Please review your reference list to ensure that it is complete and correct. If you have cited papers that have been retracted, please include the rationale for doing so in the manuscript text, or remove these references and replace them with relevant current references. Any changes to the reference list should be mentioned in the rebuttal letter that accompanies your revised manuscript. If you need to cite a retracted article, indicate the article’s retracted status in the References list and also include a citation and full reference for the retraction notice.

[Note: HTML markup is below. Please do not edit.]

Reviewers' comments:

Reviewer's Responses to Questions

**Comments to the Author**

Reviewer #1: All comments have been addressed

Reviewer #3: (No Response)

Reviewer #4: All comments have been addressed

Reviewer #5: All comments have been addressed

**********

2. Is the manuscript technically sound, and do the data support the conclusions?

Reviewer #1: Yes

Reviewer #3: Yes

Reviewer #4: Yes

Reviewer #5: Yes

**********

3. Has the statistical analysis been performed appropriately and rigorously?

Reviewer #1: Yes

Reviewer #3: Yes

Reviewer #4: Yes

Reviewer #5: Yes

**********

4. Have the authors made all data underlying the findings in their manuscript fully available?

The PLOS Data policy

Reviewer #1: Yes

Reviewer #3: No

Reviewer #4: Yes

Reviewer #5: (No Response)

**********

5. Is the manuscript presented in an intelligible fashion and written in standard English?

Reviewer #1: Yes

Reviewer #3: Yes

Reviewer #4: Yes

Reviewer #5: Yes

**********

Reviewer #1: (No Response)

Reviewer #3: Major Strengths

>Unique contribution: This is the first DCE to explicitly test preference for a technician-led asynchonous-review model of ophthalmology care, filling in a critical knowledge gap for implementation science of workforce-efficient clinical models.

>Sound methodology: The D-optimal design, forced-choice, and choice between dominance checks (justified for reasons of cognitive load) are thoughtful choices.

>Relevant clinical insights: The core finding that respondents would accept technician-led care if appointment wait times were cut by 3-6 months (or time travelling to the appointment by 1-2.5 hours) offers useful insights for service planners.

Major Concerns to Address

1. Generalisability

>The authors note some substantial demographic bias (79% homeowners vs. national average of 62.5%; 60% college degree vs. national average of 33%), but this should be emphasised in the abstract and conclusions. These factors are known to affect preferences for health-care services and may affect generalizability to socioeconomically more diverse communities. The authors should consider:

>Including a statement of limitations in the abstract

>Including more detail on how these may impact the WTW/WTT estimates (e.g., higher education is associated with stronger preferences for more contact with health care providers?

2. Inconsistent patient vs. public perspective

>All respondents (including HCPs and the public) were asked to answer "from a patient's perspective". This approach is problematic for the following reasons:

>HCPs may find it challenging to put aside their own beliefs and thoughts, creating a response bias

>The members of the public without chronic eye disease lack the experience needed to develop preferences

>These are two different groups (subgroup analyses) but still asked to take on a patient perspective

Suggestion: This should be explained and defended, or the authors should report it as a weakness. Perhaps the use of independent models (patient vs. reference) could be justified, rather than having them all think in a certain way.

3. Unrealistic WTT estimates range beyond attributes

The authors acknowledge the estimated WTTs (e.g., 305 minutes for patients who would accept technician+post vs. reference) are larger than the largest travel times presented in the DCE (90+ minutes). They refer to other data, but this implies extrapolations. The authors should:

>Clearly report these estimates are extrapolations and should be used carefully

>Explore the possibility of a non-linear utility function in modelling travel time values

>Include the proportion of respondents who had WTT values higher than the highest attribute level

4. Handling of missing data and completion rate

The authors say "rates of missing data were very low" but this is not quantified. An average of 14.5 minutes (SD 26.3) to complete had a long tail, with some taking a long time. The authors should:

>Include the survey completion rate (percentage of the survey started completed)

>Report the number of people who initiated but did not complete the survey

>Disclose rates of missing data for key variables

5. Composite attribute interpretability

The composite attribute ("who the patient sees") and ("how the results are given") is justified but causes interpretational problems. The bundle "clinician + same-day results" is different to the other bundles (technician + phone or technician + post) in two ways. This means the WTW estimates bundle:

>The disutility of technician (not clinician)

>The disutility of phone/post vs. in-person communication

>The disutility of phone/post vs. face-to-face

>The authors note this but consider whether changing the design (e.g., separate attributes with interactions) may have allowed for decomposition of this.

Minor Concerns and Clarifications Needed

6. Table size: Table 3 is long. Move data to the supplement or reduce categories.

7. Interpretation of confidence intervals: For patients WTT estimates (Table 5), 95% CI for technician+post (-628 to 18) includes zero and is very large, corresponding to the non-significant travel time coefficient in S7. This is noted, but should be made clearer that they are statistically unreliable and should not be misinterpreted.

8. Opportunity for more with attitudinal data: Section 4 involved attitudinal data collection but not in the main findings. Could any insights be added from the attitudinal data (e.g. relationship between trust in technicians and preferences).

9. Conflicts of interest: Multiple authors (PJF, SS, PTK, HJ) have multiple industry ties. These are all declared but readers should remain alert to the influence of sponsorship (this is less likely in a DCE).

Questions for Authors

>Did you investigate preference heterogeneity (mixed logit and latent class)? If yes, please provide estimates. If not, explain why on not needed conditional logit.

>How did you check that the composite attribute was understood? Were checks made using the longer descriptions?

>Since 49% of patients used public transport to attend appointments, but only 19% of the public would be willing to do so, does this indicate hypothetical bias in the public's responses from adopting a patient perspective?

>The fact that schedules are delayed implicitly and by implication patients would wait 6 months for appointments (lines 378-380) appears to imply a change in direction of causality for the WTW estimate. Please note that the estimate is the compensation needed for patients to accept care from a technician, not a preference for a longer wait.

Recommendation

Minor Revision - The manuscript is well done and a valuable contribution but needs to address the need for representativeness, for providing care from a patient's perspective, and explain the WTT concerns.

Reviewer #4: The authors have tried to incorporate the comments with the available data. And I have no further comments for them.

Reviewer #5: (No Response)

**********

what does this mean?). If published, this will include your full peer review and any attached files.

If you choose “no”, your identity will remain anonymous but your review may still be made public.

**Do you want your identity to be public for this peer review?**  For information about this choice, including consent withdrawal, please see our Privacy Policy

Reviewer #1: No

Reviewer #3: **Yes:** Md Mahmudul Hasan

Reviewer #4: No

Reviewer #5: No

**********

[NOTE: If reviewer comments were submitted as an attachment file, they will be attached to this email and accessible via the submission site. Please log into your account, locate the manuscript record, and check for the action link "View Attachments". If this link does not appear, there are no attachment files.]

To ensure your figures meet our technical requirements, please review our figure guidelines: https://journals.plos.org/plosone/s/figures

You may also use PLOS’s free figure tool, NAAS, to help you prepare publication quality figures: https://journals.plos.org/plosone/s/figures#loc-tools-for-figure-preparation.

NAAS will assess whether your figures meet our technical requirements by comparing each figure against our figure specifications.

# Author comment: Author response to Decision Letter 3

**DOI:** 10.1371/journal.pone.0342133.r006

12 Aug 2026

Responses to peer reviewer comments, July 2026

Reviewers’ comments Authors’ responses

Reviewer #1: All comments have been addressed: No further response required

Reviewer #3: (No Response): No further response required

Reviewer #4: All comments have been addressed: No further response required

Reviewer #5: All comments have been addressed: No further response required

2. Is the manuscript technically sound, and do the data support the conclusions?

The manuscript must describe a technically sound piece of scientific research with data that supports the conclusions. Experiments must have been conducted rigorously, with appropriate controls, replication, and sample sizes. The conclusions must be drawn appropriately based on the data presented.

Reviewer #1: Yes No further response required

Reviewer #3: Yes No further response required

Reviewer #4: Yes No further response required

Reviewer #5: Yes No further response required

3. Has the statistical analysis been performed appropriately and rigorously?

Reviewer #1: Yes No further response required

Reviewer #3: Yes No further response required

Reviewer #4: Yes No further response required

Reviewer #5: Yes No further response required

4. Have the authors made all data underlying the findings in their manuscript fully available?

The PLOS Data policy requires authors to make all data underlying the findings described in their manuscript fully available without restriction, with rare exception (please refer to the Data Availability Statement in the manuscript PDF file). The data should be provided as part of the manuscript or its supporting information, or deposited to a public repository. For example, in addition to summary statistics, the data points behind means, medians and variance measures should be available. If there are restrictions on publicly sharing data—e.g. participant privacy or use of data from a third party—those must be specified.

Reviewer #1: Yes No further response required

Reviewer #3: No

Response:

We thank the reviewer for noting this. We have provided an accompanying data availability statement as part of additional information on the submission portal, where we state that:

“Due to the UK’s national legislative framework (UK GDPR and UK Data Protection Act 2018), institutional data governance arrangements (Moorfields Eye Hospital NHS Foundation Trust and University College London) and ethical considerations (North East - York Research Ethics Committee; Reference 21/NE/0164), the datasets generated and/or analysed during the study cannot be made universally publicly available. Some components of the dataset contain confidential patient information. However, bona fide requests for collaborative access to anonymised data will be considered on a case-by-case basis upon reasonable request to the corresponding author and in accordance with standard data sharing regulations and policies of the study sponsor. Initial enquiries should be directed to the Research Governance Lead, Moorfields Eye Hospital NHS Foundation Trust (moorfields.resadmin@nhs.net).

Some of our data is available and has been provided via supplementary information files, titled 'Supplementary materials.zip”

Reviewer #4: Yes No further response required

Reviewer #5: (No Response) No further response required

5. Is the manuscript presented in an intelligible fashion and written in standard English?

PLOS ONE does not copyedit accepted manuscripts, so the language in submitted articles must be clear, correct, and unambiguous. Any typographical or grammatical errors should be corrected at revision, so please note any specific errors here.

Reviewer #1: Yes No further response required

Reviewer #3: Yes No further response required

Reviewer #4: Yes No further response required

Reviewer #5: Yes No further response required

6. Review Comments to the Author

Please use the space provided to explain your answers to the questions above. You may also include additional comments for the author, including concerns about dual publication, research ethics, or publication ethics. (Please upload your review as an attachment if it exceeds 20,000 characters)

Reviewer #1: (No Response) No further response required

Reviewer #3: Major Strengths

>Unique contribution: This is the first DCE to explicitly test preference for a technician-led asynchonous-review model of ophthalmology care, filling in a critical knowledge gap for implementation science of workforce-efficient clinical models.

>Sound methodology: The D-optimal design, forced-choice, and choice between dominance checks (justified for reasons of cognitive load) are thoughtful choices.

>Relevant clinical insights: The core finding that respondents would accept technician-led care if appointment wait times were cut by 3-6 months (or time travelling to the appointment by 1-2.5 hours) offers useful insights for service planners.

Response: We thank the reviewer for their positive and encouraging assessment of our study. We appreciate the recognition of the study's novelty, methodological rigour, and practical relevance to health planning and policy.

Major Concerns to Address

1. Generalisability

>The authors note some substantial demographic bias (79% homeowners vs. national average of 62.5%; 60% college degree vs. national average of 33%), but this should be emphasised in the abstract and conclusions. These factors are known to affect preferences for health-care services and may affect generalizability to socioeconomically more diverse communities. The authors should consider:

>Including a statement of limitations in the abstract

>Including more detail on how these may impact the WTW/WTT estimates (e.g., higher education is associated with stronger preferences for more contact with health care providers?

Response:

We thank the reviewer for this comment. In response, we have added a statement in the abstract acknowledging this limitation and expanded the discussion in the limitations section. Specifically, in lines 425 - 428, we note that socioeconomic factors and other unobserved respondent characteristics may influence preferences for healthcare delivery models. This would consequently affect the estimated willingness-to-wait (WTW) and willingness-to-travel (WTT) values. However, the available sample size did not support sufficiently powered subgroup analyses to examine these effects formally. As a result, we are unable to isolate the influence these factors might have on preferences and WWT/WTT estimates. Future research using a larger, more socioeconomically diverse sample would be valuable for better understanding how patient characteristics shape preferences for asynchronous, technician-led ophthalmology care.

2. Inconsistent patient vs. public perspective

All respondents (including HCPs and the public) were asked to answer "from a patient's perspective". This approach is problematic for the following reasons:

>HCPs may find it challenging to put aside their own beliefs and thoughts, creating a response bias

>The members of the public without chronic eye disease lack the experience needed to develop preferences

>These are two different groups (subgroup analyses) but still asked to take on a patient perspective

Suggestion: This should be explained and defended, or the authors should report it as a weakness. Perhaps the use of independent models (patient vs. reference) could be justified, rather than having them all think in a certain way.

Response:

We thank the reviewer for this important observation. Our objective was to understand what different stakeholder groups, including healthcare professionals (HCPs) and members of the public, consider acceptable and appropriate for patients, given their important role in the implementation and support of new care models.

In response, we have justified the selected approach as both methodological and practical. In lines 213 – 220, we state that:

“Using a single patient-focused questionnaire across all respondent groups ensured consistency in attribute framing, wording, and choice tasks, reducing the risk that differences in responses would be driven by variations in survey design rather than underlying preferences. It also enabled direct comparison of how different stakeholder groups perceived patient preferences within a common decision-making framework; understanding the gap between what patients value and what stakeholders assume patients value is a key and crucial step in designing truly patient-centred care.”

3. Unrealistic WTT estimates range beyond attributes

The authors acknowledge the estimated WTTs (e.g., 305 minutes for patients who would accept technician+post vs. reference) are larger than the largest travel times presented in the DCE (90+ minutes). They refer to other data, but this implies extrapolations. The authors should:

>Clearly report these estimates are extrapolations and should be used carefully

>Explore the possibility of a non-linear utility function in modelling travel time values

>Include the proportion of respondents who had WTT values higher than the highest attribute level

Reponse:

We thank the reviewer for this important observation. Firstly, we agree that our WTT estimates amongst patients extend beyond the range of travel times presented in the DCE; therefore, they should be interpreted with caution. This might be due to the wide WTT confidence intervals “because travel time did not significantly influence their preferences (see S7 file)”, (lines 352 -354). We further note in the discussion (lines 438 - 440) that this result is consistent with other findings in the literature (Lu et al., 2019).

Secondly, we want to note that WTT estimates are derived from average marginal rates of substitution; therefore, it is not possible, given the statistical software used, to determine the proportion of respondents with values exceeding the highest attribute level presented in the experiment.

Thirdly, we thank the reviewer for suggesting that we explore non-linear specifications of travel time. However, our study design was not sufficient to robustly estimate and compare more complex functional forms. This would be a welcome consideration in future studies.

Lastly, we thank the reviewer for suggesting that we include the proportion of respondents who had WTT values higher than the highest attribute level. However, the WTT estimates are derived from average marginal rates of substitution, and it is not possible, given the statistical software used, to determine the proportion of respondents with values exceeding the highest attribute level presented in the experiment.

4. Handling of missing data and completion rate

The authors say "rates of missing data were very low" but this is not quantified. An average of 14.5 minutes (SD 26.3) to complete had a long tail, with some taking a long time. The authors should:

>Include the survey completion rate (percentage of the survey started completed)

>Report the number of people who initiated but did not complete the survey

>Disclose rates of missing data for key variables

Response:

We thank the reviewer for this comment and suggestions. All variables with missing values are reported in Table 3 and also in S6 Table, which reports respondents' attitudes. To improve clarity, we have revised the methods section to contextualise missingness. We state in lines 183 - 184 that sections 1 to 3 were required to be completed before progressing through the survey. This ensured that there was no missingness on key components of the survey.

On the other hand, the survey was administered and hosted by a third-party survey company, as described in the methods section (lines 168 – 169). Consequently, we do not have information on the number or characteristics of individuals who may have started but not completed the survey, as only completed responses were provided to the research team. We have now clarified this in the methods section, in lines 202 - 207. Therefore, we do not have the information on non-completers to carry out the reviewer’s suggestions.

5. Composite attribute interpretability

The composite attribute ("who the patient sees") and ("how the results are given") is justified but causes interpretational problems. The bundle "clinician + same-day results" is different to the other bundles (technician + phone or technician + post) in two ways. This means the WTW estimates bundle:

>The disutility of technician (not clinician)

>The disutility of phone/post vs. in-person communication

>The disutility of phone/post vs. face-to-face

>The authors note this but consider whether changing the design (e.g., separate attributes with interactions) may have allowed for decomposition of this.

Response:

We thank the reviewer for this insightful comment. The composite attribute was intentionally designed to reflect the real-world service delivery model, where the type of healthcare professional and method of communicating results are experienced jointly by patients. Our objective was therefore to value these service configurations as they occur in practice, rather than to decompose the separate effects of clinician type and result communication method. We have clarified this rationale in lines 151-154 of the manuscript.

Minor Concerns and Clarifications Needed

6. Table size: Table 3 is long. Move data to the supplement or reduce categories.

Response:

We thank the reviewer for this suggestion. However, we have retained the full table because all categories are relevant to interpreting the study findings and provide a complete picture of the sample, including the extent and pattern of missing data. Reducing categories or moving these data to the supplementary material may limit transparency and make it more difficult for readers to fully assess the study population. We would, however, be happy to move this table to the supplementary information if the editor feels that would be preferable.

7. Interpretation of confidence intervals: For patients WTT estimates (Table 5), 95% CI for technician+post (-628 to 18) includes zero and is very large, corresponding to the non-significant travel time coefficient in S7. This is noted, but should be made clearer that they are statistically unreliable and should not be misinterpreted.

ResponseL: We thank the reviewer for this helpful observation. We agree that the WTT estimate for the technician + post scenario should be interpreted with caution. As the reviewer has noted, we have stated that the 95% confidence interval is wide and includes zero, reflecting considerable uncertainty arising from the non-significant travel time coefficient. We have clarified this in the results (lines 352 - 354) and discussion sections (lines 434 - 452)

8. Opportunity for more with attitudinal data: Section 4 involved attitudinal data collection but not in the main findings. Could any insights be added from the attitudinal data (e.g. relationship between trust in technicians and preferences).

Response:

We thank the reviewer for this comment. As highlighted in the previous round of reviews, our study was not designed to assess within group heterogeneity by disease severity or frequency of eye care use. Such analyses would have required substantially larger subgroups, with sufficient balance. Conducting a within-group analysis with this level of sample imbalance would have yielded imprecise estimates, potentially with very wide confidence intervals. In some instances, within-group sample imbalance would have limited convergence of the conditional logit model. In lines 434 - 440 in the discussion section, we state that this level of detailed assessment, beyond the exploratory analyses presented in the Supplementary Materials, would require a larger sample size.

9. Conflicts of interest: Multiple authors (PJF, SS, PTK, HJ) have multiple industry ties. These are all declared but readers should remain alert to the influence of sponsorship (this is less likely in a DCE).

Response:

We thank the reviewer for this comment. We assure the reviewer and PLOS ONE readership that all relevant competing interests and industry affiliations have been fully disclosed in accordance with journa

Attachment Submitted filename: 2026 08 11 Responses to reviewers.docx

# Peer review: Decision Letter 3

**DOI:** 10.1371/journal.pone.0342133.r007

**Authors:** Taiwo Opeyemi Aremu

25 Aug 2026

Preferences for community-based asynchronous-review eye clinics in the UK: a discrete choice experiment among patients, healthcare professionals, and the public

PONE-D-25-25800R3

Dear Dr. Ndwandwe,

We’re pleased to inform you that your manuscript has been judged scientifically suitable for publication and will be formally accepted for publication once it meets all outstanding technical requirements.

Within one week, you’ll receive an e-mail detailing the required amendments. When these have been addressed, you’ll receive a formal acceptance letter and your manuscript will be scheduled for publication.

An invoice will be generated when your article is formally accepted. Please note, if your institution has a publishing partnership with PLOS and your article meets the relevant criteria, all or part of your publication costs will be covered. Please make sure your user information is up-to-date by logging into Editorial Manager at Editorial Manager® and clicking the ‘Update My Information' link at the top of the page. For questions related to billing, please contact billing support.

If your institution or institutions have a press office, please notify them about your upcoming paper to help maximize its impact. If they’ll be preparing press materials, please inform our press team as soon as possible -- no later than 48 hours after receiving the formal acceptance. Your manuscript will remain under strict press embargo until 2 pm Eastern Time on the date of publication. For more information, please contact onepress@plos.org.

Kind regards,

Taiwo Opeyemi Aremu, MD, MPH, PhD

Academic Editor

PLOS One

Additional Editor Comments (optional):

Reviewers' comments:

Reviewer's Responses to Questions

**Comments to the Author**

Reviewer #3: All comments have been addressed

**********

2. Is the manuscript technically sound, and do the data support the conclusions?

Reviewer #3: Yes

**********

3. Has the statistical analysis been performed appropriately and rigorously?

Reviewer #3: Yes

**********

4. Have the authors made all data underlying the findings in their manuscript fully available?

The PLOS Data policy

Reviewer #3: Yes

**********

5. Is the manuscript presented in an intelligible fashion and written in standard English?

Reviewer #3: Yes

**********

Reviewer #3: (No Response)

**********

what does this mean?). If published, this will include your full peer review and any attached files.

If you choose “no”, your identity will remain anonymous but your review may still be made public.

**Do you want your identity to be public for this peer review?**  For information about this choice, including consent withdrawal, please see our Privacy Policy

Reviewer #3: **Yes:** Md Mahmudul Hasan

**********

# Editor report: Acceptance letter

**DOI:** 10.1371/journal.pone.0342133.r008

**Authors:** Taiwo Opeyemi Aremu

PONE-D-25-25800R3

PLOS One

Dear Dr. Ndwandwe,

I'm pleased to inform you that your manuscript has been deemed suitable for publication in PLOS One. Congratulations! Your manuscript is now being handed over to our production team.

At this stage, our production department will prepare your paper for publication. This includes ensuring the following:

* All references, tables, and figures are properly cited

* All relevant supporting information is included in the manuscript submission,

* There are no issues that prevent the paper from being properly typeset

You will receive further instructions from the production team, including instructions on how to review your proof when it is ready. Please keep in mind that we are working through a large volume of accepted articles, so please give us a few days to review your paper and let you know the next and final steps.

Lastly, if your institution or institutions have a press office, please let them know about your upcoming paper now to help maximize its impact. If they'll be preparing press materials, please inform our press team within the next 48 hours. Your manuscript will remain under strict press embargo until 2 pm Eastern Time on the date of publication. For more information, please contact onepress@plos.org.

You will receive an invoice from PLOS for your publication fee after your manuscript has reached the completed accept phase. If you receive an email requesting payment before acceptance or for any other service, this may be a phishing scheme. Learn how to identify phishing emails and protect your accounts at https://explore.plos.org/phishing.

If we can help with anything else, please email us at customercare@plos.org.

Thank you for submitting your work to PLOS One and supporting open access.

Kind regards,

PLOS One Editorial Office Staff

on behalf of

Dr. Taiwo Opeyemi Aremu

Academic Editor

PLOS One
